# A whole-brain voxel-based analysis of structural abnormalities in PTSD: An ENIGMA-PGC study

**DOI:** 10.1192/j.eurpsy.2025.10062

**Published:** 2025-07-22

**Authors:** Cheryl R.Z. See, Shuqing Si, C. Lexi Baird, Courtney C. Haswell, Ahmed Hussain, Miranda Olff, Dick J. Veltman, Jessie L. Frijling, Mirjam van Zuiden, Saskia B.J. Koch, Laura Nawijn, Li Wang, Ye Zhu, Gen Li, Yuval Neria, Xi Zhu, Benjamin Suarez-Jimenez, Sigal Zilcha-Mano, Amit Lazarov, Jennifer S. Stevens, Kerry Ressler, Negar Fani, Tanja Jovanovic, Sanne J.H. van Rooij, Milissa L. Kaufman, Lauren A.M. Lebois, Isabelle M. Rosso, Elizabeth A. Olson, Justin T. Baker, Scott R. Sponheim, Seth G. Disner, Nicholas D. Davenport, Amit Etkin, Adi Maron-Katz, Murray B. Stein, Martha E. Shenton, Dan J. Stein, Jonathan Ipser, Sheri-Michelle Koopowitz, Soraya Seedat, Stefan du Plessis, Leigh L. van den Heuvel, Shmuel Lissek, Hannah Berg, Thomas Straube, David Hofman, Lee A. Baugh, Gina L. Forster, Raluca M. Simons, Jeffrey S. Simons, Vincent A. Magnotta, Kelene A. Fercho, Xin Wang, Andrew S. Cotton, Erin N. O’Leary, Hong Xie, Daniel W. Grupe, Jack B. Nitschke, Richard J. Davidson, Christine L. Larson, Terri A. deRoon-Cassini, Carissa W. Tomas, Jacklynn M. Fitzgerald, Jennifer Urbano Blackford, Bunmi O. Olatunji, Evan M. Gordon, Geoffrey May, Steven M. Nelson, Ruth Lanius, Jean Théberge, Maria Densmore, Richard W.J. Neufeld, Chadi G. Abdallah, Christopher L. Averill, Ilan Harpaz-Rotem, Ifat Levy, John H. Krystal, Elbert Geuze, Remko van Lutterveld, Emily L. Dennis, David F. Tate, David X. Cifu, William C. Walker, Elisabeth A. Wilde, Nic J.A. van der Wee, Robert R.J.M. Vermeiren, Steven J.A. van der Werff, Katie McLaughlin, Kelly Sambrook, Matthew Peverill, Joaquim Radua, Lauren E. Salminen, Neda Jahanshad, Sophia I. Thomopoulos, Anthony James, Lucia Valmaggia, Paul M. Thompson, Rajendra A. Morey, Matthew J. Kempton

**Affiliations:** 1 Department of Psychosis, Institute of Psychiatry, Psychology & Neuroscience, King’s College London, London, UK; 2 Brain Imaging and Analysis Center, Duke University, Durham, NC, USA; 3 Department of Veteran Affairs Mid-Atlantic Mental Illness Research, Education and Clinical Center, Durham, NC, USA; 4 Department of Psychiatry, Amsterdam University Medical Center, Amsterdam, The Netherlands; 5 ARQ National Psychotrauma Centre, Diemen, The Netherlands; 6 De Viersprong Mental Health Specialist in Personality Disorders, Family and Behavior, Amsterdam, The Netherlands; 7 Clinical Psychology, Utrecht University, Utrecht, The Netherlands; 8 Donders Center for Cognitive Neuroimaging, Radboud University Nijmegen, Nijmegen, The Netherlands; 9 Institute of Psychology, Clinical Psychology, Leiden University, Leiden, The Netherlands; 10 Laboratory for Traumatic Stress Studies, Chinese Academy of Sciences Key Laboratory of Mental Health, Institute of Psychology, Chinese Academy of Sciences, Beijing, China; 11 Department of Psychology, University of Chinese Academy of Sciences, Beijing, China; 12 Center for Global Health Equity, New York University Shanghai, Shanghai, China; 13 Department of Psychiatry, Columbia University Medical Center, New York, NY, USA; 14 Department of Psychiatry, New York State Psychiatric Institute, New York, NY, USA; 15 Department of Neuroscience, University of Rochester Medical Center, Rochester, NY, USA; 16 Department of Psychology, University of Haifa, Haifa, Israel; 17 School of Psychological Sciences, Tel-Aviv University, Tel-Aviv, Israel; 18 Department of Psychiatry and Behavioral Sciences, Emory University School of Medicine, Atlanta, GA, USA; 19 Division of Depression and Anxiety Disorders, McLean Hospital, Belmont, MA, USA; 20 Department of Psychiatry, Harvard Medical School, Boston, MA, USA; 21 Department of Psychiatry and Behavioral Neuroscience, Wayne State University School of Medicine, Detroit, MI, USA; 22 Division of Women’s Mental Health, McLean Hospital, Belmont, MA, USA; 23 Center for Depression, Anxiety, and Stress Research, McLean Hospital, Belmont, MA, USA; 24 Institute for Technology in Psychiatry, McLean Hospital, Belmont, MA, USA; 25 Minneapolis VA Health Care System, Minneapolis, MN, USA; 26 Department of Psychiatry and Behavioral Sciences, University of Minnesota, Minneapolis, MN, USA; 27 Department of Psychiatry and Behavioral Sciences, Stanford University, Stanford, CA, USA; 28 VA Palo Alto Health Care System, Palo Alto, CA, USA; 29 Department of Psychiatry, University of California San Diego, La Jolla, CA, USA; 30 Department of Psychiatry, Neuroimaging Laboratory, Brigham and Women’s Hospital and Harvard Medical School, Boston, MA, USA; 31 Department of Radiology, Brigham and Women’s Hospital, Boston, MA, USA; 32 Department of Psychiatry, Massachusetts General Hospital and Harvard Medical School, Boston, MA, USA; 33 South African Medical Research Council Unit on Risk & Resilience in Mental Disorders, Department of Psychiatry and Neuroscience Institute, University of Cape Town, Cape Town, South Africa; 34 Department of Psychiatry, Stellenbosch University, Stellenbosch, South Africa; 35 South African Medical Research Council Unit on the Genomics of Brain Disorders (GBD), Department of Psychiatry, Stellenbosch University, Stellenbosch, South Africa; 36 Department of Psychology, University of Minnesota, Minneapolis, MN, USA; 37 Institute of Medical Psychology and Systems Neuroscience, University of Münster, Münster, Germany; 38 Division of Basic Biomedical Sciences, Sanford School of Medicine, University of South Dakota, Vermillion, SD, USA; 39 Center for Brain and Behavior Research, University of South Dakota, Vermillion, SD, USA; 40 Sioux Falls VA Health Care System, Sioux Falls, SD, USA; 41 Brain Health Research Centre, Department of Anatomy, University of Otago, Dunedin, New Zealand; 42 Department of Psychology, University of South Dakota, Vermillion, SD, USA; 43 Disaster Mental Health Institute, University of South Dakota, Vermillion, SD, USA; 44 Departments of Radiology, Psychiatry, and Biomedical Engineering, University of Iowa, Iowa City, IA, USA; 45 Civil Aerospace Medical Institute, US Federal Aviation Administration, Oklahoma City, OK, USA; 46 Department of Psychiatry, University of Toledo, Toledo, OH, USA; 47 Department of Neurosciences, University of Toledo, Toledo, OH, USA; 48 Center for Healthy Minds, University of Wisconsin-Madison, Madison, WI, USA; 49 Department of Psychiatry, University of Wisconsin-Madison, Madison, WI, USA; 50 Department of Psychology, University of Wisconsin-Madison, Madison, WI, USA; 51 Department of Psychology, University of Wisconsin-Milwaukee, Milwaukee, WI, USA; 52 Division of Trauma and Acute Care Surgery, Department of Surgery, Medical College of Wisconsin, Milwaukee, WI, USA; 53 Comprehensive Injury Center, Medical College of Wisconsin, Milwaukee, WI, USA; 54 Division of Epidemiology and Social Sciences, Institute of Health and Equity, Medical College of Wisconsin Milwaukee, Milwaukee, WI, USA; 55 Department of Psychology, Marquette University, Milwaukee, WI, USA; 56 Munroe-Meyer Institute, University of Nebraska Medical Center, Omaha, NE, USA; 57 Department of Psychiatry and Behavioral Sciences, Vanderbilt University Medical Center, Nashville, TN, USA; 58 Department of Psychology, Vanderbilt University, Nashville, TN, USA; 59 Department of Radiology, Washington University School of Medicine, St. Louis, MO, USA; 60 Veterans Integrated Service Network-17 Center of Excellence for Research on Returning War Veterans, Waco, TX, USA; 61 Department of Psychology and Neuroscience, Baylor University, Waco, TX, USA; 62 Center for Vital Longevity, School of Behavioral and Brain Sciences, University of Texas at Dallas, Dallas, TX, USA; 63 Department of Psychiatry and Behavioral Science, Texas A&M University Health Science Center, Bryan, TX, USA; 64 Masonic Institute for the Developing Brain, University of Minnesota, Minneapolis, MN, USA; 65 Department of Pediatrics, University of Minnesota, Minneapolis, MN, USA; 66 Department of Neuroscience, Western University, London, ON, Canada; 67 Departments of Psychology and Psychiatry, Neuroscience Program, Western University, London, ON, Canada; 68 Department of Psychiatry, Baylor College of Medicine, Houston, TX, USA; 69 Department of Psychiatry, Yale University School of Medicine, New Haven, CT, USA; 70 Division of Clinical Neuroscience, National Center for PTSD, West Haven, CT, USA; 71 Departments of Psychiatry and of Psychology, Wu Tsai Institute, Yale University, New Haven, CT, USA; 72 Departments of Comparative Medicine, Neuroscience and Psychology, Wu Tsai Institute, Yale University, New Haven, CT, USA; 73 Ministry of Defence, Brain Research and Innovation Centre, Utrecht, The Netherlands; 74 Brain Research and Innovation Centre, University Medical Center, Utrecht, The Netherlands; 75 Department of Neurology, University of Utah School of Medicine, Salt Lake City, UT, USA; 76 George E. Wahlen Veterans Affairs Medical Center, Salt Lake City, UT, USA; 77 Imaging Genetics Center, Mark and Mary Stevens Neuroimaging & Informatics Institute, Keck School of Medicine, University of Southern California, Marina del Rey, CA, USA; 78 Department of Radiology, Stanford University, Stanford, CA, USA; 79 Department of Physical Medicine and Rehabilitation, Virginia Commonwealth University, Richmond, VA, USA; 80 Richmond VAMC, Central Virginia VA Health Care System, Richmond, VA, USA; 81 H. Ben Taub Department of Physical Medicine and Rehabilitation, Baylor College of Medicine, Houston, TX, USA; 82 Department of Psychiatry, Leiden University Medical Center, Leiden, The Netherlands; 83 Leiden Institute for Brain and Cognition, Leiden University, Leiden, The Netherlands; 84 Department of Psychology, Harvard University, Cambridge, MA, USA; 85 Department of Radiology, University of Washington, Seattle, WA, USA; 86 Department of Psychology, University of Washington, Seattle, WA, USA; 87 Institut d’Investigacions Biomèdiques August Pi i Sunyer (IDIBAPS), CIBERSAM, University of Barcelona, Barcelona, Spain; 88 Department of Psychiatry, University of Oxford, Oxford, UK; 89 Highfield Unit, Warneford Hospital, Oxford, UK; 90 Centre for Youth Mental Health, University of Melbourne, Parkville, VIC, Australia; 91 Department of Psychology, Institute of Psychiatry, Psychology & Neuroscience, King’s College London, London, UK; 92 Department of Psychiatry, KU Leuven, Leuven, Belgium

**Keywords:** brain structure, gray matter volume, neuroimaging, PTSD, trauma, voxel-based morphometry

## Abstract

**Background:**

Patients with posttraumatic stress disorder (PTSD) exhibit smaller regional brain volumes in commonly reported regions including the amygdala and hippocampus, regions associated with fear and memory processing. In the current study, we have conducted a voxel-based morphometry (VBM) meta-analysis using whole-brain statistical maps with neuroimaging data from the ENIGMA-PGC PTSD working group.

**Methods:**

T1-weighted structural neuroimaging scans from 36 cohorts (PTSD *n* = 1309; controls *n* = 2198) were processed using a standardized VBM pipeline (ENIGMA-VBM tool). We meta-analyzed the resulting statistical maps for voxel-wise differences in gray matter (GM) and white matter (WM) volumes between PTSD patients and controls, performed subgroup analyses considering the trauma exposure of the controls, and examined associations between regional brain volumes and clinical variables including PTSD (CAPS-4/5, PCL-5) and depression severity (BDI-II, PHQ-9).

**Results:**

PTSD patients exhibited smaller GM volumes across the frontal and temporal lobes, and cerebellum, with the most significant effect in the left cerebellum (Hedges’ *g* = 0.22, *p_corrected_* = .001), and smaller cerebellar WM volume (peak Hedges’ *g* = 0.14, *p_corrected_* = .008). We observed similar regional differences when comparing patients to trauma-exposed controls, suggesting these structural abnormalities may be specific to PTSD. Regression analyses revealed PTSD severity was negatively associated with GM volumes within the cerebellum (*p*_*corrected*_ = .003), while depression severity was negatively associated with GM volumes within the cerebellum and superior frontal gyrus in patients (*p*_*corrected*_ = .001).

**Conclusions:**

PTSD patients exhibited widespread, regional differences in brain volumes where greater regional deficits appeared to reflect more severe symptoms. Our findings add to the growing literature implicating the cerebellum in PTSD psychopathology.

## Introduction

Most individuals experience trauma at some time in their lives, where 70% of respondents in the World Mental Health Survey reported exposure to at least one traumatic event [1]. The lifetime prevalence of posttraumatic stress disorder (PTSD) is estimated to be 10% in the United States [2], where symptoms are characterized as re-experiencing, hyperarousal, avoidance of trauma-related situations, negative cognition, and emotional numbing, which can last for years after the event [3].

Brain structural abnormalities have been consistently associated with PTSD, with recent structural neuroimaging meta-analyses reporting smaller gray matter (GM) volumes within the frontal lobe, hippocampus, anterior cingulate cortex (ACC), and insula in patients with PTSD when compared to controls [4–9]. The PTSD working group from the Enhancing Neuroimaging Genetics through Meta-Analysis (ENIGMA)-Psychiatric Genomics Consortium (PGC) (https://enigma.ini.usc.edu) has previously analyzed structural brain differences between patients with PTSD and controls by pooling data provided by research groups around the world, using segmented brain volumes derived by FreeSurfer (https://surfer.nmr.mgh.harvard.edu). In a region-of-interest (ROI) approach, Logue et al. [10] analyzed eight a priori subcortical structures comparing 794 patients with PTSD and 1074 controls and found that patients with PTSD had significantly smaller hippocampal volumes compared to trauma-exposed (TE) controls. Wang et al. [11] conducted a mega-analysis across 68 cortical regions comparing 1379 patients with PTSD and 2192 controls and revealed that patients with PTSD exhibited significantly smaller GM volumes across the orbitofrontal region, superior temporal gyrus, insula, lingual, and superior parietal gyri, and that these regions were also negatively correlated with PTSD symptom severity. However, Wang et al. used a control group comprising both TE- and non-trauma-exposed (nTE) controls, which was noted as a limitation in their study. Both Logue et al. [10] and Wang et al. [11] did adjust for sex, age, total intracranial volume (ICV), and scanner site. More recently, ENIGMA-PTSD has published two studies: the first examined only the cerebellum and found significantly smaller GM and white matter (WM) cerebellar volumes and cerebellar subregions in patients with PTSD compared with controls [12], and the second reported diminished cortical thickness associated with PTSD within the prefrontal cortex, insula, occipital cortex, and cingulate cortex [13].

To complement the existing research, the current study used a whole-brain voxel-based morphometry (VBM) approach to meta-analysis. VBM methodologies are unconstrained by anatomical boundaries and can observe differential effects at a voxel level, while effects in ROI analyses are only observed at the level of the predefined region. VBM analyses also encompass the whole brain and include WM structures at the voxel level. VBM meta-analyses typically involve pooling published peak coordinates, which represent the voxel location where the statistical effect is strongest. This results in a loss of valuable information as nonsignificant data are excluded. An alternative approach, used in the current study, is to use whole-brain statistical maps that are produced at the end of the VBM processing pipeline. Statistical maps contain the statistical results for a given analysis (e.g., *t*-values from group comparisons) at the voxel level across the whole brain, meaning data from all voxels are included in the analysis rather than just peak values. This methodology has previously been used to study PTSD by Bromis et al. [4], where the authors combined statistical maps and peak coordinates. This has demonstrated more accurate results in comparison to using peak coordinates [14]. However, there are practical challenges in that statistical maps are not always made available by authors, and if they are, different VBM processing parameters can affect results [15].

To address these issues, we have developed the ENIGMA-VBM tool [16]. The tool is designed for contributing sites to process their data locally using a standardized VBM pipeline with automated quality control checks. Sites share the resulting statistical maps, containing group-level data, with the researchers conducting the meta-analysis, thus addressing participant-level data privacy concerns. In the current study, we have used the ENIGMA-VBM tool to conduct the largest VBM meta-analysis in PTSD to date using only whole-brain statistical maps.

Our main analysis compared total and regional GM and WM volumes between patients with PTSD and controls, where we expected that patients would exhibit smaller regional volumes within the frontal lobe, hippocampus, ACC, insula, cerebellum, and total GM volumes compared with controls, consistent with previous literature [4–12]. In exploratory analyses, we conducted subgroup investigations to compare patients with PTSD with TE controls to try to disentangle the effects of trauma exposure from PTSD-related structural brain abnormalities. We also compared controls with and without trauma exposure to test the effects of trauma per se [4]. As the ENIGMA-PTSD sample consisted of participants from military and civilian backgrounds, we analyzed military- and civilian-recruited cohorts separately. This exploratory analysis aimed to examine whether underlying sample characteristics may be associated with different brain regions, as military populations experience more combat-related trauma [17, 18] and exhibit poorer treatment outcomes [19]. Previous evidence suggests that combat trauma is related to more severe PTSD symptoms [17] and has a higher risk of lifetime PTSD with poorer psychosocial outcomes [20]. This may be due to the extended duration of military traumatic experiences as compared with more acute civilian trauma, such as motor vehicle accidents [21]. We also examined associations between regional brain volumes in patients with PTSD and clinical variables such as PTSD severity, depression severity, and childhood trauma. In sensitivity analyses, we adjusted for sex due to higher incidence rates of PTSD in females [22, 23] and sex differences in traumatic experiences [24]. Finally, we performed several sensitivity analyses to assess the robustness of our findings by varying VBM processing parameters.

## Methods

### Cohorts and participants

Structural neuroimaging scans and clinical data were provided by the ENIGMA-PGC PTSD working group for 36 cohorts from 28 sites, comprising 1309 patients with PTSD and 2198 controls. Controls were both TE and nTE (Table 1). One site comprised only TE and nTE controls. Most cohorts were adult samples, except for two non-adult cohorts consisting of participants under the age of 20. Cohorts consisted of military- and civilian-recruited samples, and one sample of police officers (Table 2). Patients were diagnosed according to the Diagnostic and Statistical Manual of Mental Disorders (DSM)-IV or DSM-5 criteria using the instruments listed in Table 2. Sites had obtained approval from their local ethics committee and written informed consent from study participants. Further study details and inclusion and exclusion criteria can be found in Supplementary Tables S1 and S2 in Supplement A.

**Table 1.** Clinical and demographic characteristics for each cohort

Cohort	Sample, *N*				Female, *N (%)*				Age, *mean (SD)*			
	PTSD	Controls	TE controls^a^	nTE controls^a^	PTSD	Controls	TE controls	nTE controls	PTSD	Controls	TE controls	nTE controls
ADNIDOD 1	50	61	60	0	0 (0.0)	1 (1.6)	1 (1.6)	NA	67.8 (3.9)	69.3 (4.8)	69.5 (4.7)	NA
ADNIDOD 2	17	31	31	0	0 (0.0)	0 (0.0)	0 (0.0)	NA	67.9 (3.2)	69.7 (4.7)	69.7 (4.7)	NA
AMC	37	37	37	0	16 (43.2)	18 (48.6)	0 (0.0)	NA	40.2 (10.0)	39.6 (10.1)	39.6 (10.1)	NA
Beijing	42	46	46	0	29 (69.0)	24 (52.2)	24 (52.2)	NA	53.7 (7.9)	43.3 (9.7)	43.3 (9.7)	NA
Columbia–3	53	36	36	0	34 (64.2)	24 (66.7)	24 (66.7)	NA	36.4 (9.3)	35.0 (10.6)	35.0 (10.6)	NA
Columbia–6	25	55	32	23	7 (28.0)	28 (50.9)	15 (46.9)	13 (56.5)	37.3 (13.6)	35.2 (12.1)	36.2 (12.3)	33.7 (12.0)
Duke 1	11	73	72	0	2 (18.2)	16 (21.9)	16 (22.2)	NA	37.1 (9.1)	39.6 (9.4)	39.5 (9.5)	NA
Duke 2	15	33	33	0	4 (26.7)	5 (15.2)	5 (15.2)	NA	42.2 (11.4)	41.1 (9.3)	41.1 (9.3)	NA
Duke 3	15	31	31	0	2 (13.3)	8 (25.8)	8 (25.8)	NA	41.8 (9.2)	37.4 (11.4)	37.4 (11.4)	NA
Duke 4	36	75	69	0	5 (13.9)	14 (18.7)	14 (20.3)	NA	38.2 (9.6)	37.5 (10.3)	37.3 (10.3)	NA
Emory	14	48	48	0	14 (100.0)	48 (100.0	48 (100.0)	NA	42.1 (13.3)	40.0 (11.8)	40.0 (11.8)	NA
INTRuST 1	72	147	118	26	16 (22.2)	71 (48.3)	57 (48.3)	13 (50.0)	37.0 (9.4)	32.0 (12.2)	33.2 (12.5)	26.2 (9.4)
INTRuST 2	31	94	80	10	8 (25.8)	41 (43.6)	33 (41.3)	4 (40.0)	44.6 (11.4)	37.3 (12.9)	36.8 (13.0)	41.4 (11.6)
Leiden	21	30	NA	NA	18 (85.7)	26 (86.7)	NA	NA	15.9 (1.9)	14.7 (1.6)	NA	NA
LIMBIC-CENC 1	84	179	NA	NA	18 (21.4)	30 (16.8)	NA	NA	44.8 (8.7)	44.6 (9.8)	NA	NA
LIMBIC-CENC 2	76	84	NA	NA	7 (9.2)	8 (9.5)	NA	NA	34.7 (6.9)	33.2 (7.4)	NA	NA
LIMBIC-CENC 3	81	144	NA	NA	9 (11.1)	19 (13.2)	NA	NA	39.8 (8.4)	39.1 (9.3)	NA	NA
McLean 1	50	26	20	5	50 (100.0)	26 (100.0)	20 (100.0)	5 (100.0)	35.1 (13.4)	33.5 (11.3)	34.0 (11.3)	30.4 (13.0)
McLean 2	22	74	35	39	13 (59.1)	39 (52.7)	19 (54.3)	20 (51.3)	35.6 (7.6)	33.7 (9.1)	33.7 (9.1)	33.7 (9.3)
Minnesota	12	50	50	0	2 (16.7)	3 (6.0)	3 (6.0)	NA	38.6 (8.2)	43.9 (9.6)	43.9 (9.6)	NA
Münster	21	26	NA	NA	21 (100.0)	21 (80.8)	NA	NA	27.4 (7.0)	26.5 (7.4)	NA	NA
South Dakota	78	44	28	8	17 (21.8)	7 (15.9)	1 (3.6)	2 (25.0)	28.8 (7.1)	29.9 (6.9)	32.0 (6.2)	31.0 (6.2)
Stanford	30	50	45	5	6 (20.0)	17 (34.0)	14 (31.1)	3 (60.0)	31.4 (10.1)	32.6 (11. 8)	32.7 (11.6)	31.6 (14.6)
Toledo	15	63	63	0	7 (46.7)	29 (46.0)	29 (46.0)	NA	40.9 (9.5)	34.3 (11.5)	34.3 (11.5)	NA
UCT^b^	NA	68	18	50	NA	68 (100.0)	18 (100.0)	50 (100.0)	NA	26.7 (6.4)	27.2 (5.9)	26.5 (6.6)
UMC BETTER	55	52	NA	NA	1 (1.8)	0 (0.0)	NA	NA	36.1 (9.8)	36.0 (10.2)	NA	NA
VA Minn DEFEND	27	82	82	0	1 (3.7)	3 (3.7)	3 (3.7)	NA	32.0 (5.2)	32.5 (7.9)	32.5 (7.9)	NA
VA Minn SATURN	55	62	62	0	0 (0.0)	10 (16.1)	10 (16.1)	NA	30.9 (7.8)	34.3 (8.8)	34.3 (8.8)	NA
VA Waco	59	31	31	0	6 (10.2)	2 (6.5)	2 (6.5)	NA	39.4 (9.7)	42.5 (11.8)	42.5 (11.8)	NA
VA West Haven	35	30	30	0	4 (11.4)	3 (10.0)	3 (10.0)	NA	35.2 (9.3)	34.3 (10.2)	34.3 (10.2)	NA
Vanderbilt	15	35	20	15	1 (6.7)	8 (22.9)	5 (25.0)	3 (20.0)	33.9 (4.7)	30.2 (4.2)	31.6 (3.9)	28.5 (4.0)
Washington	33	116	60	56	15 (45.5)	59 (50.9)	34 (56.7)	25 (44.6)	12.7 (2.7)	12.9 (2.7)	13.2 (2.7)	12.6 (2.6)
Western Ontario	59	39	2	36	44 (74.6)	25 (64.1)	0 (0.0)	24 (66.7)	38.5 (12.7)	33.5 (12.2)	35.0 (2.8)	33.74 (12.6)
Wisconsin-Madison	19	38	38	0	3 (15.8)	1 (2.6)	1 (3.6)	NA	30.4 (6.2)	30.8 (6.7)	30.8 (6.7)	NA
Wisconsin-Milwaukee	22	60	60	0	11 (50.0)	30 (50.0)	30 (50.0)	NA	28.7 (8.2)	34.4 (10.9)	34.4 (10.9)	NA
Yale	22	48	25	23	3 (13.6)	8 (16.7)	1 (4.0)	7 (30.4)	31.8 (6.9)	29.4 (8.2)	32.9 (8.5)	25.7 (6.0)
Total	1309	2198	1362	296	394 (30.1)	740 (33.7)	438 (32.2)	169 (57.1)	37.7 (13.2)	35.9 (13.8)	37.2 (14.3)	27.0 (11.5)

Abbreviations: nTE, non-trauma-exposed; PTSD, posttraumatic stress disorder; TE, trauma-exposed.

*Note:* For sites with multiple scanners, participants were grouped by a scanner model where possible to form processing cohorts.

a Where the control subgroups do not add-up to the total number of controls, which is due to unspecified trauma exposure of the control participant.

b UCT did not have enough current patients with PTSD (<8) for the main analysis and was only included in the subgroup comparison between TE and nTE controls.

**Table 2.** Sample type and patient with PTSD symptom severity for each cohort

Cohort		PTSD	
	Sample type^a^	Diagnostic instrument	Patient PTSD severity^b^, mean % (SD)
ADNIDOD 1	Military	CAPS–4	43.0 (10.7)
ADNIDOD 2	Military	CAPS–4	39.4 (7.5)
AMC	Police	CAPS–4	49.9 (10.2)
Beijing	Civilian	PCL–5	53.1 (13.0)
Columbia–3	Civilian	CAPS–4	58.9 (11.4)
Columbia–6	Civilian	CAPS–5	45.7 (11.6)
Duke 1	Military	CAPS–4, CAPS–5	40.5 (18.3)
Duke 2	Military	CAPS–4, CAPS–5	49.5 (18.2)
Duke 3	Military	CAPS–4	47.2 (12.4)
Duke 4	Military	CAPS–4	54.3 (14.6)
Emory	Civilian	CAPS–4	44.5 (10.1)
INTRuST 1	Military, Civilian	PCL-C	63.5 (19.5)
INTRuST 2	Military, Civilian	PCL-C	62.0 (16.9)
Leiden	Civilian	ADIS-C/P	40.7 (22.0)
LIMBIC-CENC 1	Military	PCL–5	61.6 (12.8)
LIMBIC-CENC 2	Military	PCL–5	64.0 (13.9)
LIMBIC-CENC 3	Military	PCL–5	58.1 (12.7)
McLean 1	Civilian	CAPS–5	64.2 (14.2)
McLean 2	Civilian	CAPS–4	43.6 (13.5)
Minnesota	Military	CAPS–4	39.3 (8.2)
Münster	Civilian	SCID–4	46.0 (20.2)
South Dakota	Military, Civilian	PCL-M, PCL-C	55.6 (15.4)
Stanford	Civilian	CAPS–4	43.8 (13.9)
Toledo	Military, Civilian	CAPS–4	47.0 (11.6)
UCT^c^	Civilian	MINI	NA
UMC BETTER	Military, Civilian	CAPS–4	52.0 (9.7)
VA Minn DEFEND	Military	CAPS–4	47.9 (17.7)
VA Minn SATURN	Military	CAPS–4	46.1 (13.1)
VA Waco	Military	PCL–5	70.0 (14.5)
VA West Haven	Military	CAPS–4	49.9 (11.4)
Vanderbilt	Military	CAPS–5	33.7 (5.7)
Washington	Civilian	CAPS–5	18.0 (4.3)
Western Ontario	Civilian	CAPS–4, CAPS–5	51.4 (10.4)
Wisconsin-Madison	Military	CAPS–4	47.8 (10.9)
Wisconsin-Milwaukee	Civilian	CAPS–5	35.8 (9.2)
Yale	Military	CAPS–4	36.8 (17.7)
Total			52.6 (17.0)

Abbreviations: nTE, non-trauma-exposed; PTSD, posttraumatic stress disorder; TE, trauma-exposed.

*Note*: PTSD diagnosis and severity scales: CAPS-4/5 = Clinician-Administered PTSD Scale for DSM-IV/DSM-5 [61, 62]; PCL-5/C/M = PTSD Checklist for DSM-5 (Civilian or Military version) [63]; ADIS-C = Anxiety Disorders Interview Schedule for Children [64]; SCID = Structured Clinical Interview for DSM [65]; MINI = Mini International Neuropsychiatric Interview [66]; MPSS = Modified PTSD Symptom Scale [67]; TSCC = Trauma Symptom Checklist for Children [68]; PDS = Posttraumatic Stress Diagnostic Scale [69].

a PTSD patients and controls were recruited from the same sample types.

b PTSD severity has been quantified as a percentage of the total score for visual comparison across cohorts. Raw scores are available in Supplement A (Supplementary Table S4).

### Cohort-level image processing and analysis

The ENIGMA-VBM tool (https://sites.google.com/view/enigmavbm) was developed for the ENIGMA consortium by the authors for VBM case-control studies [16]. The tool processes T1-weighted brain images for each cohort using the DARTEL (Diffeomorphic Anatomical Registration Through Exponentiated Lie Algebra) [25] VBM processing pipeline in SPM12 (Statistical Parametric Mapping; https://www.fil.ion.ucl.ac.uk/spm/) within MATLAB, using a smoothing kernel of 8 mm and Jacobian modulated data, controlling for age and total ICV. A detailed description of the tool is available in Supplement B.

Sites provided T1-weighted brain imaging and clinical data for participants. Scanner information and acquisition methods can be found in Supplementary Table S3. Each cohort was processed using the ENIGMA-VBM tool v1.076, which conducted GM and WM voxel-wise statistical analysis comparing patients with controls. For sites with multicenter data or multiple studies, we used cohorts for VBM processing where participants were grouped based on scanner model, where possible, to minimize the effects of scanner model [26, 27], while ensuring there were sufficient patients with PTSD and controls for analysis. As an example, the cohorts ADNIDOD 1 and ADNIDOD 2 are from the same study but have been processed as two cohorts to account for different scanner models.

#### Group comparisons of regional brain volumes

The main analysis compared voxel-wise GM and WM volumes between patients with PTSD and all controls (inclusive of TE and nTE controls). Exploratory subgroup analyses compared: (1) patients with PTSD to TE controls; (2) TE to nTE controls; (3) patients with PTSD to all controls from military-recruited cohorts; and (4) patients with PTSD to all controls from civilian-recruited cohorts. Sample sizes for each analysis varied depending on data availability, such as the trauma exposure, of the controls. All group comparisons were adjusted for age and total ICV, as these variables account for the most variance in segmented GM and WM data.

#### Associations between regional brain volumes and clinical variables

The ENIGMA-VBM tool also conducted regression analyses to examine the association between regional brain volumes and clinical variables within the patient group. The regression analyses were performed within each cohort prior to being pooled for meta-analysis. This approach has greater statistical power than meta-regression, which uses a mean value of the clinical variable for each cohort.

We performed exploratory regression analyses to examine the associations between regional brain volumes and the following clinical covariates: PTSD severity, depression severity, childhood trauma, alcohol use disorder, drug use disorder, and antidepressant medication use. Alcohol use disorder, drug use disorder, and antidepressant medication were coded as dichotomous variables. PTSD severity, depression severity, and childhood trauma were analyzed using the participant’s total score for each variable. Further details regarding the treatment of the clinical variables are reported in Supplement A. All regression analyses were adjusted for age, ICV, and sex. Sex was included to adjust for potential associations with the clinical variables, as it is well-established that females are more likely to develop PTSD as compared to males [28, 29], and sex has been associated with PTSD comorbidities, including depression, alcohol use disorder, and drug use disorder [30–32].

#### Sensitivity analysis

The tool performed several sensitivity analyses to test the robustness of our findings against changes in VBM processing parameters including: (1) different smoothing kernels of 2, 4, and 12 mm; (2) different combinations of covariates of no interest (e.g., age and sex, or no covariates); (3) proportional scaling of voxels, where each voxel is scaled by the fraction of total ICV; and (4) using nonmodulated data.

For each analysis, the resulting statistical map contained the results for approximately 200,000 voxels, reflecting volumetric group differences or regression coefficients at each voxel.

### Meta-analysis across cohorts

The statistical maps were pooled across cohorts for meta-analysis using the software Seed-based *d*-Mapping with Permutation of Subject Images (SDM-PSI v6.22; https://www.sdmproject.com) [33]. In summary, the SDM-PSI process involves the following main steps: (1) statistical maps are converted to effect size maps using standard formulae; (2) the mean of the voxel values is calculated via random effects meta-analysis; and (3) a subject-based permutation test is conducted to family-wise error (FWE) correct for multiple comparisons using threshold-free cluster enhancement (TFCE) with statistical thresholding (*p* < .025, voxel extent ≥ 10).

Total GM and WM volumes were compared between patients with PTSD and all controls using the unadjusted mean and standard deviation (SD) statistics at a cohort level as reported by the ENIGMA-VBM tool. The statistics from each cohort were pooled using an inverse-variance weighted random-effects model in STATA (release 17).

In sensitivity analyses, we repeated the meta-analysis of the main group comparison to exclude two non-adult cohorts, consisting of participants under the age of 20, to test for changes to our results. A further eight cohorts included participants who had been diagnosed with moderate-to-severe traumatic brain injury (TBI), and we similarly repeated the main group comparison, excluding the affected participants (PTSD *n*_TBI_ = 382, controls *n*_TBI_ = 527) from our meta-analysis. We used a parcel-based correlation analysis [34, 35] in R (version 4.3.1) to calculate Pearson’s correlation coefficient to compare the spatial pattern of regional GM and WM differences between a given sensitivity analysis and our main group findings. Using a parcel-based approach mitigates the issue in voxel-based correlations where adjacent voxels are not independent. Further details are reported in Supplement A.

## Results

All effect size and statistical maps are available online (https://neurovault.org/collections/QOAYXFZK/). The *p* values reported below are FWE-corrected for multiple comparisons using TFCE for the VBM analyses. The main findings are reported below, with full results and figures reported in Supplement A. Cohort sample characteristics are reported in Tables 1 and 2, and descriptive statistics for the clinical variables are reported in Supplementary Tables S4 and S5.

### Group comparisons of regional brain volumes

#### PTSD versus controls

**Figure 1.**
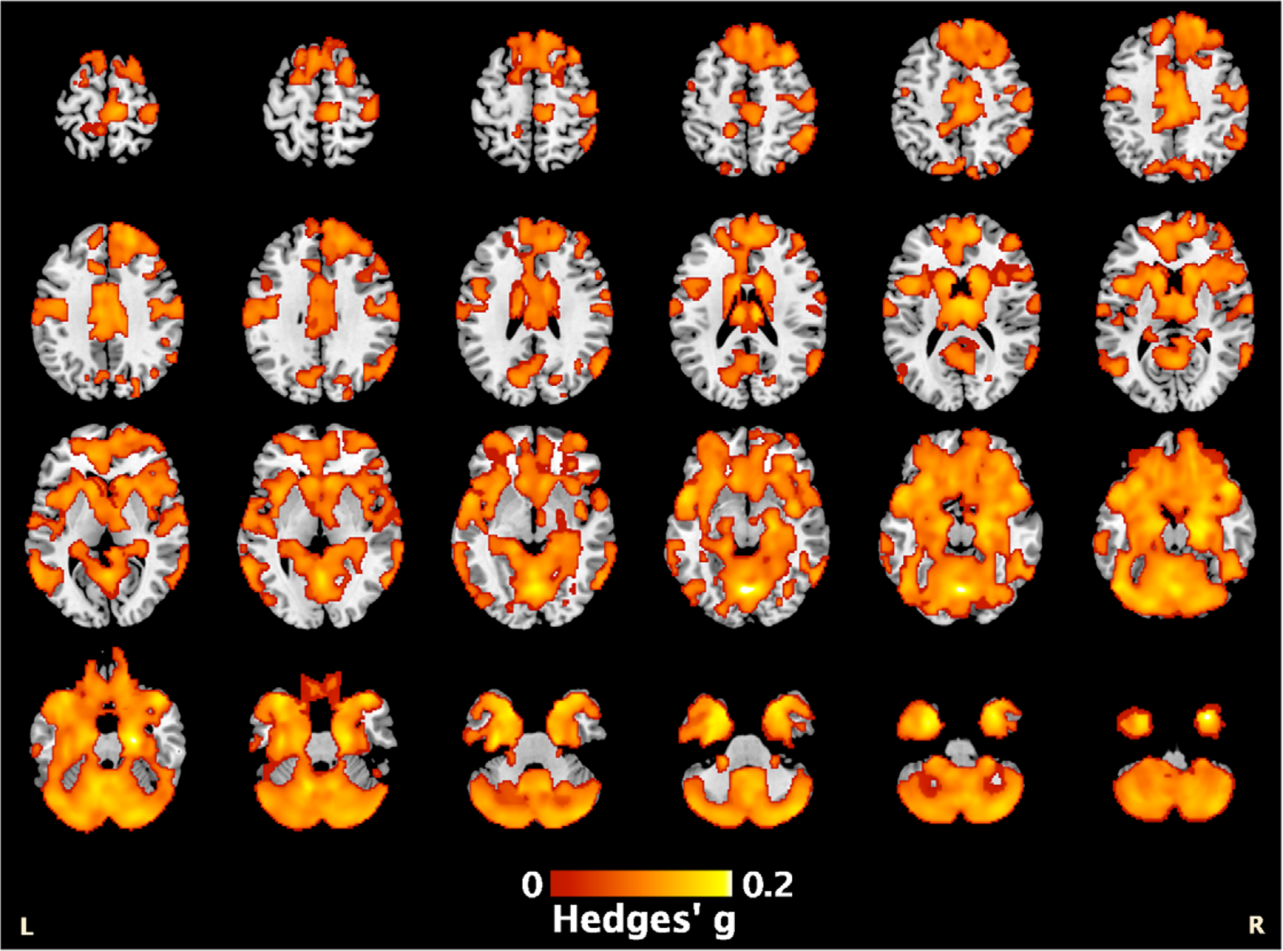
Patients with PTSD exhibited lower regional gray matter volume compared to controls throughout the brain as seen in the orange highlighted regions in the figure, with a peak effect in the left cerebellum [−4,−72,-10] (see also Supplementary Table S6).

The main group comparison analyzed data from 35 cohorts comprising 1309 patients with PTSD and 2130 controls, inclusive of TE and nTE controls. Patients exhibited smaller GM volumes in a large cluster extending across the brain, encompassing the frontal and temporal lobes, thalamus, and cerebellum (Figure 1; Supplementary Table S6). Peak effects were observed in the left cerebellum (Hedges’ *g* = 0.22, *p_TFCE_* = .001, MNI [−4,−72,−10]) and right parahippocampus (Hedges’ *g* = 0.20, *p_TFCE_* = .001, MNI [22,−18,−24]). Patients exhibited smaller WM volumes in a single cluster within the cerebellum, with peak effects in the middle cerebellar peduncles (Hedges’ *g* = 0.14, *p_TFCE_* = .008, MNI [−16,−54,−38]) and left cerebellum (Hedges’ *g* = 0.14, *p_TFCE_* = .009, MNI [−6,−54,−18]) (Supplementary Table S6; Supplementary Figure S1). There were no regions where brain volumes were greater in patients than in controls.

Patients with PTSD exhibited significantly lower *total* GM volume (Hedges’ *g* = −0.18, 95% CI [−0.29,−0.08], *p* = .001) (Supplementary Figure S2). There was no significant difference in *total* WM volume between groups (Supplementary Figure S3).

#### Subgroup analyses

In comparing 912 patients with PTSD to 1342 TE controls, patients exhibited smaller GM volumes in a similar spatial pattern to the main finding, and greater WM volumes within the corpus callosum (Supplementary Table S7; Supplementary Figure S4). When comparing 416 TE and 250 nTE controls, there were no significant GM or WM differences between groups.

In our analysis comparing patients with PTSD and controls from 19 military-recruited cohorts, the results were similar to the main findings, with patients exhibiting smaller GM volumes in a cluster across the frontal and temporal lobes, and cerebellum, and smaller WM volumes adjacent to the striatum (Supplementary Table S8; Supplementary Figure S5). In a separate analysis of 13 civilian-recruited cohorts, patients exhibited less widespread effects, with smaller GM volumes in the parahippocampus and cerebellum, and greater WM volumes within the corpus callosum (Supplementary Table S9; Supplementary Figure S6).

### Associations between regional brain volumes and clinical variables in patients with PTSD

**Figure 2.**
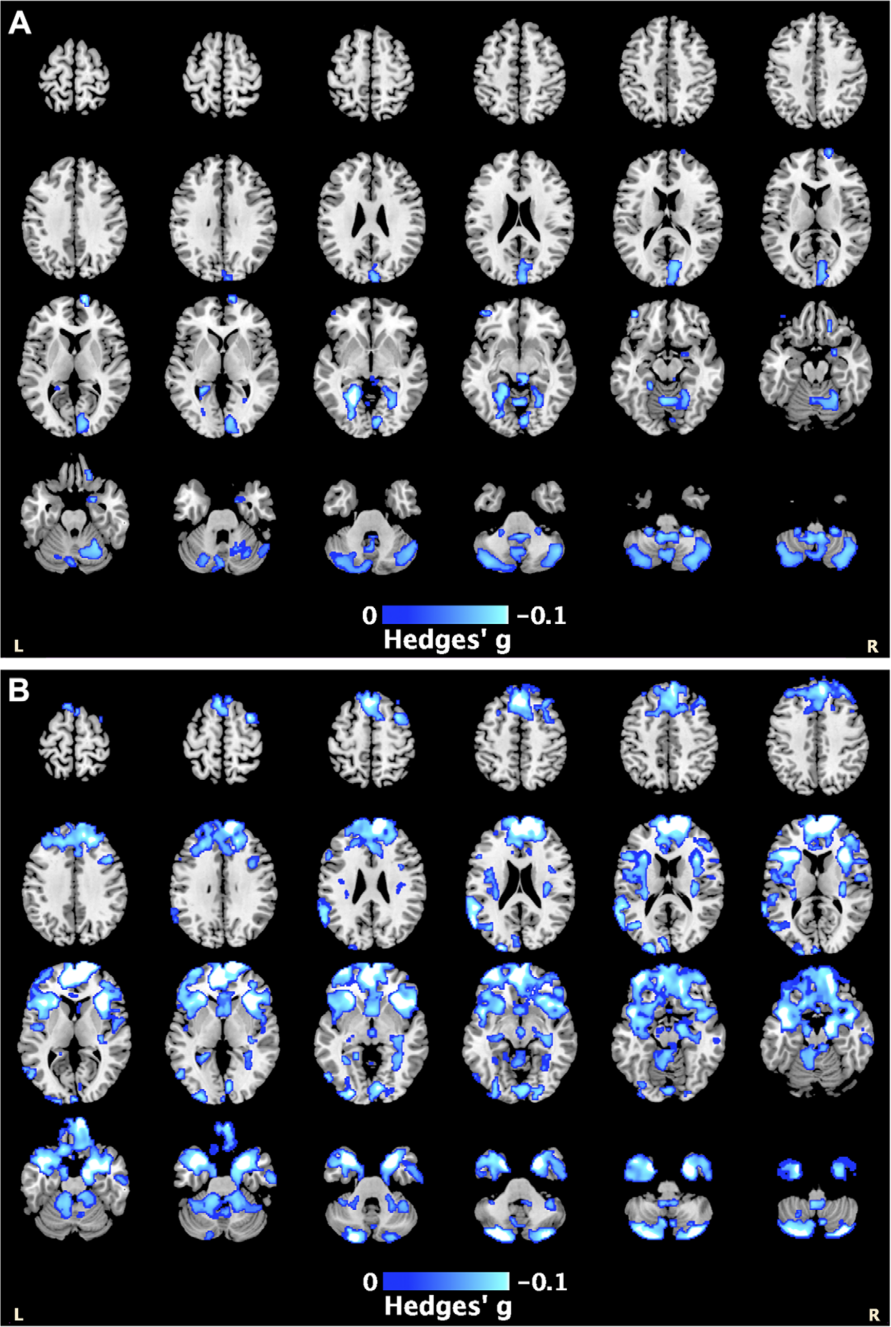
The blue highlighted regions represent smaller gray matter volumes associated with: (A) higher PTSD severity scores, with the peak effect in the right cerebellum [4,−48,−58]; and (B) higher depression severity scores, with the peak effect in the right superior frontal gyrus [14, 66, 6] (see also Supplementary Tables S12 and S13).

PTSD severity data were available for 35 cohorts (PTSD *n* = 1283). A higher PTSD severity score was associated with smaller GM volumes within the cerebellum, lingual gyrus, and superior frontal gyrus, with a peak effect in the right cerebellum (Hedges’ *g* = −0.11, *p_TFCE_* = .003, MNI [4,−48,−58]) (Figure 2A; Supplementary Table S10).

Depression severity data were available for 30 cohorts (PTSD *n* = 1023). Higher depression severity was associated with lower GM volumes within the frontal, temporal, and cerebellar regions, with a peak effect in the right superior frontal gyrus (Hedges’ *g* = −0.15, *p_TFCE_* = .001, MNI [14, 66, 6]) (Figure 2B; Supplementary Table S11).

680 patients with PTSD had available data on alcohol use disorder status, where 25.6% were identified as having an alcohol use disorder. Alcohol use disorder was associated with lower GM volumes within the cerebellum and temporal lobe, with a peak effect in the left fusiform gyrus (Hedges’ *g* = −0.15, *p_TFCE_* = .001, MNI [−34,−56,−6]) (Supplementary Table S12; Supplementary Figure S7).

364 patients with PTSD had available data on antidepressant medication, where 30.8% were identified as using antidepressant medication. We observed smaller GM volumes associated with antidepressant medication use in a small cluster within the left temporal gyrus, with a peak effect in the left inferior temporal gyrus (Hedges’ *g* = −0.17, *p_TFCE_* = .017, MNI [−60,−26,−18]) (Supplementary Table S13; Supplementary Figure S8).

There were no significant associations observed between GM volumes and childhood trauma (PTSD *n* = 507) or drug use disorder (PTSD *n* = 405). There were also no significant associations found between WM volumes and any of the clinical variables.

### Sensitivity analysis

The spatial pattern of effect sizes was similar to that of the main findings for GM and WM when we excluded two nonadult cohorts from the analysis (Pearson’s *r* > 0.9) (Supplementary Table S14; Supplementary Figure S9). When we excluded participants with moderate-to-severe TBI, the spatial pattern of effect sizes was also similar to the main findings for GM and WM (Pearson’s *r* > 0.9) (Supplementary Table S15; Supplementary Figure S10). However, different WM clusters passed the significance threshold, where patients with PTSD exhibited significantly *greater* WM volumes within the corpus callosum. Patients still exhibited smaller WM volumes in the cerebellum as in the main findings, but these effects were no longer significant.

The results from the sensitivity analyses using different VBM parameters are reported in Supplement A (Supplementary Tables S16–S27; Supplementary Figures S11–S22). The correlation coefficients comparing the effect size maps from the sensitivity analyses to that of the main group comparison are reported in Supplementary Table S28. Using nonmodulated data effected the biggest change to our results (Pearson’s *r >* 0.49), while controlling for different covariates had a lesser effect on our results (Pearson’s *r* > 0.76). Using different smoothing kernels were in good agreement with our main result (Pearson’s *r* > 0.94).

### Heterogeneity of the effect size

The extent of heterogeneity of the effect size was relatively low across the analyses. The main group comparison had a mean I^2^ of 8.15% across all GM voxels and of 4.67% across all WM voxels (Supplementary Figure S23). Heterogeneity is reported for each analysis in the tables within Supplement A, expressed as the mean I^2^ across all GM or WM voxels, and at the peak coordinates.

## Discussion

Patients with PTSD exhibited smaller total GM volume than controls and in regions widespread across the brain with a peak effect in the cerebellum. Patients with PTSD had lower WM volumes within the cerebellum but exhibited no differences in total WM volume. We observed similar findings in comparing patients with PTSD to TE controls, but there were no differences between TE and nTE controls. Military-recruited cohorts exhibited group differences in similar GM regions as the main findings, while GM differences appeared to be less widespread in civilian-recruited cohorts. Regional GM volumes were negatively associated with PTSD severity, depression severity, alcohol use disorder, and antidepressant medication within patients with PTSD.

### Regional and total brain volumes

Our findings are largely consistent with existing meta-analyses that found smaller total GM volumes in patients with PTSD compared to controls [4–9], and with previous ENIGMA-PTSD FreeSurfer studies [10, 11], with effects in similar regions including the frontal lobe, cingulate cortex, hippocampus, and amygdala. However, comparisons with ROI studies are provided cautiously given the different methodologies of the present study relative to published studies. Our analysis revealed similar regional volume differences when we compared patients to TE controls, suggesting that these regions could be related to PTSD itself, rather than being associated with trauma exposure. This is further supported where we found no significant differences between TE and nTE controls. However, the smaller sample of nTE controls may have been underpowered to detect subtle differences between the control subgroups.

We observed lower GM and WM volumes within the cerebellum in patients, a finding not reported in previous VBM meta-analyses [4–9]. From previous work, Serra-Blasco et al. [8] reported significantly lower GM volumes in the cerebellum in patients with PTSD when compared to those with anxiety disorders, suggesting that this regional finding could be specific to PTSD. In ROI studies, the cerebellum is rarely included as it has been historically associated with motor control [36]. The disparities between the current study and previous meta-analyses may be due to the increased power and homogeneity within the VBM processing in the current study from using the ENIGMA-VBM tool, or from differences in the sample characteristics. Notably, prior meta-analyses included 50–80% of samples from Europe and Asia [4–9], while fewer than 15% of cohorts in the current study were from these regions. Our findings are consistent with individual neuroimaging studies that have reported smaller cerebellar volumes in patients with PTSD compared with controls [37–39] and further complement the cerebellar mega-analysis by ENIGMA-PTSD, which used a novel parcellation protocol to reveal smaller brain volumes within the cerebellum and its substructures associated with PTSD [12]. Previous functional MRI studies have also found evidence of resting-state dysfunction in the cerebellum in patients with PTSD [40, 41] and cerebellar activation in response to fear [42, 43]. The cerebellum is becoming an increasingly important structure in PTSD [44], with rich connections to regions that are often implicated in stress and trauma such as the hypothalamus, hippocampus, and prefrontal cortex [45].

In examining only military-recruited cohorts, regional GM differences between patients and controls appeared to be more widespread compared to differences observed in civilian-recruited cohorts. This may be driven by characteristics specific to military populations, where previous work has reported lower cortical thickness in veterans with and without PTSD [46] and smaller GM volumes associated with longer military deployment in personnel without PTSD [47]. Our results highlight the importance of considering sample characteristics in future neuroimaging studies and may explain why our findings differ from previous work. For instance, Bromis et al. [4], who similarly meta-analyzed statistical maps, included mostly civilian studies with only 2 military-recruited cohorts, while the current study consisted of 19 military-recruited cohorts.

### GM associations with clinical variables

PTSD severity was negatively associated with GM volumes in posterior regions, including the cerebellum, consistent with individual ROI studies [37–39], and the ENIGMA-PTSD cerebellar mega-analysis [12]. However, our findings contrast with those from a large meta-analysis by Xiao et al. [9] reported associations with the ACC instead. This could be due to methodological differences where the authors used a coordinate-based meta-regression, while in the current study, the regression analysis was conducted within each cohort prior to pooling the resulting statistical maps, which was expected to increase statistical power and sensitivity.

Depression severity was associated with smaller GM volumes in both posterior and frontal regions of the brain. The latter finding may be relevant to functional MRI findings of decreased connectivity within the frontal lobe in PTSD patients with depression [48, 49]. Alcohol use disorder was associated with smaller GM volumes, mainly within the cerebellum, which contrasts with previous work that found associations with the ACC [50]. The negative associations between symptom severity and regional brain volumes indicate that structural abnormalities may exist on a continuum, where patients with more severe symptoms may exhibit greater structural changes within the brain. It is interesting to note that the cerebellum was negatively associated with both depression severity and alcohol use disorder, common comorbidities for PTSD [51, 52]. This suggests that the cerebellum findings are specific to PTSD, with comorbidities potentially affecting further morphological changes. Future work is needed to determine the direction of effect and whether cerebellar abnormalities represent vulnerability factors or consequences of PTSD.

### Sensitivity analyses

We found the significance of the GM results was generally consistent across the sensitivity analyses, while the significance of the WM findings was less robust. The use of nonmodulated data resulted in the biggest difference in results, where findings were only moderately correlated with the main results (*r* = 0.558), with a smaller cluster of significant differences observed in the cerebellum. This may be expected given modulated data has been reported as more sensitive to identifying volumetric differences, while nonmodulated data may be more sensitive to detecting changes in cerebral cortical thickness [53, 54]. We also compared findings using varying smoothing kernel sizes of 2, 4, and 12 mm, where we observed greater spatial extent of significant clusters in regional brain volume differences with larger kernel sizes. In the current study, we have used Pearson’s correlation to compare the spatial pattern of effect sizes between analyses, but future studies investigating the reliability of VBM parameters may consider using the intraclass correlation instead [55, 56].

### Limitations

The ENIGMA-VBM tool is designed to run locally at each site, meaning analyses are prespecified, which means we did not examine the interaction between PTSD and sex. Greater consideration of sex is required in future work [57], given the evidence for sex differences in PTSD prevalence [22, 23], symptom presentation [29, 32], and associated risk factors [58]. We were also unable to consider the type or incidence of trauma exposure, or the age of PTSD onset, as not all studies collected these data. It would be beneficial if these variables could be included in future studies, given the complexities surrounding the timing and experience of trauma in relation to the onset and severity of PTSD [59]. The majority of our studies were recruited in the United States, which limits the generalizability of our results, particularly given differences in PTSD prevalence [60] and in the types of commonly reported traumatic events [1] across countries. The current study is based on cross-sectional data, making it unclear whether the observed structural abnormalities represent vulnerability factors for PTSD and/or are consequences of the illness, which can be clarified with longitudinal studies.

We have conducted the largest PTSD meta-analysis to date using whole-brain VBM statistical maps, further strengthened in the homogeneity of the VBM processing pipeline via the ENIGMA-VBM tool. The 3D effect size and statistical maps from the current study are available online. Our results revealed that patients with PTSD exhibited smaller GM volumes across the brain as compared to controls and support the growing literature implicating the cerebellum in PTSD.

## Supporting information

10.1192/j.eurpsy.2025.10062.sm001 See et al. supplementary material 1 See et al. supplementary material

10.1192/j.eurpsy.2025.10062.sm002 See et al. supplementary material 2 See et al. supplementary material

## Data Availability

The 3D effect size maps and statistical maps are available online: https://neurovault.org/collections/QOAYXFZK/.

## Supplementary material

The supplementary material for this article can be found at http://doi.org/10.1192/j.eurpsy.2025.10062.
