# Supplementary material for: A whole-brain voxel-based analysis of structural abnormalities in PTSD: An ENIGMA-PGC study
Source: Eur Psychiatry. 2025 Jul 22;68(1):e97. doi: 10.1192/j.eurpsy.2025.10062 (PMC12344465; doi:10.1192/j.eurpsy.2025.10062)
Supplement: See et al. supplementary material 1 — See et al. supplementary material [file S092493382510062Xsup001.pdf]

Supplement A

Supplementary Methods 1
Treatment of the Clinical Variables 1
Parcel-based correlation analysis 2
Cohort-level details 3
Table S1. Cohort site and study details. 3
Table S2. Study inclusion and exclusion criteria. 4
Table S3. Scanner image acquisition and processing details for each cohort. 12
Table S4. Cohort-level clinical characteristics for PTSD severity, depression severity, and childhood trauma for the patient group. 14
Table S5. Cohort-level clinical characteristics for alcohol use disorder, drug use disorder, and antidepressant medication use for the patient group. 16
Supplementary Results 18
Summary Results Table. Summary table of all voxel-based analysis presenting the peak coordinate with the largest effect and a snapshot of the axial slice at the peak coordinate. 18
Group Comparisons 22
Table S6. PTSD vs. Controls 22
Figure S1. PTSD vs. Controls – differences in WM volumes 22
Figure S2. Forest plot comparing total GM volumes between PTSD patients and controls 23
Figure S3. Forest plot comparing total WM volumes between PTSD patients and controls 24
Table S7. PTSD vs. Trauma-Exposed Controls 25
Figure S4. PTSD vs. Trauma-Exposed Controls - differences in GM and WM 25
Table S8. Military-recruited cohorts 26
Figure S5. Military-recruited cohorts – differences in GM and WM 26
Table S9. Civilian-recruited cohorts 27
Figure S6. Civilian-recruited cohorts – differences in GM and WM 27
Regression Analyses of Clinical Variables 28
Table S10. PTSD severity associations with brain volume 28
Table S11. Depression severity associations with brain volume 28
Table S12. Alcohol use disorder associations with brain volume 29
Figure S7. Alcohol use disorder associations with brain volume – GM associations 29
Table S13. Antidepressant medication use associations with brain volume 30
Figure S8. Antidepressant medication associations with brain volume – GM associations 30
Sensitivity Analyses: exclusion of non-adults and adults with traumatic brain injury (TBI) 31
Table S14. PTSD vs. Controls excluding two non-adult cohorts 31
Figure S9. PTSD vs. Controls excluding two non-adult cohorts – differences in GM and WM 31
Table S15. PTSD vs. Controls excluding participants with traumatic brain injury 32
Figure S10. PTSD vs. Controls excluding participants with traumatic brain injury – differences in GM and WM 32

Sensitivity Analyses: controlling for different covariates **33**
Table S16. Covarying for age, ICV, and sex 34
Figure S11. Covarying for age, ICV, and sex – GM and WM differences 34
Table S17. Covarying for age and total GM or total WM 35
Figure S12. Covarying for age and total GM or total WM – GM and WM differences 35
Table S18. Covarying for age, age^2, ICV, and sex 36
Figure S13. Covarying for age, age^2, ICV, and sex – GM differences 36
Table S19. Covarying for age and sex 37
Figure S14. Covarying for age and sex – GM and WM differences 37
Table S20. Covarying for ICV 38
Figure S15. Covarying for ICV – GM and WM differences 38
Table S21. Proportional scaling covarying for age and ICV 39
Figure S16. Proportional scaling covarying for age and ICV – GM and WM differences 39
Table S22. No covariates 40
Figure S17. No covariates – GM and WM differences 40
Sensitivity Analyses: using non-modulated images **41**
Table S23. Non-modulated images covarying for age and ICV 41
Figure S18. Non-modulated images covarying for age and ICV – GM and WM differences 41
Table S24. Non-modulated images with no covariates 42
Figure S19. Non-modulated images with no covariates – GM and WM differences 42
Sensitivity Analyses: varying smoothing kernel size **43**
Table S25. Smoothing kernel of 2mm covarying for age and ICV 43
Figure S20. Smoothing kernel of 2mm covarying for age and ICV – GM differences 43
Table S26. Smoothing kernel of 4mm covarying for age and ICV 44
Figure S21. Smoothing kernel of 4mm covarying for age and ICV – GM differences 44
Table S27. Smoothing kernel of 12mm covarying for age and ICV 45
Figure S22. Smoothing kernel of 12mm covarying for age and ICV – GM and WM differences 45
Sensitivity Analysis: comparison to the main results **46**
Table S28. Correlations between the results of the sensitivity analyses and the main results. 46
Heterogeneity of Effect Size **47**
Figure S23. The heterogeneity of effect size measured using the I^2 statistic for the main group comparison. 47
Supplementary References **48**

Supplementary Methods

Treatment of the Clinical Variables

This section provides more detail on the treatment of the clinical variables. In general, continuous variables such as the PTSD severity, depression severity, and childhood trauma scores were used in their raw form as the regression analyses were done within each cohort, so it was not necessary to harmonise the data. The exception to this was where different scales were used within a cohort (e.g., CAPS-4 and CAPS-5). In these rare exceptions, cohorts may have consisted of samples from more than one study, but always from the same site and where possible, matched on scanner models, to maximise data retention while ensuring there were sufficient PTSD patients and controls for the analysis. Here, severity scores for each participant were calculated as a percentage of the possible maximum total score for the relevant scale instead as seen in previous ENIGMA-PTSD studies [1, 2].

Alcohol Use Disorder. Alcohol use disorder was coded as a binary variable (0 = no harmful alcohol use; 1 = harmful alcohol use). Group membership for alcohol use disorder was established using either SCID or MINI diagnostic criteria, or the recommended threshold cut-off score where the Alcohol Use Disorder Identification Test (AUDIT) [3] was used. Where participants were above the threshold scores for harmful alcohol use (AUDIT ≥ 8), they were coded as "1", otherwise they were coded as "0".

Drug Use Disorder. Drug use disorder was coded as a binary variable (0 = low/no drug use; 1 = drug use disorder). Group membership for drug use disorder was established using either SCID or MINI diagnostic criteria, or threshold cut-off scores where the Drug Abuse Screening Test (DAST) [4] was used. The threshold scores were set at the 'intermediate' level (DAST-28 ≥ 6; DAST-20 ≥ 6; DAST-10 ≥ 3), where participants above this threshold would be coded as "1", and below this threshold they would be coded as "0" [5].

Antidepressant Medication Use. Antidepressant medication use was coded as a binary variable (0 = not taking antidepressant medication; 1 = currently taking antidepressant medication). Group membership was derived from data collected for a broader field covering current medication, where available. This data were captured in free-text format, and as such any record of antidepressant medication was coded as "1", no mention was coded as "0", and missing data was coded as "NA".

In the regression analyses, cohorts were included where patients had sufficient clinical data, and for the categorical variables, there was sufficient variation across patients within a cohort. In other words, if a cohort had a specified exclusion criteria where all participants were naïve to drug use, then drug use disorder would be coded as "0" across all patients. This cohort would not be included in the analysis

because there would be no variation within the cohort, and it would not be possible to conduct a regression analysis.

The cohort-level characteristics and the measurement instruments used for each clinical variable is reported in Tables S4 and S5.

Parcel-based correlation analysis

To compare the spatial pattern of regional GM and WM differences between a given sensitivity analysis and our main group findings, we used a parcel-based correlation analysis in R (version 4.3.1) and the packages *nifti.oro* [6] and *nifti.pbcor* (https://CRAN.R-project.org/package=nifti.pbcor) [7]. This approach mitigates the issue in voxel-based correlations where adjacent voxels are not independent. The correlation analysis is done by randomly dividing the brain into parcels and calculating the Pearson's correlation coefficient across parcels. The random parcellation and correlation is performed multiple times such that the final result is the median estimate of the correlation coefficients calculated. A correlation coefficient of 0 indicated there was no similarity between the effect size maps, while a value of 1 indicated the maps were perfectly correlated.

Cohort-level details

Table S1. Cohort site and study details.

Site	PI(s)	Cohorts[1]	Study Name	City	Country
ADNI-DoD	P. Thompson	ADNIDOD 1<br>ADNIDOD 2	ADNI-DoD	Marina del Rey, CA	United States
Academic Medical Centre	M. Olff<br>D.J. Veltman	AMC	BOOSTER	Amsterdam	Netherlands
Beijing	L. Wang	Beijing	Wenchuan Earthquake Study	Beijing	China
Columbia University	Y. Neria<br>X. Zhu	Columbia-3	Columbia-3	New York City, NY	United States
		Columbia-6	Columbia-6	New York City, NY	United States
Duke University	R. Morey	Duke 1	CatGen	Durham, NC	United States
			SubBlast	Durham, NC	United States
			TBIPTSD	Durham, NC	United States
		Duke 2	Predator-1	Durham, NC	United States
			Predator-3	Durham, NC	United States
		Duke 3	FearPTSD	Durham, NC	United States
		Duke 4	MIRECC	Durham, NC	United States
Emory	J.S. Stevens<br>N. Fani	Emory	Grady Trauma Project	Atlanta, GA	United States
INTRuST	M.B. Stein	INTRuST 1<br>INTRuST 2	INTRuST	Multiple	United States
Leiden University Medical Center	N.J.A. van der Wee	Leiden	EPISCA	Leiden	Netherlands
LIMBIC-CENC	E.L. Dennis	LIMBIC-CENC 1<br>LIMBIC-CENC 2<br>LIMBIC-CENC 3	LIMBIC-CENC	Richmond, VA, Houston, TX, Tampa, FL, San Antonio, TX, Ft. Belvoir, FL, Portland, OR, Minneapolis, MN	United States
McLean Hospital	M. Kaufman	McLean 1	NTD	Boston, MA	United States
	I. Rosso	McLean 2	McLean Rosso	Boston, MA	United States
University of Minnesota	S. Lissek	Minnesota	MARS2	Minneapolis, MN	United States
University Hospital Münster	T. Straube<br>D. Hofmann	Münster	Münster	Münster	Germany
University of South Dakota	L.A. Baugh<br>K. A. Fercho	South Dakota	PTSD	Vermillion, SD	United States
			SAP	Vermillion, SD	United States
Stanford University	A. Etkin<br>A. Maron-Katz	Stanford	CausCon	Palo Alto, CA	United States
University of Toledo	X. Wang	Toledo	ONG	Toledo, OH	United States
			MVA	Toledo, OH	United States
University of Cape Town	D.J. Stein<br>J. Ipser	UCT	Drakenstein Child Health Study	Cape Town	South Africa
University Medical Center	E. Geuze	UMC BETTER	BETTER	Utrecht	Netherlands
VA Minneapolis	S. Sponheim	VA Minn DEFEND	DEFEND	Minneapolis, MN	United States
		VA Minn SATURN	SATURN	Minneapolis, MN	United States
VA Waco	E. Gordon<br>G. May	VA Waco	MAVERIX	Waco, TX	United States
			ROBI	Waco, TX	United States
			TEMI	Waco, TX	United States
VA West Haven	C.G. Abdallah	VA West Haven	West Haven	West Haven, CT	United States
Vanderbilt	J.U. Blackford	Vanderbilt	Vanderbilt PTSD Study	Nashville, TN	United States
University of Washington	K. McLaughlin	Washington	UwashMT	Seattle, WA	United States
Lawson Health Research institute	R. Lanius	Western Ontario	Western Ontario	London, ON	Canada
University of Wisconsin-Madison	D.W. Grupe	Wisconsin-Madison	Veterans' Wellness Study	Madison, WI	United States
University of Wisconsin-Milwaukee	C. Larson	Wisconsin-Milwaukee	iSTAR	Milwaukee, WI	United States
Yale University	I. Harpaz-Rotem	Yale	Yale	New Haven, CT	United States

Organised alphabetically by Cohort name.

[1] Studies within each site may have been combined into different processing cohorts based on scanner model to minimise the effects of scanner during the ENIGMA-VBM processing, or to ensure enough patients and controls for analysis.

Table S2. Study inclusion and exclusion criteria.

Cohorts	Study Name	Inclusion Criteria	Exclusion Criteria
ADNIDOD 1<br>ADNIDOD 2	ADNI-DoD	All: Vietnam War veterans 50-90 years of age, must live within 150 miles of the closest ADNI clinic<br>PTSD: Must meet SCID-I (for DSM-IV-TR) criteria for current/chronic PTSD: current CAPS-IV>49; current PTSD symptoms related to a Vietnam War related trauma<br>Control: Must be comparable in age, gender, and education with TBI and PTSD groups. May be receiving VA disability payments for something other than TBI or PTSD – or no disability at all.	All: Mild Cognitive Impairment/Dementia; Documented or self-report history of mild/moderate severe TBI; Any history of head trauma associated with persistent cognitive complaints or loss of consciousness >5minutes; History of psychosis, bipolar disorder, alcohol and/or substance abuse/dependence within past 5 years; contraindications to MRI, lumbar puncture, PET scan; unstable medical conditions (e.g., hepatic, renal, pulmonary, metabolic diseases);<br>Control: MCI/Dementia; Current or lifetime presence of PTSD (DSM-IV-TR criteria or a CAPS-IV>30)
AMC	BOOSTER	All: Police officers 18-65 years of age who are eligible for MRI<br>PTSD: current PTSD diagnosis, with CAPS ≥ 45.<br>Controls: exposure to at least one traumatic event (according to DSM-IV A1 criterion), with CAPS < 15	General: History of neurological disorders, any severe or chronic systemic disease or unstable medical condition (including endocrinological disorders), use of psychotropic medications.<br>Females: pregnancy or breastfeeding.<br>PTSD: current psychotic disorder, substance-related disorder, severe personality disorder, severe major depressive disorder (MDD) (i.e., involving high suicidal risk and/or psychotic symptoms) or current suicidal risk.<br>Controls: any current Axis-1 disorder and lifetime history of PTSD or MDD
Beijing	Wenchuan Earthquake Study	Individuals 18-65 years of age who personally experienced Wenchuan earthquake in 2008 and are right-handed	Intellectual disability; major psychosis (e.g., schizophrenia and organic mental disorders); drug or alcohol abuse; history of head trauma or surgery; metallic embedded object in body; claustrophobia; exposure to other trauma events from time of the disaster to the time of the study.
Columbia-3	Columbia-3	PTSD: Criterion A trauma. CAPS-4 diagnosis of PTSD, CAPS score of 50 or above.	For patients, psychosis, substance/alcohol dependence within 6 months or abuse within 2 months, use of psychotropic medication in past 4 weeks (6 weeks of fluoxetine), HAM-D-17 score greater than 24. For controls, current or past Axis I disorder or CAPS > 19.
Columbia-6	Columbia-6	All: Males or females 18-60 years of age able to give consent, fluent in English<br><br>PTSD: Experience of a traumatic event or events during lifetime; current DSM-V Criterion A for PTSD.	All: Prior or current Axis I psychiatric diagnosis of schizophrenia, psychotic disorder, bipolar disorder, dementia; depression score of > 25 on the Hamilton Rating Scale for Depression (HAM-D-17-item); significant depression and /or depression related impairment that is judged to warrant pharmacotherapy or combined medication and psychotherapy; individuals at risk for suicide based on history and current mental state; history of substance/alcohol dependence within the past six months, or abuse within past two months; any psychotropic medications; pregnancy, or plans to become pregnant during the period of the study; paramagnetic metallic implants or devices contraindicating magnetic resonance imaging or any other non-removable paramagnetic metal in the body; medical illness that could interfere with assessment of diagnosis, or biological measures (SCR, fMRI), including organic brain impairment from stroke, CNS tumour, or demyelinating disease, and renal, thyroid, hematologic or hepatic impairment; any condition that would exclude MRI exam (e.g., pacemaker, paramagnetic metallic prosthesis, surgical clips, shrapnel, necessity for constant medicinal patch, some tattoos).

Table S2. Study inclusion and exclusion criteria (*continued)*.

Cohorts	Study Name	Inclusion Criteria	Exclusion Criteria
Duke 1	CatGen	Veterans 18-65 years of age, fluent in English, free of implanted metal objects or metal shards in eyes	Axis I disorders (except depression, GAD, PTSD, panic disorder, agoraphobia, other specific phobias, anxiety NOS), ferrous metal in the body, neurological disorders, history of TBI, colour blindness, psychotic disorders, suicide attempts in past year, claustrophobia.
	SubBlast	Veterans 18-65 years of age, fluent in English, free of implanted metal objects or metal shards in eyes	Axis I disorders (except depression, GAD, PTSD, panic disorder, agoraphobia, other specific phobias, anxiety NOS), ferrous metal in the body, neurological disorders, history of TBI, colour blindness, psychotic disorders, suicide attempts in past year, claustrophobia.
	TBIPTSD	Veterans 18-65 years of age, fluent in English, free of implanted metal objects or metal shards in eyes	Axis I disorders (except depression, GAD, PTSD, panic disorder, agoraphobia, other specific phobias, anxiety NOS), ferrous metal in the body, neurological disorders, history of TBI, colour blindness, psychotic disorders, suicide attempts in past year, claustrophobia.
Duke 2	Predator-1	Veterans 18-65 years of age, fluent in English, free of implanted metal objects or metal shards in eyes	Axis I disorders (except depression, GAD, PTSD, panic disorder, agoraphobia, other specific phobias, anxiety NOS), ferrous metal in the body, neurological disorders, history of TBI, colour blindness, psychotic disorders, suicide attempts in past year, claustrophobia.
	Predator-3	Veterans 18-65 years of age, fluent in English, free of implanted metal objects or metal shards in eyes	Significant neurological disorders, a history of learning disability, developmental delay, current substance abuse, a history of substance dependence, psychotic disorders, significant medical conditions, suicide attempt during the past year or are currently at high risk for suicide, neurological injury, or disease (head trauma, seizures, strokes, prior neurosurgery, or if they are under the care of a neurologist or neurosurgeon), pregnant women, MRI contraindications.
Duke 3	FearPTSD	Veterans 18-65 years of age, fluent in English, free of implanted metal objects or metal shards in eyes	Axis I disorders (except depression, GAD, PTSD, panic disorder, agoraphobia, other specific phobias, anxiety NOS), ferrous metal in the body, neurological disorders, history of TBI, colour blindness, psychotic disorders, suicide attempts in past year, claustrophobia.
Duke 4	MIRECC	Veterans 18-65 years of age, fluent in English, free of implanted metal objects or metal shards in eyes	Axis I disorders (except depression, GAD, PTSD, panic disorder, agoraphobia, other specific phobias, anxiety NOS), ferrous metal in the body, neurological disorders, history of TBI, colour blindness, psychotic disorders, suicide attempts in past year, claustrophobia.
Emory	Grady Trauma Project	Individuals 18-65 years of age who speak English and have endorsed at least 1 criterion A trauma	Current psychotic symptoms or bipolar disorder; current substance or alcohol dependence; history of head trauma; psychoactive medication usage; current illegal drug use (verified with urine drug screen within 24 hours of scan)

Table S2. Study inclusion and exclusion criteria (*continued)*.

Cohorts	Study Name	Inclusion Criteria	Exclusion Criteria
INTRuST 1<br>INTRuST 2	INTRuST	Patients: enrolled in individual INTRuST studies with a diagnosis of mTBI (initial Glasgow Coma Scale score of 13-15) or diagnosis of current psychological distress (PTSD, anxiety, or depression), or both.<br><br>Healthy Controls: ages between 18 and 65.	Patients: (1) lifetime bipolar I disorder, lifetime psychotic disorders, lifetime dementia, delirium, alcohol or other substance dependence (within 30 days), (2) CNS disorders including aneurysm, anoxic events, brain tumour, encephalitis, Guillain Barre syndrome, Huntington's disease, hydrocephalus, uncontrolled diabetes, thyroid condition or blood pressure, multiple sclerosis, Parkinson's disease, seizure disorder, stroke, or subdural hematoma, (3) currently pregnant or lactating (due to effects of hormonal fluctuations on biological samples collected as part of the repository). (4) current medications that affect the brain function as determined by the study physician, (5) English as a second language after the age of 5, (6) history of a learning disability, and (7) weight of more than 300 pounds as this would preclude the subject from entering the scanner.<br>Healthy Controls: screened by phone and by an in-person MINI (6.0.0) interview. (1) CNS disorders as described above, (2) medication exclusions, including more than one antihypertensive drug, psychotropic drugs within the last 90 days, herbal psychoactive substance use, or steroid use in the last 4 months, (3) currently pregnant or lactating, (4) history of mood, anxiety, psychotic, dementia, delirium, substance dependence in the past 12 months, (5) history of probable TBI as defined by the I-TBI.
Leiden	EPISCA	All participants met the following inclusion criteria: aged between 12 and 21, estimated full scale IQ (FIQ) ≥ 80 as measured by Dutch versions of the Wechsler Intelligence Scales for Children (WISC-III) or adults (WAIS), being right-handed, normal or corrected-to-normal vision, sufficient understanding of the Dutch language, no history of neurological impairments and no contraindications for MRI testing (e.g. braces, metal implants or possible pregnancy).	(1) Primary DSM-IV diagnosis of ADHD, pervasive developmental disorders, Tourette's syndrome, obsessive–compulsive disorder, bipolar disorder, and psychotic disorders; (2) current use of psychotropic medication other than stable use of SSRI's, or amphetamine medication on the day of scanning; and (3) current substance abuse.
LIMBIC-CENC 1<br>LIMBIC-CENC 2<br>LIMBIC-CENC 3	LIMBIC-CENC	Veterans with history of deployment in Operation Enduring Freedom (OEF), Operation Iraqi Freedom (OIF), Operation New Dawn (OND), or follow-up conflicts; history of combat exposure (score>1 on any item in Deployment Risk and Resiliency Inventory Section D [DRRI-2-D])	History of moderate to severe TBI; history of major neurologic disorder with significant decrease in functional status and/or loss of ability for independent living; severe psychiatric disorder (e.g., schizophrenia)
McLean 1	NTD	Women 18-60 years of age with a history of childhood maltreatment who speak English; must have legal and mental competency, Normal or Corrected Vision.	Delirium secondary to medical illness; History of neurological conditions that may cause significant psychiatric symptomatology (e.g., dementia); Any contraindication to MR scans, including claustrophobia, pregnancy, metal implants, etc.; Current alcohol or substance use disorder (within the last month); A history of schizophrenia or other psychotic disorder; History of head injury or loss of consciousness for longer than 5 min (including concussion); pregnancy

Table S2. Study inclusion and exclusion criteria (*continued)*.

Cohorts	Study Name	Inclusion Criteria	Exclusion Criteria
McLean 2	McLean Rosso	Civilians aged 20-50 years old; right-handed; DSM-IV diagnosis consistent with group assignment; ability to provide written informed consent	Medical condition that would confound results; history of seizures or head trauma with loss of consciousness; exposure to psychotropic medications within 4 weeks of study (8 weeks for fluoxetine); contraindications to MRI; positive urine toxicology or HCG status on scan day; history of psychotic disorder, bipolar disorder, eating disorder, intellectual disability, or pervasive developmental disorder; lifetime history of DSM-IV non-PTSD anxiety disorder
Minnesota	MARS2	Individuals 18-65 years of age with history of combat-related trauma	Current of past history of psychosis, bipolar disorder, delirium, dementia, amnestic disorder, or intellectual disability; suicidality; substance use disorder within past six months; pregnancy; current or past medical illnesses that may confound study results or place participant at risk; current use of any medication that alters central nervous system function including antidepressants, benzodiazepines, anti-psychotics, mood-stabilizers, anti-parkinsonian agents, anti-convulsants, sleep medications, pain medications, and anti-hypertensives; MRI contraindications
Münster	Münster	All patients fulfilled the diagnostic criteria for PTSD as primary diagnosis according to the DSM-IV-TR (American Psychiatric Association, 2000), assessed by the German version of the Structured Clinical Interview for DSM-IV (SCID; Wittchen et al., 1997). Given the focus on InterPersonal Violence-PTSD (IPV-PTSD), the experience of a trauma related to IPV (e.g., rape, sexual or physical abuse) at least once was an inclusion criterion for the patient group. All participants had normal or corrected-to-normal vision and were right-handed as determined by the Edinburgh Handedness Inventory (Oldfield, 1971).	Controls: Lifetime PTSD.
South Dakota	PTSD	OIF/OEF/OND (Operation New Dawn) veterans.	Exclusion criteria were (a) current or previous seizure history; b) current crisis-related issues such as serious self-injurious behaviour, psychosis, or substance dependence (excluding alcohol dependence); (c) report of traumatic brain injury using the Traumatic Brain Injury Checklist; and (d) contraindications to fMRI (metal objects in body, claustrophobia).
	SAP	Participants were undergraduate students who were identified as an adult child of an alcoholic parent (ACoA), based on the Children of Alcoholics Screening Test (CAST, Jones, 1983). A score of 6 or above on the CAST indicated the participant was more than likely the child of an alcoholic parent and raised by this parent.	Participants were excluded for current or previous seizure history, contraindications to MRI, or if they exhibited possible psychotic or other psychological symptoms that would make inclusion in the study potentially hazardous to them.

Table S2. Study inclusion and exclusion criteria (*continued)*.

Cohorts	Study Name	Inclusion Criteria	Exclusion Criteria
Stanford	CausCon	Patients will be required to have chronic (>3 months) moderate to severe anxiety or depression, assessed dimensionally by a score on the PHQ9 scale (excluding the suicide question)>10 or a score on the GAD7 scale >10. Both of these scales assess general symptoms of anxiety and depression, and these cut-offs have been shown to relate to moderate or greater severity of symptoms. Moreover, because these scales measure general anxiety and depression, they are sensitive to a wide range of DSM diagnoses, including GAD, MD, and PTSD. Additionally, to ensure clinical significance, subjects will need to indicate that they would be interested in seeking treatment for these symptoms (i.e. that symptoms impair functioning). Other inclusion criteria are: (1) community dwelling adults ages 18-60 years old; (2) not currently in treatment; (3) free of metal or ferrous implant; (4) good English comprehension and non-impaired intellectual abilities to ensure understanding of task instructions; (5) no history of neurological disorders, brain surgery, electroconvulsive or radiation treatment, brain haemorrhage or tumor, stroke, epilepsy, hypo- or hyperthyroidism; and (6) no daily use of PRN benzodiazepines or opiates(max: 3x/week), or daily thyroid medications, and no antidepressant, anticonvulsant or antipsychotic medications for >2 weeks (fluoxetine >6 weeks). As-needed benzodiazepines or opiates cannot be used within 48 hours of assessments. Medication-free healthy subjects will likewise be split equally between those who have never been traumatized and those who have had a criterion A trauma. Controls must deny lifetime psychiatric diagnosis and treatment and have PHQ9 and GAD7 ≤4. Stratification of each group by trauma exposure will be re-assessed every 20 participants and we will ensure that groups are matched on demographic variables.	(1) MRI counter-indications (e.g. shrapnel or other metal in/on the body that cannot be removed, claustrophobia, etc.); (2) Additional TMS counter-indications (seizure disorder, CNS active disorder, certain medications described below); (3) Medication use that substantially reduces seizure threshold to TMS (olanzapine, chlorpromazine, lithium) and unwilling or unable medically (determined by patient and his/her physician) to safely withdraw, at least 2 weeks prior to TMS, from these medications; (4) Opiate medication, antihypertensive medication, or any medication that interferes with blood flow (interferes with fMRI recordings); (5) Thyroid dysfunction not adequately controlled by medication; (6) History of neurological or cardiovascular disorders, brain surgery, radiation treatment, brain haemorrhage or tumour, stroke, or diabetes; (7) Diagnosis of substance dependence within the past 3 months (but not abuse); (8) Refusal to abstain from illicit drug use for duration of the study; (9) Refusal to abstain from alcohol within 24 hours of scans; (10) Pregnancy in female participants; (11) Prior exposure to deep brain stimulation, rTMS, or tDCS (transcranial direct current stimulation) therapies; (12) Significant traumatic brain injury (loss of consciousness, post-injury amnesia, significant radiological/neurological findings, penetrating brain injury); (13) Lifetime evidence of psychosis, mania, hypomania, or bipolar disorders on the SCID.

Table S2. Study inclusion and exclusion criteria (*continued)*.

Cohorts	Study Name	Inclusion Criteria	Exclusion Criteria
Toledo	ONG	Ohio National Guard and Reserve soldiers 18-50 years of age who were deployed in OEF or OIF – must have met Ohio National Guard Study characteristics and able to provide informed consent.	History of psychosis, bipolar disorder, or neurologic condition; current substance dependence; intellectual disability or developmental disorder; contraindication to MRI; current use of antipsychotic medication.
	MVA	Motor vehicle accident (MVA) survivors transported to the University of Toledo Emergency department, or to a ProMedica emergency medicine department.	Pregnancy; under the influence of alcohol or drugs at the time of MVA; major injuries, moderate to severe traumatic brain injury; major medical illnesses; contraindication to MRI
UCT	Drakenstein Child Health Study	Women over the age of 18 years, who were between 20 and 28 weeks pregnant at the time of initial inclusion in the study, who presented to one of two health care clinics for antenatal care (TC Newman and Mbekweni clinics), and had no intention of moving out of the area within the following year, and were able to give written consent.	1) Loss of consciousness longer than 30 minutes; 2) inability to speak English; 3) current/lifetime alcohol and/or substance dependence or abuse; 4) psychopathology other than PTSD and/or MDD; 5) traumatic brain injury; 6) standard MRI exclusion criteria, such as claustrophobia and presence of ferromagnetic objects in the participant's body.
UMC BETTER	BETTER	Age 18–60 years and written informed consent. War veterans with PTSD: diagnosed with combat-related PTSD by a psychologist or psychiatrist at one of the four Military Mental Healthcare out-patient clinics. This was confirmed with a total score of $\geqslant$45 on the clinician-administered PTSD scale. Controls consisted of war veterans without a current psychiatric disorder and non-military participants without a current psychiatric disorder. Controls were included when they had no current psychiatric disorder and a CAPS total score of $\leqslant$15.	A history of neurological illness.
VA Minn DEFEND	DEFEND	Age: 18-60, OEF/OIF, deployed, positive screen on VA TBI Clinical Reminder.	Moderate/severe TBI, non-TBI neurological conditions, current psychotic symptoms, substance abuse/dependence other than alcohol, unstable med conditions, significant risk of suicide/homicide.
VA Minn SATURN	SATURN	Age: 18-60, OEF/OIF, deployed.	Moderate/severe TBI, non-TBI neurological conditions, current psychotic symptoms, substance abuse/dependence other than alcohol, unstable med conditions, significant risk of suicide/homicide.

Table S2. Study inclusion and exclusion criteria (*continued)*.

Cohorts	Study Name	Inclusion Criteria	Exclusion Criteria
VA Waco	MAVERIX	Veteran, age 18-60, agreement to donate saliva.	Serious general medical condition that would risk the subject being able to complete MRI (active seizure disorder, dementia, active back or muscle spasms), MRI safety screen positive (metal) or history of penetrating head or eye wound without subsequent radiological evidence that the wound is metal-free. Subjects that are (were) welders or subjects that have had metal surgically removed from their eyes will not be allowed to participate without subsequent radiological evidence that the wound is metal-free, MRI quality problems (tremors, significant claustrophobia, teeth braces).
	ROBI	Participants will be Veterans with a diagnosis of TBI, recruited on a volunteer basis from the Central Texas VA. Inclusion criteria are age of 18-60 years and a clinical diagnosis of TBI in the VA medical record.	Exclusion criteria will include: an absence of qEEG parameters more than 2 standard deviations from the population mean of healthy age-matched historical controls saved in a commercial normative database (Neuroguide, Largo, FL); a positive screen on the MINI International Neuropsychiatric Interview: diagnosis of schizophrenia, schizoaffective disorder, bipolar disorder type I, severe substance use disorder, a high risk of suicide; and an inability to provide informed consent.
	TEMI	Male and female Veterans enrolled in a CTVHCS PTSD treatment program who are 18-60 years old.	(1) pregnancy; (2) exposure to metal in the eyes; (3) shrapnel or other metal embedded in the body; (4) ferromagnetic surgical implants; (5) mechanical implants (e.g., pacemakers); (6) electrical implants (e.g., cochlear implants); (7) non-removable metallic devices (e.g. stables, neck braces, or artificial limbs) (8) tattoos not done professionally; (9) non-removable body piercings; (10) current psychosis including Axis I psychotic disorder, bipolar disorder, or schizophrenia; (11) dementia or another severe cognitive disorder; (12) prior exposure to an rTMS or dTMS; (13) seizure disorder; (14) positive screen for suicidal intent, plan, or behaviour within the past 6 months; (15) a TMS motor threshold of 70% or greater of the machine's maximum output.
VA West Haven	West Haven	US Combat Veterans with and without PTSD, age 21-65, who were fluent in English. Comorbidities such as unipolar depression, anxiety disorders, substance/alcohol use disorders, and a stable dose of antidepressants were allowed.	Psychotic disorder, bipolar depression, learning disorder, attention deficit disorder/hyperactivity disorder, moderate-to-severe TBI, epilepsy, brain tumours, gross neurological disorders, benzodiazepines, standard MRI contraindications.
Vanderbilt	Vanderbilt PTSD Study	OEF/OIF/OND Veterans 18-50 years of age who are fluent in English	Psychoactive medication usage in past 6 weeks; participated in psychotherapy within the past month; current substance use disorder (>6 month remission); positive urine drug or alcohol breath screen on MRI study day; history of psychotic or bipolar disorder, traumatic brain injury, or significant medical (e.g., cancer, HIV) or neurological illness (e.g., stroke, brain tumor, multiple sclerosis, epilepsy); contraindication to MRI<br><br>Trauma-exposed controls: lifetime diagnosis of PTSD; symptoms of hypervigilance<br><br>Healthy controls: any trauma exposure

Table S2. Study inclusion and exclusion criteria (*continued)*.

Cohorts	Study Name	Inclusion Criteria	Exclusion Criteria
Washington	UwashMT	8-20 years old English speaking.	Psychiatric medication use (excepting stimulant meds for ADHD, which were discontinued for the scan). MRI contra-indications including braces, or other metal in the body or claustrophobia. Active substance dependence, pervasive developmental disorder, active safety concerns.
Western Ontario	Western Ontario	Primary diagnosis of PTSD for patients	Incompatibilities with scanning conditions, previous neurologic and development illness, comorbid schizophrenia or bipolar disorder, alcohol or substance abuse, a history of head trauma, or pregnancy during scan. Participants were excluded if they had implants or metal that do not comply with 3T fMRI safety standards for research, a history of head injury with a loss of consciousness, significant untreated medical illness, a history of neurological disorders, history of any pervasive developmental disorders, pregnancy, and current use of any psychotropic medication within one month prior to study. PTSD individuals were further excluded if they reported a history of bipolar disorder, schizophrenia, or substance-use disorder prior to participation of the study.
Wisconsin-Madison	Veterans' Wellness Study	Age range of 18-50; Capable of giving informed consent; Fluent in English; Exposure to one or more life-threatening war zone trauma events per the Combat Experiences Scale and documented by DD-214, Combat Action Ribbon (Marines), Combat Infantry Badge (Army), or other clear evidence of war zone trauma exposure in Iraq or Afghanistan since 2001; Pharmacological or psychotherapeutic treatment stable for at least 8 weeks prior to beginning of study, with no intent to begin a new course of treatment during the study period.	Weight of 352 pounds or over (due to constraints of MRI scanner); Women of childbearing potential with positive pregnancy test, looking to conceive during the research timeline, or who are breastfeeding; Metallic implants such as prostheses or aneurysm clip, or electronic implants such as cardiac pacemakers; Neurological or serious medical condition that may contraindicate MRI or that may overlap with physiological substrates of psychiatric conditions; History of seizures or seizure disorder; Moderate or severe traumatic brain injury (over 20 minutes unconscious); Current active substance dependence or dependence within 3 months (other than nicotine); Meets DSM-IV criteria for bipolar disorder, schizophrenia, schizoaffective disorder, psychotic disorder NOS, delirium, or any DSM-IV cognitive disorder; Substance dependence disorder within 3 months or any current substance dependence; Severe psychiatric instability or severe situational life crises, including evidence of being actively suicidal or homicidal, or any behaviour that poses an immediate danger to patient or others; Participants with extensive experience in yoga or meditation; Current use of benzodiazepines and beta-blockers.
Wisconsin-Milwaukee	iSTAR	Civilians aged 18-60 years; exposure to DSM-5 A1 criterion trauma; high risk for PTSD (score ≥3 OR item 2 rated ≥3 on Predicting PTSD Questionnaire, Rothbaum et al., 2014); English speaking; ability to schedule baseline study visit within 30 days of traumatic injury	Glasgow Coma Scale score ≤ 13 (i.e., moderate to severe traumatic brain injury); on police hold; contraindication to MRI; pregnancy (or planned pregnancy within 6 months); intentional self-inflicted injury; severe vision or hearing impairment; history of psychotic or manic symptoms, or neurologic condition (e.g., seizures, spinal cord injury); currently on antipsychotic medication; clear evidence of substance use disorder
Yale	Yale	Individuals 21-60 years of age; at least one deployment on combat tour	Diagnosis of bipolar disorder or psychotic disorder; current benzodiazepine use; a history of ADHD, learning disorder, moderate or severe traumatic brain injury (TBI), brain tumor, epilepsy, or a neurological disorder; current inpatient status; MRI contraindication.

Table S3. Scanner image acquisition and processing details for each cohort.

Cohort	Scanner Model	Strength	No. Coil Channels	Sequence	Voxel Size (mm)	FOV (mm)	TR	TE	Flip Angle
ADNIDOD 1	GE Discovery MR750	3T	8	FSPGR	1 x 1 x 1.2	256 x 256	6984	2.85	11
	GE Discovery MR750w	3T	40	SPGR	1 x 1 x 1.2	256 x 256	7652	3.1	11
ADNIDOD 2	GE Signa HDxt	3T	8	SPGR	1 x 1 x 1.2	256 x 256	7340	3.04	11
AMC	Philips Achieva	3T	32	Fast MPRAGE	1 x 1 x 1	240 x 188	8200	3.8	8
Beijing	Philips Achieva	3T	8	NA	1 x 0.8594 x 0.8594	220 x 220	8500	3.7	90
Columbia-3	GE Signa Excite	1.5T	8	SPGR	1 x 1 x 1	224 x 224	3000	3	84
Columbia-6	GE MR750	3T	32	NA	1 x 1 x 1	NA	1300	2.8	60
	GE Premier	3T	32	NA	1 x 1 x 1	NA	1300	2.8	60
Duke 1	GE Discovery MR750	3T	8	FSPGR BRAVO	0.9375 x 0.9375 x 1	240 x 240	8160	8.148	12
	GE Discovery MR750	3T	8	FSPGR BRAVO	0.9375 x 0.9375 x 1	240 x 240	8160	3.22	12
	GE Signa Excite	3T	8	FSPGR BRAVO	0.9375 x 0.9375 x 1	240 x 240	8148 / 7840 / 8160	3.22	12
Duke 2	GE Discovery MR750	3T	8	FSPGR BRAVO	1 x 1 x 1	256 x 256	8160	3.18	12
Duke 3	GE Discovery MR750	3T	8	FSPGR BRAVO	0.9375 x 0.9375 x 1	256 x 256	8160	2.98	12
Duke 4	GE LX Nvi	4T	8	NA	1 x 1 x 1	240 x 240	NA	5.4	20
Emory	Siemens TIM Trio	3T	12	MPRAGE	1 x 1 x 1	224 x 256	2600	3.02	8
INTRuST 1	GE Discovery MR750	3T	NA	SPGR	1 x 1 x 1	256 x 256	9160	3.71 / 3.68	10
	Philips Achieva	3T	NA	T1W 3D TFE SENSE	1 x 1 x 1	256 x 256	7640 / 7670	3.56/ 3.53	7
INTRuST 2	Siemens TIM Trio	3T	NA	MPRAGE	1 x 1 x 1	256 x 256	2530	3.32	7
Leiden	Philips Achieva	3T	8	NA	1 x 1 x 1	224 x 177 x 168	9.8	4.6	8
LIMBIC-CENC 1	Philips Ingenia	3T	NA	MPRAGE	1 x 1 x 1	256 x 256	6.78	3.16	9
LIMBIC-CENC 2	Siemens TIM Trio	3T	NA	MPRAGE	1 x 1 x 1	240 x 256	2300	2.96	9
	Siemens Prisma	3T	NA	MPRAGE	1 x 1 x 1	300 x 320	2400	2.24	8
LIMBIC-CENC 3	GE Signa HDxt	NA	NA	SPGR	1 x 1 x 1	256 x 256	6.28	3.15	NA
McLean 1	Siemens TIM Trio	3T	32	MPRAGE	1.2 x 1.2 x 1.2	256 x 128	2530	3.31	7
McLean 2	Siemens TIM Trio	3T	12	MEMPRAGE	1 x 1 x 1	256 x 256	2530	1.64 / 3.5 / 5.36 / 7.22	10
Minnesota	Siemens Magnetom Prisma	3T	32	NA	1 x 1 x 1	NA	NA	NA	NA
Münster	Siemens Prisma	3T	32	MPRAGE	1 x 1 x 1	256 x 256	2130	2.28	8
South Dakota	Siemens Magnetom Skyra	3T	20	MPRAGE	0.9375 x 0.9375 x 0.9	240 x 240	1900	2.13	9
	Siemens Magnetom Skyra	3T	20	MPRAGE	0.9375 x 0.9375 x 0.9	256 x 256	1900	2.13	9
Stanford	GE Discovery MR750	3T	8	SPGR	1 x 0.9.375 x 0.9375	240 x 240	8600	3.4	15
Toledo	GE Signa HDxt	3T	8	SPGR	1 x 1 x 1	256 x 256	8200	3.2	12
	GE Signa HDxt	3T	8	SPGR	1 x 1 x 1	256 x 256	8200	3.2	12
UMC BETTER	Philips Achieva	3T	NA	3D-FSE	0.75 x 0.75 x 0.8	240 x 240 x 160	10	4.6	8

Table S3. Scanner image acquisition and processing details for each cohort (*continued)*.

Cohort	Scanner Model	Strength	No. Coil Channels	Sequence	Voxel Size (mm)	FOV (mm)	TR	TE	Flip Angle
UCT	Siemens Skyra	3T	32	MPRAGE	1.5 x 1 x 1	256 x 256	2530	1.69 / 3.55 / 5.41 / 7.27	7
	Siemens Allegra	3T	4	MPRAGE	1.5 x 1 x 1	256 x 256	2000	1.53 / 3.21 / 4.89 / 6.57	20
VA Minn DEFEND	Siemens TIM Trio	3T	12	MPRAGE	1 x 1 x 1	256 x 256	2530	3.65	7
VA Minn SATURN	Siemens TIM Trio	3T	12	MPRAGE	1 x 1 x 1	256 x 256	2530	3.65	7
VA Waco	Philips Achieva	3T	16	MPRAGE	0.9 x 0.9 x 0.9	256 x 256	7256	2.77	12
	Philips Achieva	3T	16	MPRAGE	0.9 x 0.9 x 0.9	256 x 256	7256	2.77	12
	Philips Achieva	3T	16	MPRAGE	0.9 x 0.9 x 0.9	256 x 256	7256	2.77	12
VA West Haven	Siemens TIM Trio	3T	32	MPRAGE	1 x 1 x 1	256 x 256	2530	2.71	7
Vanderbilt	Philips Intera	3T	32	NA	0.8 x 0.8 x 0.9	256 x 256	9000	4.6	9
Washington	Philips Achieva	3T	32	MPRAGE	1 x 1 x 1	256 x 256	2530	1.6 - 7	7
Western Ontario	Siemens Biograph mMR	3T	32	MPRAGE	1 x 1 x 1	256 x 240 x 192	2300	2.98	9
Wisconsin-Madison	GE Discovery X750	3T	8	MPRAGE	1 x 1 x 1	256 x 256	1900	2.5	9
Wisconsin-Milwaukee	GE Discovery MR750	3T	8	MPRAGE	0.9375 x 0.9375 x 1	256 x 256	8200	3.2	12
Yale	Siemens TIM Trio	3T	12	MPRAGE	1 x 1 x 1	256 x 256	2500	2.77	7

FOV = Field of View; TR = Repetition Time; TE = Echo Time; NA = Not Available

FSPGR = fast spoiled gradient echo; SPGR = spoiled gradient recalled echo; MPRAGE = magnetization prepared rapid gradient echo; FSPGR BRAVO = fast spoiled gradient echo brain volume; 3D-FSE = 3D fast spin-echo.

Table S4. Cohort-level clinical characteristics for PTSD severity, depression severity, and childhood trauma for the patient group.

	Total Patients,	PTSD Severity				Depression Severity				Childhood Trauma			
Cohort	*N*	Instrument	N[1]	Mean	SD	Instrument	N[1]	Mean	SD	Instrument	N[1]	Mean	SD
ADNIDOD 1	50	CAPS-4	50	58.44	14.61	GDS-15	50	4.24	3.15	*NA*	*NA*	*NA*	*NA*
ADNIDOD 2	17	CAPS-4	17	53.65	10.16	GDS-15	17	4.24	3.05	*NA*	*NA*	*NA*	*NA*
AMC	37	CAPS-4	37	67.84	13.93	HADS-D	36	10.89	4.25	ETI	37	5.73	4.78
Beijing	42	CAPS-4	42	42.50	10.43	CES-D	39	24.69	9.61	*NA*	*NA*	*NA*	*NA*
Columbia-3	53	CAPS-4	53	80.11	15.47	*NA*	*NA*	*NA*	*NA*	*NA*	*NA*	*NA*	*NA*
Columbia-6	25	CAPS-5	25	36.52	9.31	HAMD-17	25	14.32	6.03	CTQ	25	54.88	19.92
Duke 1	11	CAPS-4, CAPS-5[2]	11	40.46	18.30	BDI-II	10	22.1	11.70	*NA*	*NA*	*NA*	*NA*
Duke 2	15	CAPS-4, CAPS-5[2]	15	49.46	18.16	BDI-II	15	19.87	15.32	CTA	14	52.36	27.22
Duke 3	15	CAPS-4	15	64.20	16.83	BDI-II	14	18.64	9.38	*NA*	*NA*	*NA*	*NA*
Duke 4	36	CAPS-4	36	73.86	19.89	BDI-II	20	27.45	11.08	*NA*	*NA*	*NA*	*NA*
Emory	14	CAPS-4, MPSS[2]	14	44.49	10.11	BDI-II	12	19.25	8.81	CTQ	14	53.86	18.38
INTRuST 1	72	PCL-C	71	53.97	16.54	PHQ-9	71	11.44	6.72	CTQ	71	49.58	20.72
INTRuST 2	31	PCL-C	17	52.71	14.40	PHQ-9	29	11.62	6.48	CTQ	24	55.92	23.70
Leiden	21	TSCC PTSD Subscale	18	12.22	6.61	CDI	18	15.67	6.59	*NA*	*NA*	*NA*	*NA*
LIMBIC-CENC 1	84	PCL-5	84	49.31	10.22	PHQ-9	82	13.71	5.34	*NA*	*NA*	*NA*	*NA*
LIMBIC-CENC 2	76	PCL-5	76	51.21	11.15	PHQ-9	76	14.13	4.84	*NA*	*NA*	*NA*	*NA*
LIMBIC-CENC 3	81	PCL-5	81	46.49	10.18	PHQ-9	81	12.96	4.24	*NA*	*NA*	*NA*	*NA*
McLean 1	50	CAPS-5	50	51.36	11.37	*NA*	*NA*	*NA*	*NA*	CTQ	41	79.44	20.93
McLean 2	22	CAPS-4	22	59.36	18.38	*NA*	*NA*	*NA*	*NA*	CTQ	21	60.67	22.84
Minnesota	12	CAPS-4	12	53.42	11.21	BDI-II	12	19.99	6.47	*NA*	*NA*	*NA*	*NA*
Münster	21	PDS	18	23.44	10.31	BDI-II	21	19.52	10.73	*NA*	*NA*	*NA*	*NA*
South Dakota	78	PCL-M, PCL-C[3]	78	47.24	13.08	CES-D, BDI-II[2]	78	28.36	18.84	*NA*	*NA*	*NA*	*NA*
Stanford	30	CAPS-4	29	59.52	18.87	*NA*	*NA*	*NA*		CTQ	27	72.67	24.71
Toledo	15	CAPS-4	11	63.91	15.73	CES-D, DASS-21[2]	14	32.16	25.06	CTQ	14	56.14	18.09
UMC BETTER	55	CAPS-4	55	70.69	13.23	MASQ Depressive Symptoms Subscale	49	28.9	8.75	ETI	48	5	4.67
VA Minn DEFEND	27	CAPS-4	27	65.11	24.11	BDI-II	22	20.95	9.80	*NA*	*NA*	*NA*	*NA*
VA Minn SATURN	55	CAPS-4	55	62.64	17.82	*NA*	*NA*	*NA*	*NA*	*NA*	*NA*	*NA*	*NA*
VA Waco	59	PCL-5	59	56.05	11.64	BDI-II	34	25.44	12.26	CTQ	26	61.04	24.14
VA West Haven	35	CAPS-4	35	67.89	15.56	BDI-II	35	25.46	10.29	*NA*	*NA*	*NA*	*NA*
Vanderbilt	15	CAPS-5	15	26.93	4.54	BDI-II	15	16.2	7.10	CTQ	15	37.87	11.62

Table S4. Cohort-level clinical characteristics for PTSD severity, depression severity, and childhood trauma for the patient group (*continued)*.

	Total Patients,	PTSD Severity				Depression Severity				Childhood Trauma			
Cohort	*N*	Instrument	N[1]	Mean	SD	Instrument	N[1]	Mean	SD	Instrument	N[1]	Mean	SD
Washington	33	CAPS-5	33	14.42	3.46	CDI	33	25.55	2.61	CTQ	32	47.47	17.58
Western Ontario	59	CAPS-4, CAPS-5[2]	59	51.38	10.45	BDI-II	52	26.5	10.63	CTQ	55	60.65	23.54
Wisconsin-Madison	19	CAPS-4	19	64.95	14.78	BDI-II	19	22.12	13.52	*NA*	*NA*	*NA*	*NA*
Wisconsin-Milwaukee	22	CAPS-5	22	28.64	7.40	DASS-21	22	16.18	10.81	CTQ	22	50.05	20.01
Yale	22	CAPS-4	22	50.05	24.13	BDI-II	22	21.64	11.79	CTQ	21	46.9	15.48

The descriptive statistics are reported based on the raw scores for each scale.

[1] N represents the number of patients with available clinical covariate data.

[2] Where cohorts used different scales for current PTSD severity or depression severity, participant scores were calculated as a percentage value of the maximum possible score depending on the scale used.

[3] Note, PCL-M and PCL-C were not converted to a percentage as both scales have the same maximum score.

PTSD Severity Instruments: CAPS-4/-5 = Clinician-Administered PTSD Scale for DSM-4 or DSM-5 [8, 9]; PCL-5/C/M = PTSD Checklist for DSM-5 (Civilian, or Military version) [10]; ADIS-C = Anxiety Disorders Interview Schedule for Children [11]; SCID = Structured Clinical Interview for DSM [12]; MINI = Mini International Neuropsychiatric Interview [13]; MPSS = Modified PTSD Symptom Scale [14]; TSCC = Trauma Symptom Checklist for Children [15]; PDS = Posttraumatic Stress Diagnostic Scale [16].

Depression Severity Instruments: GDS-15 = Geriatric Depression Scale-15 [17]; HADS-D = Hospital Anxiety and Depression Scale [18]; BDI-II = Beck Depression Inventory-II [19]; PHQ-9 = Patient Health Questionnaire-9 [20]; CDI = Children's Depression Inventory [21]; CES-D = Center for Epidemiological Studies Depression Scale [22]; DASS-21 = Depression Anxiety Stress Scale-21 [23]; MASQ = Mood and Anxiety Symptom Questionnaire [24].

Childhood Trauma Instruments: CTQ = Childhood Trauma Questionnaire [25]; ETI = Early Trauma Inventory [26].

Table S5. Cohort-level clinical characteristics for alcohol use disorder, drug use disorder, and antidepressant medication use for the patient group.

Cohort	Total Patients, *N*	Alcohol Use Disorder (AUD)				Drug Use Disorder (DUD)				Antidepressant Medication (AD)		
		Instrument	N[1]	N_{AUD}	%	Instrument	N[1]	N_{DUD}	%	N[1]	N_{AD}	%
ADNIDOD 1	50	SCID	50	19	38	SCID	26	2	7.7	*NA*	*NA*	*NA*
ADNIDOD 2	17	SCID	17	8	47.1	*NA*	*NA*	*NA*	*NA*	*NA*	*NA*	*NA*
AMC	37	AUDIT	37	4	10.8	*NA*	*NA*	*NA*	*NA*	*NA*	*NA*	*NA*
Beijing	42	*NA*	*NA*	*NA*	*NA*	*NA*	*NA*	*NA*	*NA*	*NA*	*NA*	*NA*
Columbia-3	53	*NA*	*NA*	*NA*	*NA*	*NA*	*NA*	*NA*	*NA*	*NA*	*NA*	*NA*
Columbia-6	25	*NA*	*NA*	*NA*	*NA*	*NA*	*NA*	*NA*	*NA*	*NA*	*NA*	*NA*
Duke 1	11	*NA*	*NA*	*NA*	*NA*	DAST		Naïve		11	4	36.4
Duke 2	15	*NA*	*NA*	*NA*	*NA*	DAST	15	1	6.7	15	7	46.7
Duke 3	15	*NA*	*NA*	*NA*	*NA*	DAST	14	1	7.1	15	9	60.0
Duke 4	36	*NA*	*NA*	*NA*	*NA*	*NA*	*NA*	*NA*	*NA*	36	8	22.2
Emory	14	AUDIT	10	4	40	*NA*	*NA*	*NA*	*NA*	14	2	14.3
INTRuST 1	72	AUDIT	72	41	56.9	DAST	72	6	8.3	*NA*	*NA*	*NA*
INTRuST 2	31	AUDIT	21	13	61.9	DAST	31	8	25.8	*NA*	*NA*	*NA*
Leiden	21	*NA*	*NA*	*NA*	*NA*	*NA*	*NA*	*NA*	*NA*	*NA*	*NA*	*NA*
LIMBIC-CENC 1	84	AUDIT	83	1	1.2	DAST		Naïve		*NA*	*NA*	*NA*
LIMBIC-CENC 2	76	AUDIT	76	9	11.8	DAST	76	7	9.2	*NA*	*NA*	*NA*
LIMBIC-CENC 3	81	AUDIT	81	3	3.7	DAST	81	1	1.2	*NA*	*NA*	*NA*
McLean 1	50	*NA*	*NA*	*NA*	*NA*	*NA*	*NA*	*NA*	*NA*	46	34	73.9
McLean 2	22	*NA*	*NA*	*NA*	*NA*	*NA*	*NA*	*NA*	*NA*	22	2	9.1
Minnesota	12	SCID		Naïve		SCID		Naïve		12	4	33.3
Münster	21	*NA*	*NA*	*NA*	*NA*	*NA*	*NA*	*NA*	*NA*	18	4	22.2
South Dakota	78	AUDIT	78	45	57.7	*NA*	*NA*	*NA*	*NA*	78	11	14.1
Stanford	30	*NA*	*NA*	*NA*		*NA*	*NA*	*NA*	*NA*	*NA*	*NA*	*NA*
Toledo	15	MINI	15	2	13.3	MINI	15	2	13.3	*NA*	*NA*	*NA*
UMC BETTER	55	*NA*	*NA*	*NA*	*NA*	SCID	55	8	14.5	*NA*	*NA*	*NA*
VA Minn DEFEND	27	AUDIT	17	1	5.9	*NA*	*NA*	*NA*	*NA*	*NA*	*NA*	*NA*
VA Minn SATURN	55	AUDIT	50	13	26	*NA*	*NA*	*NA*	*NA*	*NA*	*NA*	*NA*
VA Waco	59	AUDIT	34	6	17.6	*NA*	*NA*	*NA*	*NA*	59	19	32.3
VA West Haven	35	SCID	20	4	20	SCID	20	3	15	*NA*	*NA*	*NA*
Vanderbilt	15	*NA*	*NA*	*NA*	*NA*	*NA*	*NA*	*NA*	*NA*		Naïve	

Table S5. Cohort-level clinical characteristics for alcohol use disorder, drug use disorder, and antidepressant medication use for the patient group *(continued)*.

	Total Patients,	Alcohol Use Disorder (AUD)				Drug Use Disorder (DUD)				Antidepressant Medication (AD)		
**Cohort**	***N***	**Instrument**	**N[1]**	**N_{AUD}**	**%**	**Instrument**	**N[1]**	**N_{DUD}**	**%**	**N[1]**	**N_{AD}**	**%**
Washington	33	*NA*	*NA*	*NA*	*NA*	*NA*	*NA*	*NA*	*NA*	*NA*	*NA*	*NA*
Western Ontario	59	*NA*	*NA*	*NA*	*NA*	*NA*	*NA*	*NA*	*NA*	*NA*	*NA*	*NA*
Wisconsin-Madison	19	AUDIT	19	1	5.3	SCID		Naïve		19	7	36.8
Wisconsin-Milwaukee	22	*NA*	*NA*	*NA*	*NA*	*NA*	*NA*	*NA*	*NA*	19	1	5.3
Yale	22	*NA*	*NA*	*NA*	*NA*	*NA*	*NA*	*NA*	*NA*	*NA*	*NA*	*NA*
TOTAL	***1309***		***680***	***174***	***25.6***		***405***	***39***	9.6	***364***	***112***	30.8

[1] N represents the number of patients with available clinical covariate data.

Alcohol Use Disorder Instruments: SCID = Structured Clinical Interview for DSM [12]; MINI = Mini International Neuropsychiatric Interview [13]; AUDIT = Alcohol Use Disorder Identification Test [3]

Drug Use Disorder Instruments: SCID = Structured Clinical Interview for DSM [12]; MINI = Mini International Neuropsychiatric Interview [13]; DAST = Drug Abuse Screening Test [4]

Supplementary Results

Summary Results Table. Summary table of all voxel-based analysis presenting the peak coordinate with the largest effect and a snapshot of the axial slice at the peak coordinate. Highlighted regions represent significant clusters – in group comparisons, orange represents where cases < controls and blue where cases > controls. In regression analyses, blue represents where the clinical variable is negatively associated with brain volume. Detailed results for each comparison are provided in the following sections. In addition, 3D maps are available online at Neurovault: https://neurovault.org/collections/QOAYXFZK.

	Sample Size			GREY MATTER			WHITE MATTER		
**Analysis**	**PTSD**	**Controls**	**No. Cohorts**	**Peak Coordinate**	**Hedges' *g***	***p*-value**	**Peak Coordinate**	**Hedges' *g***	***p*-value**
Group Comparison									
PTSD vs. All Controls	1309	2130	35	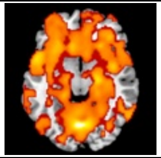 Left cerebellum [-4,-72,-10]	0.22	.001	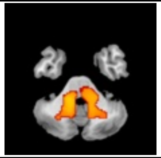 Middle cerebellar peduncle [-16, -54, -38]	0.14	.008
PTSD vs. TE Controls	912	1342	28	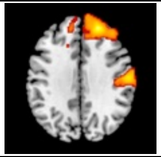 Right superior frontal gyrus [22,46,34]	0.20	.001	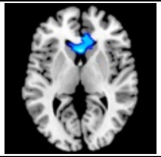 Corpus callosum [0,18,4]	-0.16	.007
Military Cohorts PTSD vs. All Controls	697	1148	19	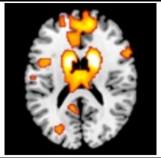 Right caudate [12,8,18]	0.24	.001	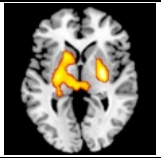 WM adjacent to left striatum [-28,-14,0]	0.20	.002
Civilian Cohorts PTSD vs. All Controls	412	614	13	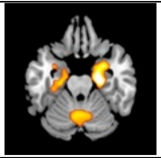 Right parahippocampus [24,-18,-24]	0.30	.001	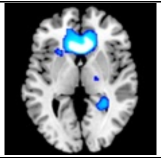 Corpus callosum [2,18,2]	-0.31	.001

Summary Results Table. (*continued*)

Analysis	Sample Size		No. Cohorts	GREY MATTER			WHITE MATTER		
	PTSD	Controls		Peak Coordinate	Hedges' *g*	*p*-value	Peak Coordinate	Hedges' *g*	*p*-value
Regression Analysis (within PTSD only)									
PTSD severity	1283	*NA*	35	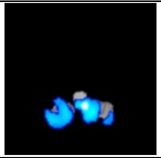 Right cerebellum [4,-48,-58]	-0.11	.003	*No significant associations*		
Depression severity	1023	*NA*	30	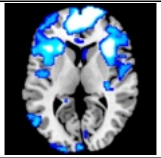 Right superior frontal gyrus [14,66,6]	-0.15	.001	*No significant associations*		
Alcohol use disorder	680	*NA*	16	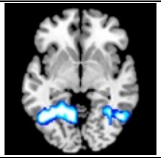 Left fusiform gyrus [-34,-56,-6]	-0.15	.003	*No significant associations*		
Antidepressant medication use	364	*NA*	13	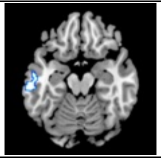 Left inferior temporal gyrus [-60,-26,-18]	-0.17	.017	*No significant associations*		
Drug use disorder	405	*NA*	10	*No significant associations*			*No significant associations*		
Childhood trauma	507	*NA*	17	*No significant associations*			*No significant associations*		
Sensitivity Analysis (PTSD vs. All Controls)									
Excluding non-adult cohorts	1255	1984	33	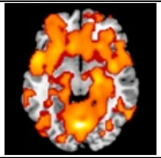 Left cerebellum [-4,-72,-10]	0.22	.001	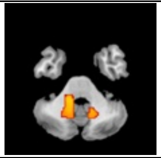 Left cerebellum [-14,-56,-38]	0.13	.012

Summary Results Table. (*continued*)

	Sample Size			GREY MATTER			WHITE MATTER		
**Analysis**	**PTSD**	**Controls**	**No. Cohorts**	**Peak Coordinate**	**Hedges' *g***	***p*-value**	**Peak Coordinate**	**Hedges' *g***	***p*-value**
Sensitivity Analysis (PTSD vs. All Controls)									
Excluding traumatic brain injury	927	1603	33	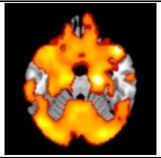 Right parahippocampus [22,-18,-24]	0.23	.001	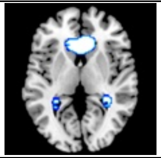 Corpus callosum [-2,18,4]	-0.18	.002
Covarying age, ICV, sex	1228	2025	32	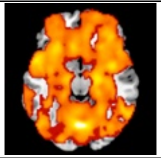 Left cerebellum [-4,-72,-12]	0.22	.001	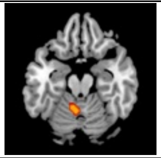 Left cerebellum [-6,-54,-20]	0.14	.020
Covarying age and total GM or total WM	1309	2130	35	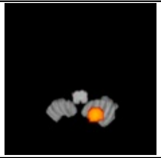 Right cerebellum [16,-58,-56]	0.14	.009	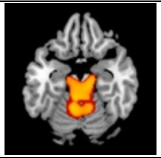 Left cerebellum [-16,-54,-38]	0.16	.001
Covarying age, age^2, ICV, sex	1228	2025	32	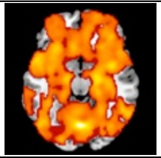 Left cerebellum [-4,-72,-12]	0.21	.001	*No significant differences*		
Covarying age, sex	1228	2025	32	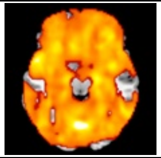 Left cerebellum [-4,-72,-12]	0.24	< .001	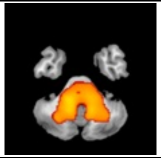 Middle cerebellar peduncles [-16,-54,-38]	0.15	< .001
Covarying ICV	1309	2130	35	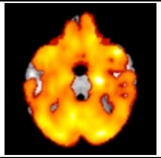 Right parahippocampus [24,-18,-24]	0.22	.001	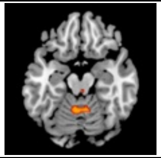 Cerebellum vermis [-2,-54,-18]	0.13	.017

Summary Results Table. (*continued*)

	Sample Size			GREY MATTER				WHITE MATTER			
**Analysis**	**PTSD**	**Controls**	**No. Cohorts**	**Peak Coordinate**		**Hedges' *g***	***p*-value**	**Peak Coordinate**		**Hedges' *g***	***p*-value**
Proportional scaling, covarying age and ICV	1309	2130	35	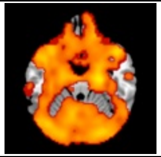	Right fusiform gyrus [28,2,-50]	0.22	.001	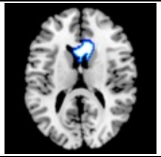	Corpus callosum [8,16,14]	-0.14	.006
No covariates	1309	2130	35	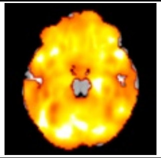	Right parahippocampus [18,-22,-18]	0.22	.001	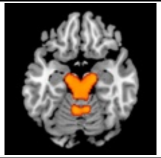	Left cerebellum [-16,-54,-38]	0.14	.002
Non-modulated data	1309	2130	35	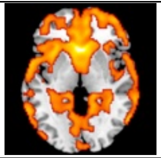	Left olfactory [-4,22,0]	0.21	< .001	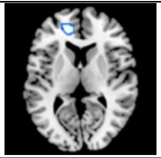	Left median cingulum [-16,46,8]	-0.16	.007
Non-modulated data, no covariates	1309	2130	35	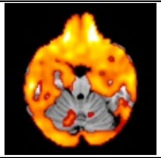	Left frontal superior gyrus [-16,38,-22]	0.22	.001	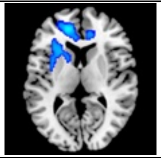	Left median cingulum [-14,44,8]	-0.18	.002
Smoothing 2mm	1309	2130	35	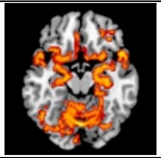	Cerebellum vermis [0,-70,-16]	0.21	.001	*No significant differences*			
Smoothing 4mm	1309	2130	35	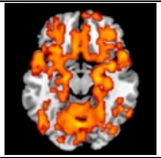	Cerebellum vermis [-2,-72,-14]	0.20	.001	*No significant differences*			
Smoothing 12mm	1309	2130	35	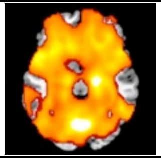	Cerebellum vermis [-2,-70,-12]	0.22	.001	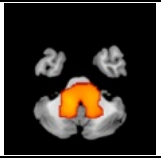	Left cerebellum [-16,-56,-40]	0.13	.003

Group comparisons were adjusted for age and ICV unless specified; regression analyses were adjusted for age, ICV, and sex.
All p-values have been FWE-corrected for multiple comparisons.

Group Comparisons

All group comparison analyses were adjusted for age and ICV.

Table S6. PTSD vs. Controls

Peak Regions	MNI coordinate	Hedges' g	Z	Cluster size (voxels)	*p*-value[a]	I^2
**GREY MATTER** (Mean I^2 = 8.15 (all voxels in the brain))						
Large cluster comprising regions across the frontal lobe, temporal lobe, thalamus, and cerebellum.				84,883		
Left cerebellum	-4,-72,-10	0.22	5.978	Subcluster	.001	0.00
Right parahippocampus	22,-18,-24	0.20	5.509	Subcluster	.001	0.00
Right fusiform gyrus	28,0,-50	0.19	5.277	Subcluster	.001	0.00
Left fusiform gyrus	-34,-18,-34	0.19	5.151	Subcluster	.001	0.93
Left fusiform gyrus	-30,2,-44	0.19	5.136	Subcluster	.001	2.49
**WHITE MATTER** (Mean I^2 = 4.67 (all voxels in the brain))						
Cluster across the cerebellum.				2,423		
Middle cerebellar peduncles	-16,-54,-38	0.14	3.612	Subcluster	.008	5.24
Left cerebellum	-6,-54,-18	0.14	3.470	Subcluster	.009	14.32
Middle cerebellar peduncles	-14,-40,-42	0.16	3.376	Subcluster	.008	33.67
Middle cerebellar peduncles	14,-58,-40	0.12	3.352	Subcluster	.012	0.00
Left cerebellum	-12,-52,-20	0.13	3.340	Subcluster	.009	11.47

[a]p-values have been FWE-corrected for multiple comparisons
Data included in the analysis comprised 1,309 PTSD patients and 2,130 controls from 35 cohorts.

Figure S1. PTSD vs. Controls – differences in WM volumes
White Matter: patients exhibited smaller WM volumes compared to controls.

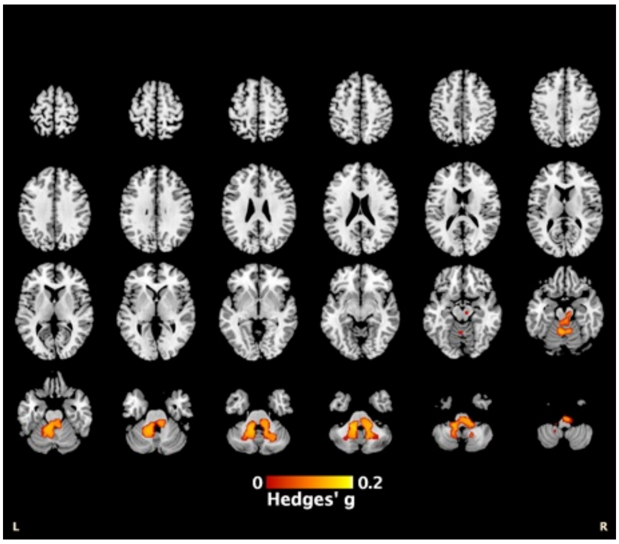

Note: For GM volumes, see Figure 1 in the main paper.

Figure S2. Forest plot comparing total GM volumes between PTSD patients and controls

Study	PTSD Patients N	PTSD Patients Mean	PTSD Patients SD	Controls N	Controls Mean	Controls SD	Hedges's g with 95% CI	Weight (%)
ADNIDOD 1	50	.669	.071	61	.649	.063	0.29 [-0.09, 0.66]	3.44
ADNIDOD 2	17	.659	.055	31	.662	.064	-0.05 [-0.63, 0.53]	2.08
AMC	37	.739	.07	37	.741	.059	-0.04 [-0.49, 0.41]	2.85
Beijing	42	.643	.055	46	.683	.068	-0.63 [-1.06, -0.21]	3.03
Columbia-3	53	.636	.071	36	.65	.061	-0.21 [-0.63, 0.21]	3.06
Columbia-6	25	.679	.071	55	.678	.063	0.01 [-0.46, 0.48]	2.73
Duke 1	11	.753	.072	73	.71	.057	0.71 [0.07, 1.34]	1.84
Duke 2	15	.684	.067	33	.704	.071	-0.29 [-0.89, 0.31]	1.98
Duke 3	15	.697	.084	31	.735	.084	-0.44 [-1.06, 0.17]	1.94
Duke 4	36	.694	.078	75	.72	.072	-0.35 [-0.75, 0.05]	3.25
Emory	14	.604	.076	48	.658	.089	-0.62 [-1.22, -0.02]	2.01
INTRuST 1	72	.711	.072	147	.726	.075	-0.20 [-0.48, 0.08]	4.28
INTRuST 2	31	.652	.079	94	.679	.072	-0.36 [-0.77, 0.04]	3.18
Leiden	21	.757	.082	30	.783	.068	-0.36 [-0.91, 0.20]	2.23
LIMBIC-CENC 1	84	.679	.075	179	.697	.076	-0.24 [-0.50, 0.02]	4.51
LIMBIC-CENC 2	76	.709	.062	84	.726	.068	-0.26 [-0.57, 0.05]	4.01
LIMBIC-CENC 3	81	.792	.151	144	.78	.1	0.10 [-0.17, 0.37]	4.38
McLean 1	50	.658	.058	26	.651	.053	0.13 [-0.34, 0.60]	2.72
McLean 2	22	.85	.075	74	.931	.108	-0.79 [-1.28, -0.31]	2.62
Minnesota	12	.756	.06	50	.74	.08	0.19 [-0.43, 0.82]	1.90
Muenster	21	.732	.07	26	.765	.063	-0.49 [-1.06, 0.09]	2.12
South Dakota	78	.759	.069	44	.765	.065	-0.09 [-0.46, 0.28]	3.49
Stanford	30	.679	.067	50	.709	.075	-0.41 [-0.86, 0.04]	2.83
Toledo	15	.675	.057	63	.727	.078	-0.69 [-1.26, -0.13]	2.15
UMC BETTER	55	.729	.078	52	.745	.054	-0.24 [-0.61, 0.14]	3.41
VA Minn DEFEND	27	.725	.048	82	.736	.056	-0.20 [-0.63, 0.23]	2.98
VA Minn SATURN	55	.755	.068	62	.729	.054	0.42 [0.06, 0.78]	3.52
VA Waco	59	.441	.143	31	.497	.158	-0.37 [-0.81, 0.06]	2.96
VA West Haven	35	.716	.104	30	.741	.098	-0.25 [-0.73, 0.24]	2.63
Vanderbilt	15	.742	.055	35	.726	.064	0.25 [-0.35, 0.85]	2.01
Washington	33	.797	.07	116	.795	.076	0.02 [-0.36, 0.41]	3.35
Western Ontario	59	.66	.058	39	.68	.054	-0.35 [-0.75, 0.06]	3.19
Wisconsin-Madison	19	.743	.076	38	.785	.058	-0.65 [-1.21, -0.10]	2.21
Wisconsin-Milwaukee	22	.66	.076	60	.639	.074	0.28 [-0.21, 0.76]	2.62
Yale	22	.719	.06	48	.756	.084	-0.48 [-0.98, 0.03]	2.50
**Overall**							-0.18 [-0.29, -0.08]	

Heterogeneity: $I^2 = 47.9\%$

$z = -3.49$, $p = .001$

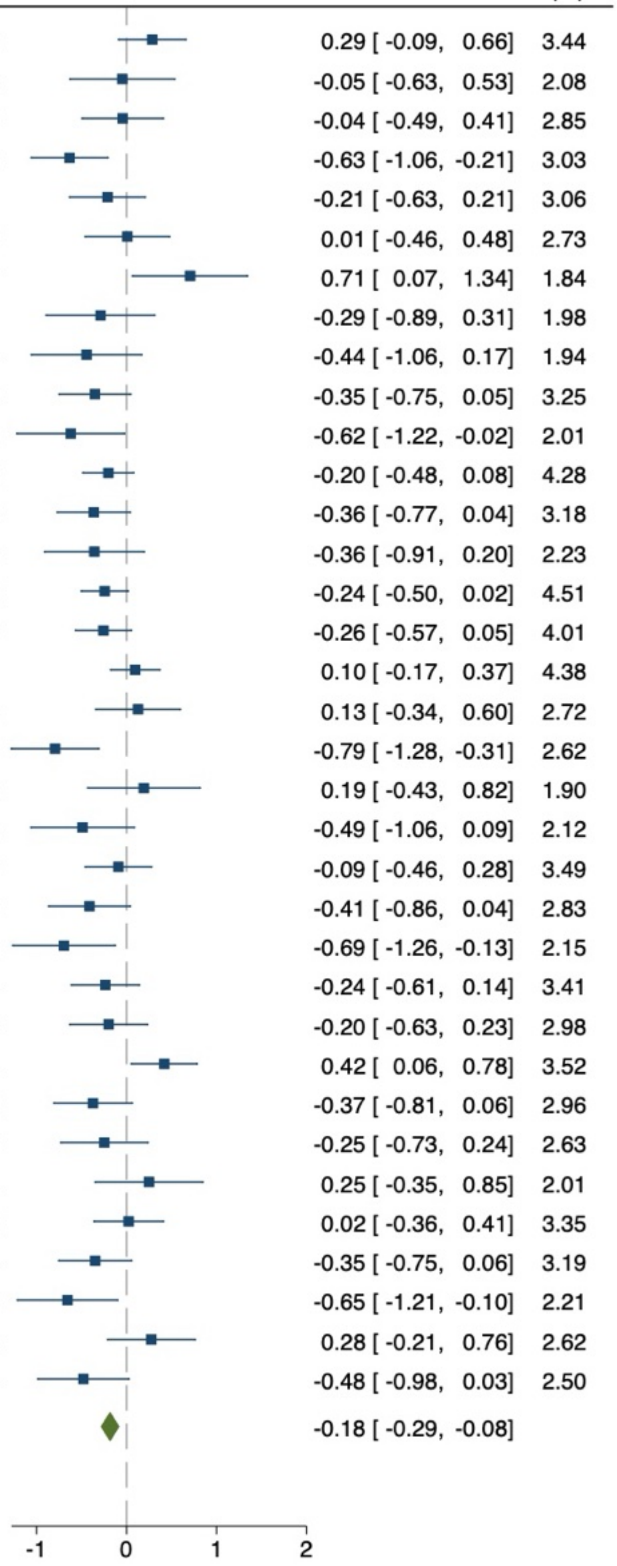

Data included in the analysis comprised 1,309 PTSD patients and 2,130 controls from 35 cohorts.

Figure S3. Forest plot comparing total WM volumes between PTSD patients and controls

Study	PTSD Patients N	PTSD Patients Mean	PTSD Patients SD	Controls N	Controls Mean	Controls SD	Hedges's g with 95% CI	Weight (%)
ADNIDOD 1	50	.419	.046	61	.422	.046	-0.06 [-0.43, 0.31]	3.69
ADNIDOD 2	17	.411	.048	31	.434	.056	-0.43 [-1.01, 0.16]	1.49
AMC	37	.453	.057	37	.445	.052	0.15 [-0.30, 0.60]	2.52
Beijing	42	.439	.045	46	.442	.057	-0.06 [-0.48, 0.35]	2.98
Columbia-3	53	.478	.076	36	.473	.06	0.07 [-0.35, 0.49]	2.91
Columbia-6	25	.461	.032	55	.442	.054	0.40 [-0.07, 0.88]	2.30
Duke 1	11	.48	.066	73	.468	.053	0.23 [-0.40, 0.85]	1.31
Duke 2	15	.482	.052	33	.485	.051	-0.06 [-0.66, 0.54]	1.43
Duke 3	15	.471	.038	31	.463	.058	0.16 [-0.45, 0.77]	1.40
Duke 4	36	.464	.053	75	.47	.051	-0.11 [-0.51, 0.28]	3.28
Emory	14	.383	.052	48	.372	.041	0.24 [-0.35, 0.83]	1.49
INTRuST 1	72	.449	.047	147	.444	.052	0.08 [-0.20, 0.36]	6.34
INTRuST 2	31	.475	.07	94	.457	.054	0.31 [-0.10, 0.71]	3.11
Leiden	21	.395	.049	30	.401	.04	-0.12 [-0.67, 0.43]	1.71
LIMBIC-CENC 1	84	.434	.056	179	.439	.046	-0.11 [-0.37, 0.15]	7.43
LIMBIC-CENC 2	76	.449	.057	84	.442	.044	0.13 [-0.18, 0.44]	5.28
LIMBIC-CENC 3	81	.416	.054	144	.426	.05	-0.19 [-0.46, 0.08]	6.76
McLean 1	50	.425	.039	26	.43	.032	-0.14 [-0.61, 0.33]	2.33
McLean 2	22	.256	.063	74	.252	.038	0.09 [-0.39, 0.56]	2.30
Minnesota	12	.486	.067	50	.476	.059	0.16 [-0.46, 0.79]	1.33
Muenster	21	.441	.044	26	.442	.044	-0.01 [-0.57, 0.56]	1.62
South Dakota	78	.521	.067	44	.52	.055	0.03 [-0.34, 0.39]	3.78
Stanford	30	.427	.056	50	.45	.06	-0.38 [-0.83, 0.07]	2.51
Toledo	15	.425	.039	63	.437	.062	-0.20 [-0.76, 0.36]	1.66
UMC BETTER	55	.487	.056	52	.482	.043	0.11 [-0.27, 0.48]	3.59
VA Minn DEFEND	27	.501	.058	82	.511	.056	-0.17 [-0.61, 0.26]	2.74
VA Minn SATURN	55	.489	.052	62	.48	.054	0.17 [-0.19, 0.53]	3.90
VA Waco	59	.336	.072	31	.366	.085	-0.38 [-0.82, 0.05]	2.71
VA West Haven	35	.469	.072	30	.511	.076	-0.56 [-1.05, -0.07]	2.13
Vanderbilt	15	.487	.058	35	.462	.051	0.45 [-0.15, 1.06]	1.43
Washington	33	.426	.054	116	.419	.044	0.14 [-0.24, 0.53]	3.44
Western Ontario	59	.454	.058	39	.475	.055	-0.38 [-0.79, 0.02]	3.12
Wisconsin-Madison	19	.449	.046	38	.463	.039	-0.33 [-0.88, 0.21]	1.73
Wisconsin-Milwaukee	22	.489	.052	60	.501	.065	-0.19 [-0.68, 0.29]	2.19
Yale	22	.434	.055	48	.45	.05	-0.30 [-0.80, 0.20]	2.05
**Overall**							-0.04 [-0.11, 0.04]	

Heterogeneity: $I^2 = 1.8\%$

$z = -1.01$, $p = .314$

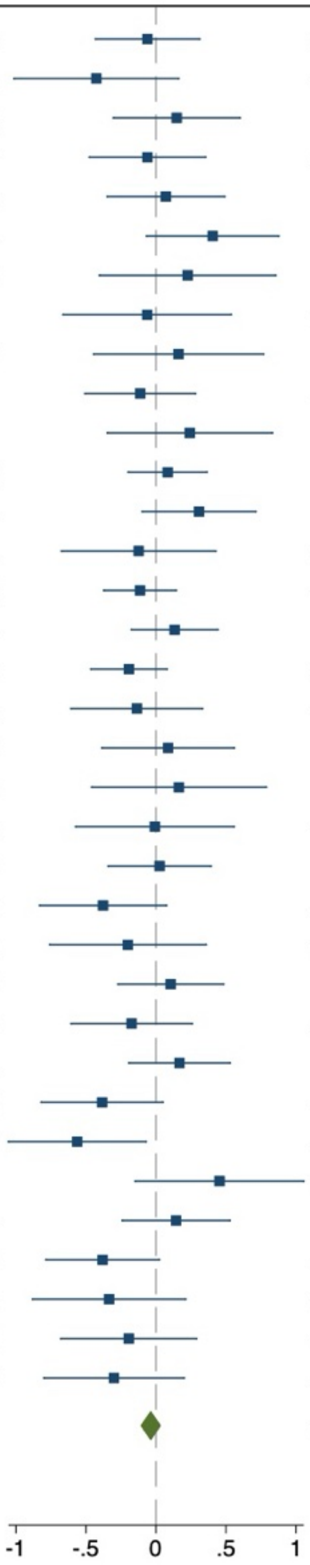

Data included in the analysis comprised 1,309 PTSD patients and 2,130 controls from 35 cohorts.

Table S7. PTSD vs. Trauma-Exposed Controls

Peak Regions	MNI coordinate	Hedges' g	Z	Cluster size (voxels)	*p*-value[a]	I^2
**GREY MATTER** (Mean I^2 = 9.32 (all voxels in the brain))						
Right superior frontal gyrus	22,46,34	0.20	4.384	32,799	.001	0.00
Right inferior parietal	56,-46,50	0.19	4.190	118	.004	0.00
Left precuneus	-10,-78,46	0.14	3.062	149	.016	0.00
Left caudate	-8,10,20	0.13	2.804	26	.021	0.00
Left precuneus	-2,-66,26	0.13	2.759	21	.021	0.00
**WHITE MATTER** (Mean I^2 = 1.13 (all voxels in the brain))						
Cluster within the corpus callosum.				1,671		
Corpus callosum	0,18,4	-0.16	-3.432	Subcluster	.007	0.00
Corpus callosum	0,12,14	-0.14	-3.176	Subcluster	.008	0.00
Corpus callosum	-2,8,16	-0.14	-3.163	Subcluster	.008	0.00
Corpus callosum	6,12,16	-0.14	-3.122	Subcluster	.008	0.00
Corpus callosum	6,16,14	-0.14	-3.115	Subcluster	.008	0.00

[a]p-values have been FWE-corrected for multiple comparisons
Data included in the analysis comprised 912 PTSD patients and 1,342 TE controls from 28 cohorts.

Figure S4. PTSD vs. Trauma-Exposed Controls - differences in GM and WM
(A) **Grey Matter**: Patients exhibited smaller GM volumes than controls.
(B) **White Matter**: Patients exhibited greater WM volumes than controls.

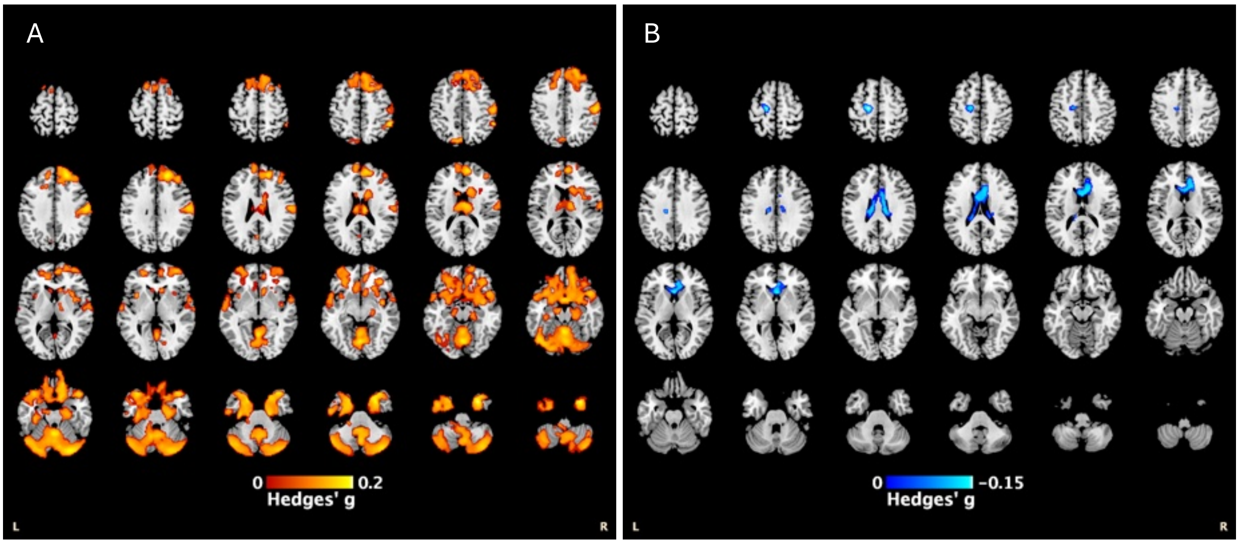

Table S8. Military-recruited cohorts

Peak Regions	MNI coordinate	Hedges' g	Z	Cluster size (voxels)	*p*-value[a]	I^2
**GREY MATTER** (Mean I^2 = 8.90 (all voxels in the brain))						
Right caudate	12,8,18	0.24	4.895	45,271	.001	0.00
Left postcentral gyrus	-64,-18,30	0.16	3.191	595	.011	0.00
Left Rolandic operculum	-44,6,16	0.15	2.914	171	.015	0.00
**WHITE MATTER** (Mean I^2 = 4.85 (all voxels in the brain))						
WM adjacent to the left striatum	-28,-14,0	0.20	3.968	5,673	.002	0.00
WM adjacent to the right striatum	28,-10,-2	0.21	4.160	535	.003	0.00

[a]p-values have been FWE-corrected for multiple comparisons
Data included in the analysis comprised 697 PTSD patients and 1,148 controls from 19 cohorts.

Figure S5. Military-recruited cohorts – differences in GM and WM
(A) **Grey Matter:** Patients exhibited smaller GM compared to controls.
(B) **White Matter:** Patients exhibited smaller WM compared to controls.

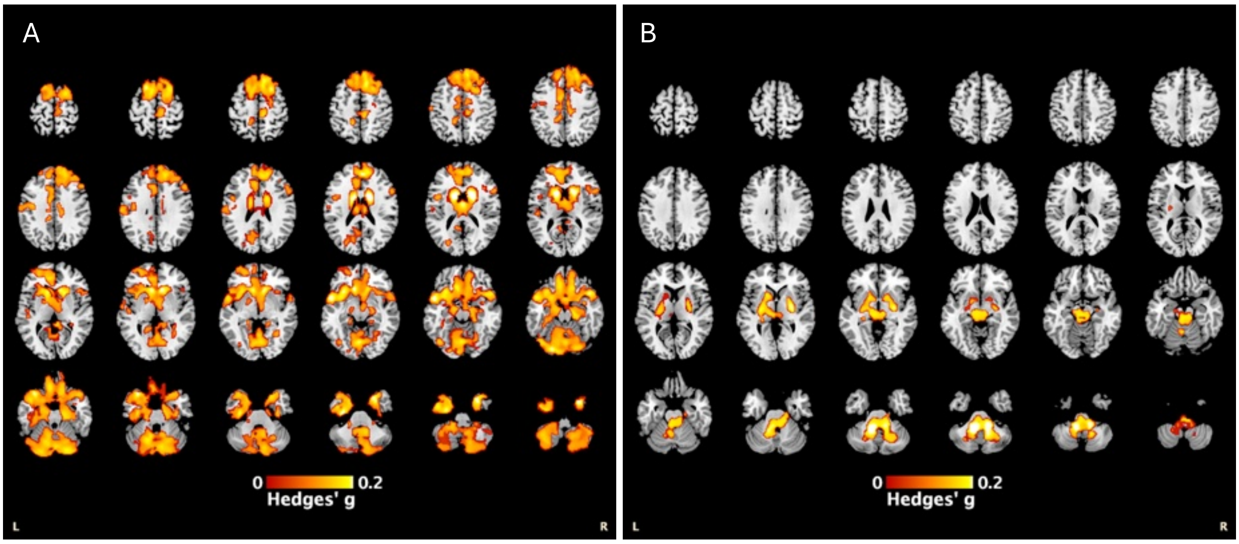

Table S9. Civilian-recruited cohorts

Peak Regions	MNI coordinate	Hedges' g	Z	Cluster size (voxels)	*p*-value[a]	I^2
**GREY MATTER** (Mean I^2 = 11.75 (all voxels in the brain))						
Right parahippocampus	24,-18,-24	0.30	4.368	4400	.001	0.00
Left parahippocampus	-20,4,-34	0.21	3.111	1092	.014	0.00
Left middle temporal gyrus	-66,-50,4	0.26	3.763	991	.005	0.00
Right cerebellum	42,-80,-38	0.26	3.887	644	.007	0.00
Right middle temporal gyrus	66,-48,-6	0.23	3.406	280	.014	0.00
**WHITE MATTER** (Mean I^2 = 4.16 (all voxels in the brain))						
Corpus callosum	2,18,2	-0.31	-4.503	11,745	.001	0.00
Corpus callosum	12,-2,50	-0.24	-3.590	147	.013	0.00
Corpus callosum	10,-18,56	-0.21	-3.040	32	.022	0.56
WM adjacent to the right precentral gyrus	24,-24,66	-0.22	-3.195	25	.022	0.00

[a]p-values have been FWE-corrected for multiple comparisons
Data included in the analysis comprised 412 PTSD patients and 614 controls from 13 cohorts.

Figure S6. Civilian-recruited cohorts – differences in GM and WM
(A) **Grey Matter**: Patients exhibited smaller GM volumes than controls.
(B) **White Matter**: Patients exhibited greater WM volumes than controls.

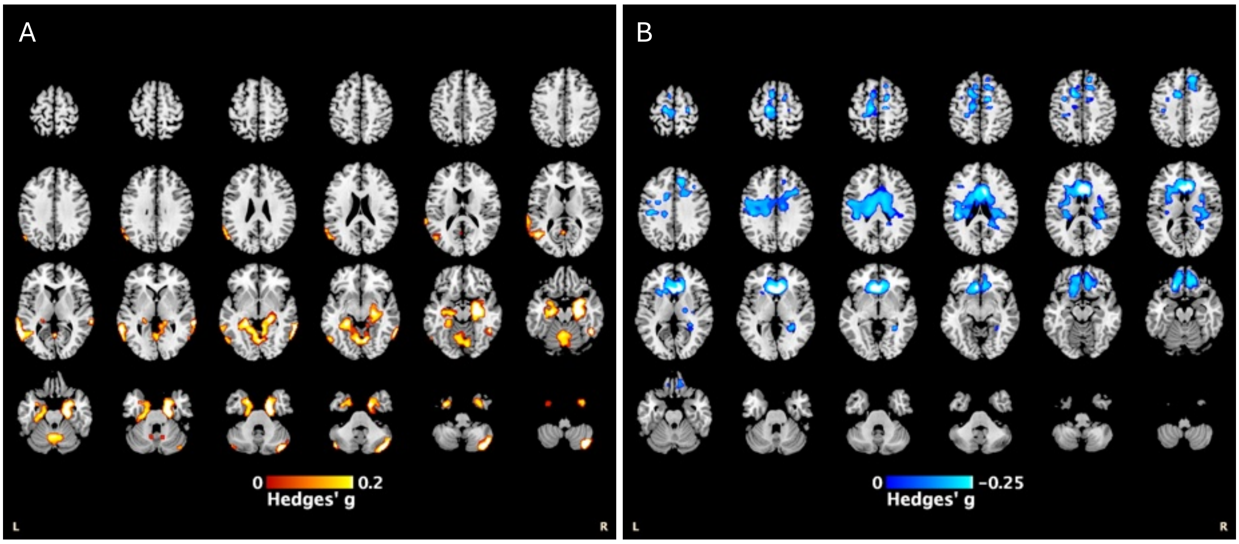

Regression Analyses of Clinical Variables

Regression analyses of the clinical variables is performed within the patient group only, investigating associations between brain volumes and clinical variables. All analyses were adjusted for age, ICV, and sex.

Table S10. PTSD severity associations with brain volume

Peak Regions	MNI coordinate	Hedges' g	Z	Cluster size (voxels)	*p*-value[a]	I^2
**GREY MATTER** (Mean I^2 = 5.93 (all voxels in the brain))						
Right cerebellum	4,-48,-58	-0.11	-4.014	4089	.003	0.00
Left cerebellum	-4,-60,-12	-0.09	-3.444	2674	.007	0.00
Right lingual gyrus	6,-86,-8	-0.10	-3.439	1073	.007	0.00
Left lingual gyrus	-26,-58,-4	-0.11	-3.977	658	.005	0.00
Right superior frontal gyrus	12,68,6	-0.10	-3.633	138	.017	0.00
**WHITE MATTER**						
No significant associations.						

[a]p-values have been FWE-corrected for multiple comparisons
Data included in the analysis comprised 1,283 PTSD patients from 35 cohorts.
See also Fig. 2A in the main paper.

Table S11. Depression severity associations with brain volume

Peak Regions	MNI coordinate	Hedges' g	Z	Cluster size (voxels)	*p*-value[a]	I^2
**GREY MATTER** (Mean I^2 = 6.54 (all voxels in the brain))						
Right superior frontal gyrus	14,66,6	-0.15	-4.850	31971	.001	0.00
Left cerebellum	-22,-86,-38	-0.13	-4.054	2552	.003	0.52
Left cerebellum	-16,-42,-30	-0.09	-2.845	2068	.010	0.00
Left middle temporal gyrus	-60,-64,20	-0.12	-3.919	1206	.007	0.00
Right lingual gyrus	8,-84,-4	-0.11	-3.424	720	.010	0.00
**WHITE MATTER**						
No significant associations.						

[a]p-values have been FWE-corrected for multiple comparisons
Data included in the analysis comprised 1,023 PTSD patients from 30 cohorts.
See also Fig. 2B in the main paper.

Table S12. Alcohol use disorder associations with brain volume

Peak Regions	MNI coordinate	Hedges' g	Z	Cluster size (voxels)	*p*-value[a]	I^2
**GREY MATTER** (Mean I^2 = 6.31 (all voxels in the brain))						
Cluster across the cerebellum and temporal lobe.				12,041		
Left fusiform gyrus	-34,-56,-6	-0.15	-3.801	Subcluster	.003	0.00
Right cerebellum	30,-70,-34	-0.15	-3.772	Subcluster	.002	0.00
Right cerebellum	30,-70,-42	-0.14	-3.589	Subcluster	.002	0.00
Right cerebellum	32,-66,-44	-0.14	-3.508	Subcluster	.002	0.00
Right cerebellum	34,-60,-46	-0.13	-3.476	Subcluster	.002	0.00
**WHITE MATTER**						
No significant associations.						

[a]p-values have been FWE-corrected for multiple comparisons
Data included in the analysis comprised 680 PTSD patients from 16 cohorts.

Figure S7. Alcohol use disorder associations with brain volume – GM associations
Grey Matter: Patients with alcohol use disorder exhibited smaller GM volumes.

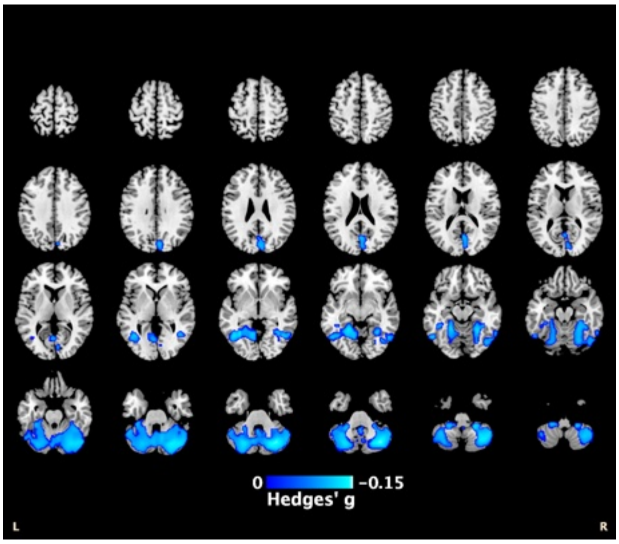

Table S13. Antidepressant medication use associations with brain volume

Peak Regions	MNI coordinate	Hedges' g	Z	Cluster size (voxels)	*p*-value[a]	I^2
**GREY MATTER** (Mean I^2 = 6.63 (all voxels in the brain))						
Cluster within the left temporal gyrus.				174		
Left inferior temporal gyrus	-60,-26,-18	-0.17	-3.150	Subcluster	.017	0.00
Left middle temporal gyrus	-56,-12,-22	-0.16	-2.967	Subcluster	.020	0.00
Left middle temporal gyrus	-54,-18,-16	-0.15	-2.832	Subcluster	.020	0.00
Left middle temporal gyrus	-58,-8,-16	-0.14	-2.640	Subcluster	.023	0.00
**WHITE MATTER**						
No significant associations.						

[a]p-values have been FWE-corrected for multiple comparisons
Data included in the analysis comprised 364 PTSD patients from 13 cohorts.

Figure S8. Antidepressant medication associations with brain volume – GM associations
Grey Matter: Patients on antidepressant medication exhibited smaller GM volumes.

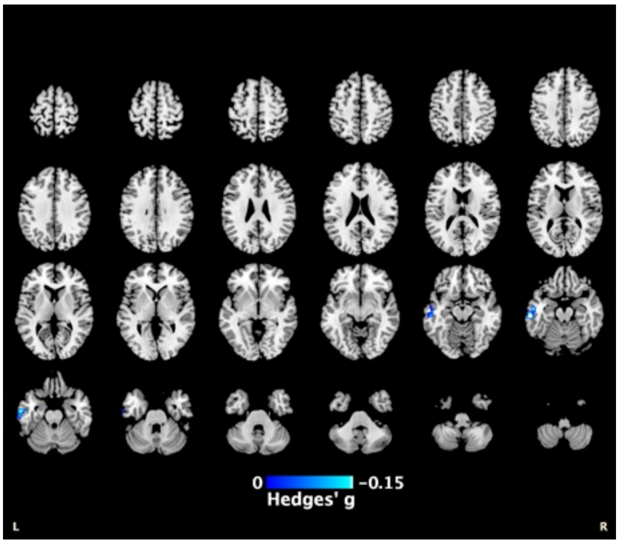

Sensitivity Analyses: exclusion of non-adults and adults with traumatic brain injury (TBI)

Table S14. PTSD vs. Controls excluding two non-adult cohorts

Peak Regions	MNI coordinate	Hedges' g	Z	Cluster size (voxels)	*p*-value[a]	I^2
**GREY MATTER** (Mean I^2 = 8.49 (all voxels in the brain))						
Left cerebellum	-4,-72,-10	0.22	5.792	80,940	.001	0.80
Right precuneus	12,-58,46	0.11	2.829	62	.024	0.00
**WHITE MATTER** (Mean I^2 = 4.99 (all voxels in the brain))						
Left cerebellum	-14,-56,-38	0.13	3.555	537	.012	0.87
Right cerebellum	14,-58,-40	0.13	3.480	70	.021	0.00

[a]p-values have been FWE-corrected for multiple comparisons
Data included in the analysis comprised 1,255 PTSD patients and 1,984 controls from 33 cohorts. See Table S28 for the results of the correlation analysis.

Figure S9. PTSD vs. Controls excluding two non-adult cohorts – differences in GM and WM
(A) **Grey Matter**: Patients exhibited similar results to the main case-control finding with smaller GM volumes than controls.
(B) **White Matter**: Patients exhibited smaller WM volumes than controls.

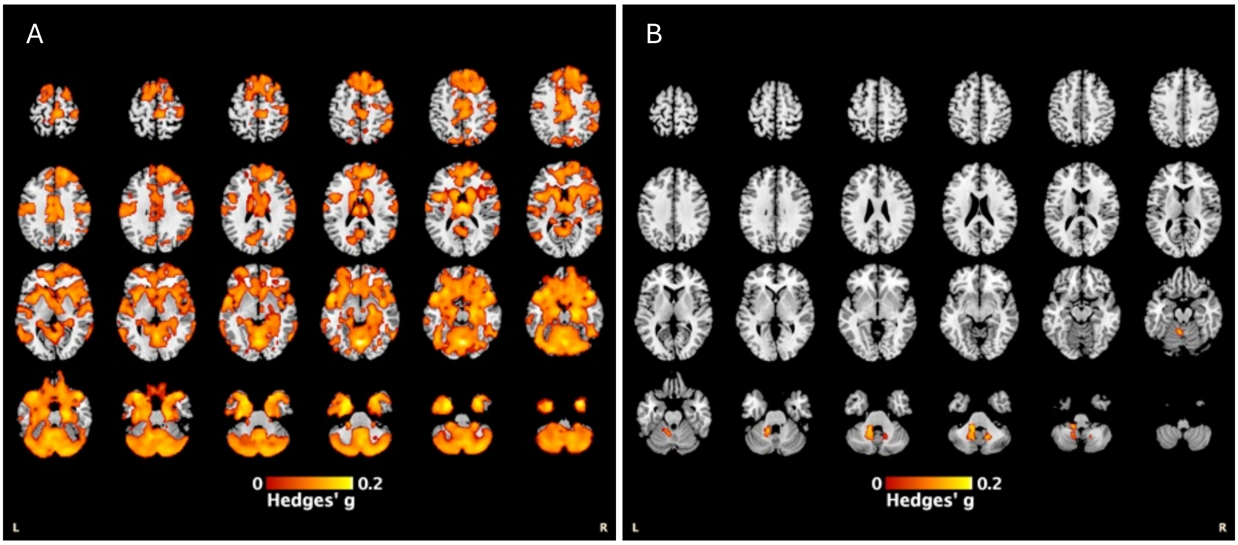

Table S15. PTSD vs. Controls excluding participants with traumatic brain injury

Peak Regions	MNI coordinate	Hedges' g	Z	Cluster size (voxels)	*p*-value[a]	I^2
**GREY MATTER** (Mean I^2 = 6.95 (all voxels in the brain))						
Right parahippocampus	22,-18,-24	0.23	5.261	65,592	.001	0.00
Right postcentral gyrus	48,-20,56	0.14	3.292	696	.010	0.00
Left postcentral gyrus	-46,-12,36	0.15	3.441	489	.008	0.00
Left postcentral gyrus	-66,-14,14	0.13	2.976	95	.021	0.00
Left inferior temporal gyrus	60,-14,-28	0.13	2.902	94	.021	0.00
**WHITE MATTER** (Mean I^2 = 4.36 (all voxels in the brain))						
Corpus callosum	-2,18,4	-0.18	-4.106	3,015	.002	0.00
Corpus callosum	-18,-20,60	-0.16	-3.3696	13	.025	15.83

[a]p-values have been FWE-corrected for multiple comparisons
Data included in the analysis comprised 927 PTSD patients and 1,603 controls from 33 cohorts. See Table S28 for the results of the correlation analysis.

Figure S10. PTSD vs. Controls excluding participants with traumatic brain injury – differences in GM and WM
(A) **Grey Matter**: Patients exhibited similar results to the main case-control finding with smaller GM volumes than controls.
(B) **White Matter**: Patients exhibited greater WM volumes than controls.

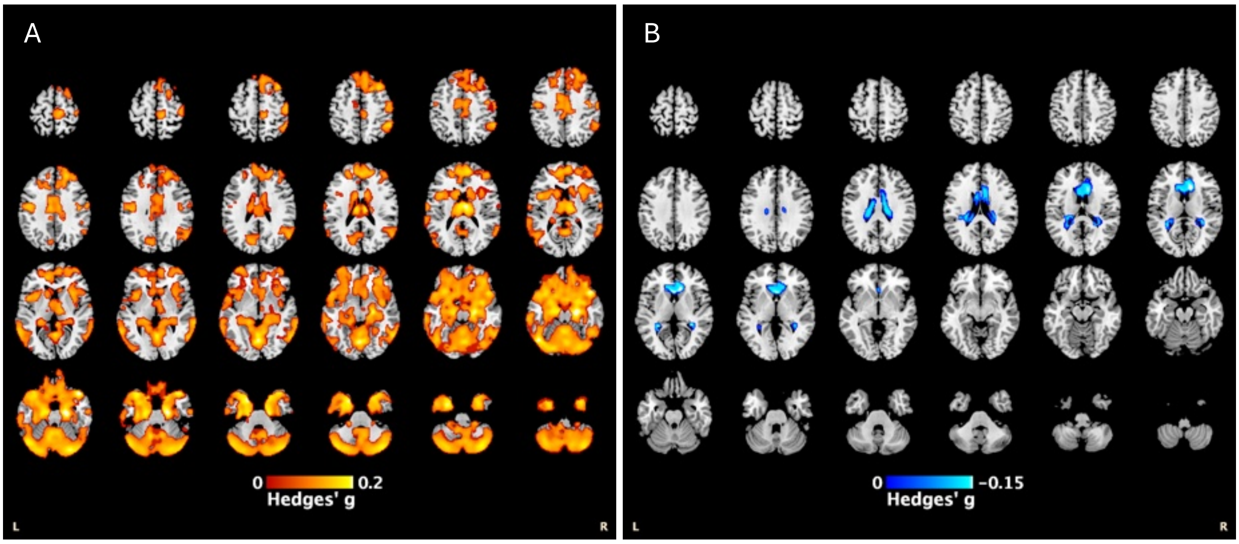

Sensitivity Analyses: controlling for different covariates

The sensitivity analyses compared patients and controls and included 35 cohorts consisting of 1,309 patients and 2,130 controls, controlling for different covariate combinations. The exception was when sex was included as a covariate, which included 32 cohorts consisting of 1,228 patients and 1,962 controls. 3 cohorts were excluded because they were single-sex samples.

In summary, the results for GM differences were similar to the main group finding (all Pearson's $r > .86$; see Table S28) when controlling for different covariate combinations. However, the significance of clusters changed when the model adjusted for total GM (instead of ICV), which revealed significant differences within the cerebellum only. Similarly, the resulting effect size maps for WM covarying for different covariate combinations were highly correlated to the main findings (all Pearson's $r > .9$), except when controlling for age and sex (Pearson's r = 0.78). The statistical significance of the clusters was, however, affected: WM differences were no longer significant when we controlled for age, age^2, ICV, and sex (Table S18, Figure S13). Furthermore, when using proportional scaling but covarying only for age and ICV, patients exhibited significantly greater WM volumes than controls within the corpus callosum (Table S21, Figure S16).

Table S16. Covarying for age, ICV, and sex

Peak Regions	MNI coordinate	Hedges' g	Z	Cluster size (voxels)	*p*-value[a]	I^2
**GREY MATTER** (Mean I^2 = 7.89 (all voxels in the brain))						
Left cerebellum	-4,-72,-12	0.22	5.858	90,572	.001	0.00
Right precuneus	10,-76,42	0.13	3.463	300	.017	0.17
Right precuneus	10,-58,48	0.10	2.770	83	.020	0.00
Left precuneus	-12,-78,46	0.09	2.481	66	.022	0.00
Left inferior parietal gyri	-34,-72,42	0.10	2.771	24	.022	0.00
**WHITE MATTER** (Mean I^2 = 4.50 (all voxels in the brain))						
Left cerebellum	-6,-54,-20	0.14	3.630	50	.020	1.59

[a]p-values have been FWE-corrected for multiple comparisons
Data included in the analysis comprised 1,228 PTSD and 2,025 controls from 32 cohorts.

Figure S11. Covarying for age, ICV, and sex – GM and WM differences
(A) **Grey Matter**: smaller GM volumes in patients compared to controls.
(B) **White Matter**: smaller WM volumes in patients compared to controls.

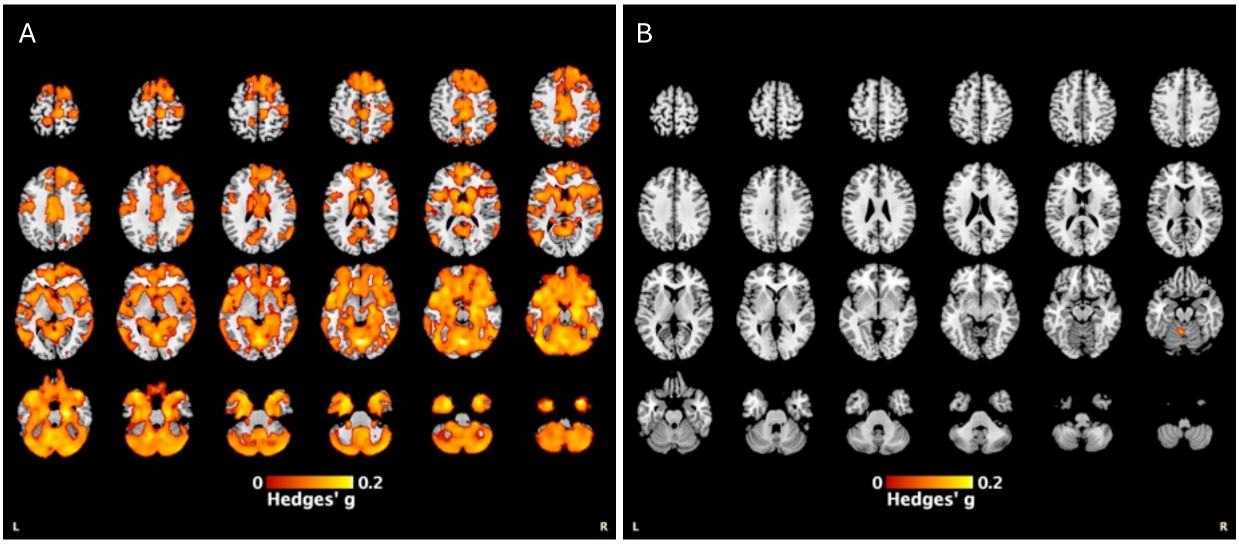

Table S17. Covarying for age and total GM or total WM

Peak Regions	MNI coordinate	Hedges' g	Z	Cluster size (voxels)	*p*-value[a]	I^2
**GREY MATTER** (Mean I^2 = 5.26 (all voxels in the brain))						
Right cerebellum	16,-58,-56	0.14	3.698	1,984	.009	0.00
Cerebellum vermis	-2,-72,-10	0.19	5.222	586	.003	1.67
**WHITE MATTER** (Mean I^2 = 2.66 (all voxels in the brain))						
Left cerebellum	-16,-54,-38	0.16	4.323	6,678	.001	2.31
WM adjacent to the left striatum	-30,-12,0	0.15	4.116	116	.012	0.00

[a]p-values have been FWE-corrected for multiple comparisons
Data included in the analysis comprised 1,309 PTSD and 2,130 controls from 35 cohorts.

Figure S12. Covarying for age and total GM or total WM – GM and WM differences
(A) **Grey Matter**: smaller GM volumes in patients compared to controls.
(B) **White Matter**: smaller WM volumes in patients compared to controls.

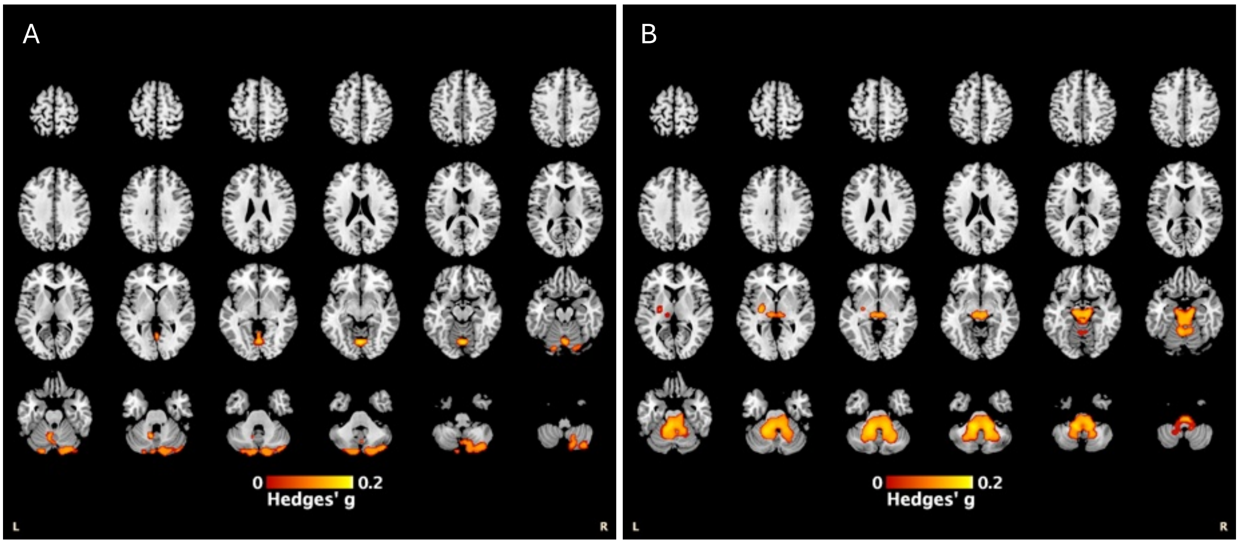

Table S18. Covarying for age, age^2, ICV, and sex

Peak Regions	MNI coordinate	Hedges' g	Z	Cluster size (voxels)	*p*-value[a]	I^2
**GREY MATTER** (Mean I^2 = 7.88 (all voxels in the brain))						
Left cerebellum	-4,-72,-12	0.21	5.472	87,346	.001	0.00
Right precuneus	8,-78,44	0.13	3.224	17	.024	6.22
Right superior parietal gyrus	18,-52,66	0.11	2.930	16	.025	1.93
**WHITE MATTER**						
No significant differences.						

[a]p-values have been FWE-corrected for multiple comparisons
Data included in the analysis comprised 1,228 PTSD and 2,025 controls from 32 cohorts.

Figure S13. Covarying for age, age^2, ICV, and sex – GM differences
Grey Matter: smaller GM volumes in patients compared to controls.

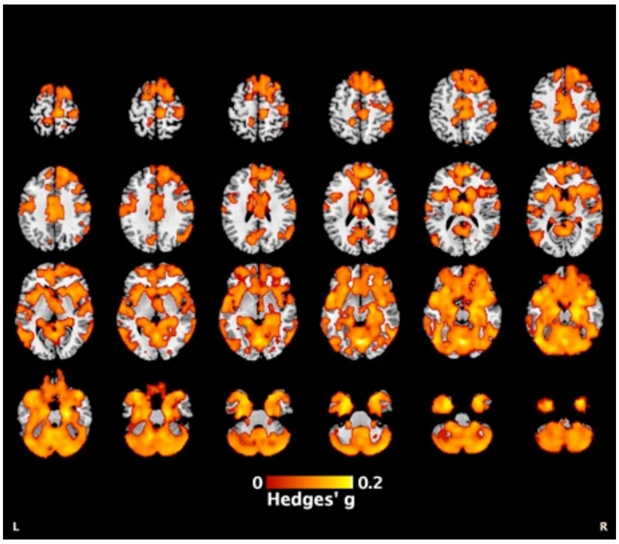

Table S19. Covarying for age and sex

Peak Regions	MNI coordinate	Hedges' g	Z	Cluster size (voxels)	*p*-value[a]	I^2
**GREY MATTER** (Mean I^2 = 8.28 (all voxels in the brain))						
Large cluster comprising regions across the frontal lobe, temporal lobe, cerebellum, parietal lobe, and thalamus.				139,985		
Left cerebellum	-4,-72,-12	0.24	6.259	Subcluster	< .001	0.00
Right parahippocampus	22,-18,-24	0.21	5.681	Subcluster	.001	0.00
Left fusiform	-34,-16,-34	0.21	5.527	Subcluster	.001	0.00
Left fusiform	-30,0,-42	0.22	5.435	Subcluster	.001	7.11
Cerebellum vermis	0,-62,-2	0.20	5.411	Subcluster	.001	0.00
**WHITE MATTER** (Mean I^2 = 1.85 (all voxels in the brain))						
Cluster across the cerebellum and striatum.				9,422		
Middle cerebellar peduncles	-16,-54,-38	0.15	4.024	Subcluster	< .001	1.30
WM adjacent to the left striatum	-30,-12,0	0.15	3.932	Subcluster	.001	0.00
WM adjacent to the right striatum	14,8,-6	0.15	3.918	Subcluster	.001	0.00
Left cerebellum	-6,-54,-20	0.14	3.76	Subcluster	.001	0.00
Middle cerebellar peduncles	14,-58,-42	0.15	3.75	Subcluster	.001	4.27

[a]p-values have been FWE-corrected for multiple comparisons
Data included in the analysis comprised 1,228 PTSD and 2,025 controls from 32 cohorts.

Figure S14. Covarying for age and sex – GM and WM differences
(A) **Grey Matter**: smaller GM volumes in patients compared to controls.
(B) **White Matter**: smaller WM volumes in patients compared to controls.

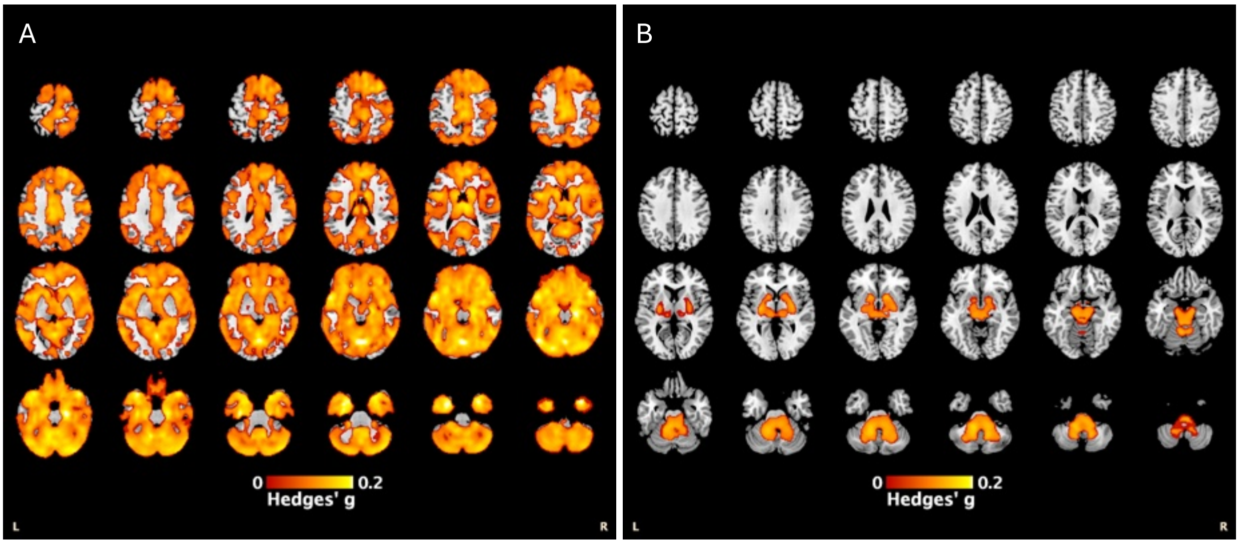

Table S20. Covarying for ICV

Peak Regions	MNI coordinate	Hedges' g	Z	Cluster size (voxels)	*p*-value[a]	I^2
**GREY MATTER** (Mean I^2 = 20.37 (all voxels in the brain))						
Large cluster comprising regions across the frontal lobe, temporal lobe, cerebellum, parietal lobe, and thalamus.				137,868		
Right parahippocampus	24,-18,-24	0.22	5.993	Subcluster	.001	0.00
Left cerebellum	-4,-70,-10	0.24	5.911	Subcluster	.001	14.41
Left lingual gyrus	-6,-74,-8	0.22	5.844	Subcluster	.001	2.33
Cerebellum vermis	0,-60,-2	0.21	5.593	Subcluster	.001	0.00
Right cerebellum	24,-82,-24	0.20	5.455	Subcluster	.001	0.00
**WHITE MATTER** (Mean I^2 = 4.51 (all voxels in the brain))						
Cluster within the cerebellum.				921		
Cerebellum vermis	-2,-54,-18	0.13	3.514	Subcluster	.017	0.00
Right cortico-spinal projections	4,-28,-48	0.13	3.403	Subcluster	.016	6.39
Cerebellum vermis	6,-52,-18	0.12	3.355	Subcluster	.020	0.00
Middle cerebellar peduncles	-14,-40,-42	0.14	3.198	Subcluster	.022	29.80
Left cerebellum	-16,-54,-38	0.12	3.166	Subcluster	.022	10.09

[a]p-values have been FWE-corrected for multiple comparisons
Data included in the analysis comprised 1,309 PTSD and 2,130 controls from 35 cohorts.

Figure S15. Covarying for ICV – GM and WM differences
(A) **Grey Matter**: smaller GM volumes in patients compared to controls.
(B) **White Matter**: smaller WM volumes in patients compared to controls.

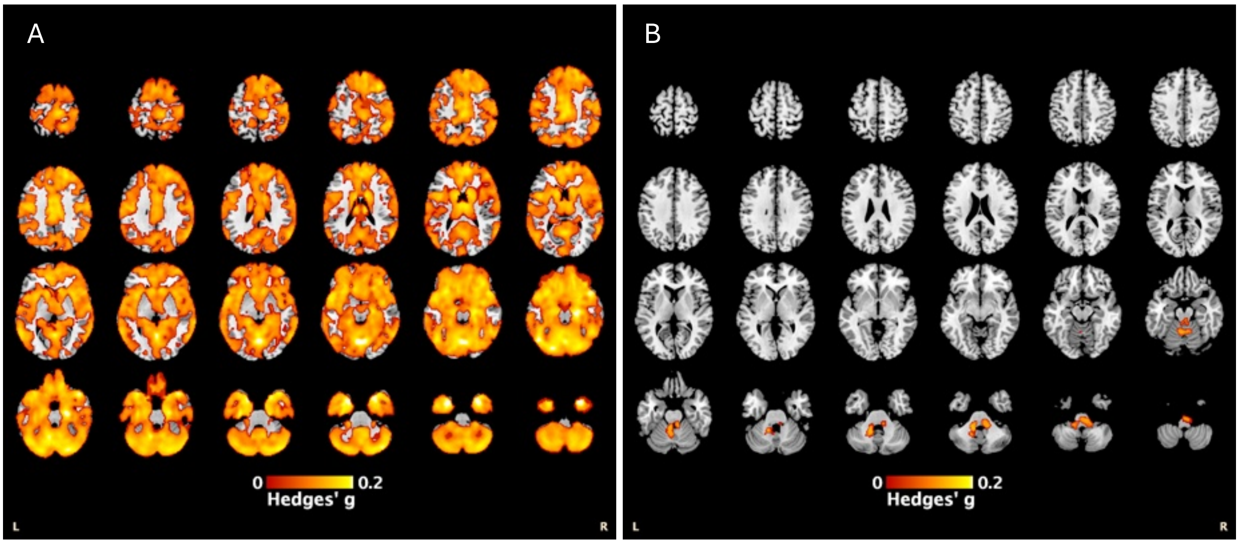

Table S21. Proportional scaling covarying for age and ICV

Peak Regions	MNI coordinate	Hedges' g	Z	Cluster size (voxels)	*p*-value[a]	I^2
**GREY MATTER** (Mean I^2 = 7.90 (all voxels in the brain))						
Large cluster comprising regions across the frontal lobe, temporal lobe, cerebellum, and thalamus.				73,902		
Right fusiform gyrus	28,2,-50	0.20	5.455	Subcluster	.001	2.55
Cerebellum vermis	-2,-72,-10	0.20	5.418	Subcluster	.001	0.00
Left fusiform gyrus	-34,-18,-34	0.20	5.391	Subcluster	.001	0.00
Left inferior temporal gyrus	-30,-2,-44	0.20	5.330	Subcluster	.001	5.68
Right parahippocampus	22,-16,-24	0.18	4.898	Subcluster	.001	0.00
**WHITE MATTER** (Mean I^2 = 6.44 (all voxels in the brain))						
Cluster within the corpus callosum.				849		
Corpus callosum	8,16,14	-0.14	-3.710	Subcluster	.006	0.00
Corpus callosum	10,0,22	-0.13	-3.408	Subcluster	.007	0.00
Corpus callosum	2,18,6	-0.12	-3.333	Subcluster	.006	1.54
Corpus callosum	14,-20,24	-0.12	-3.312	Subcluster	.009	0.00
Corpus callosum	-2,4,18	-0.11	-3.083	Subcluster	.015	0.00

[a]p-values have been FWE-corrected for multiple comparisons
Data included in the analysis comprised 1,309 PTSD and 2,130 controls from 35 cohorts.
Proportional scaling is where each voxel is scaled by the fraction of total ICV.

Figure S16. Proportional scaling covarying for age and ICV – GM and WM differences
(A) **Grey Matter**: smaller GM volumes in patients compared to controls.
(B) **White Matter**: greater WM volumes in patients compared to controls.

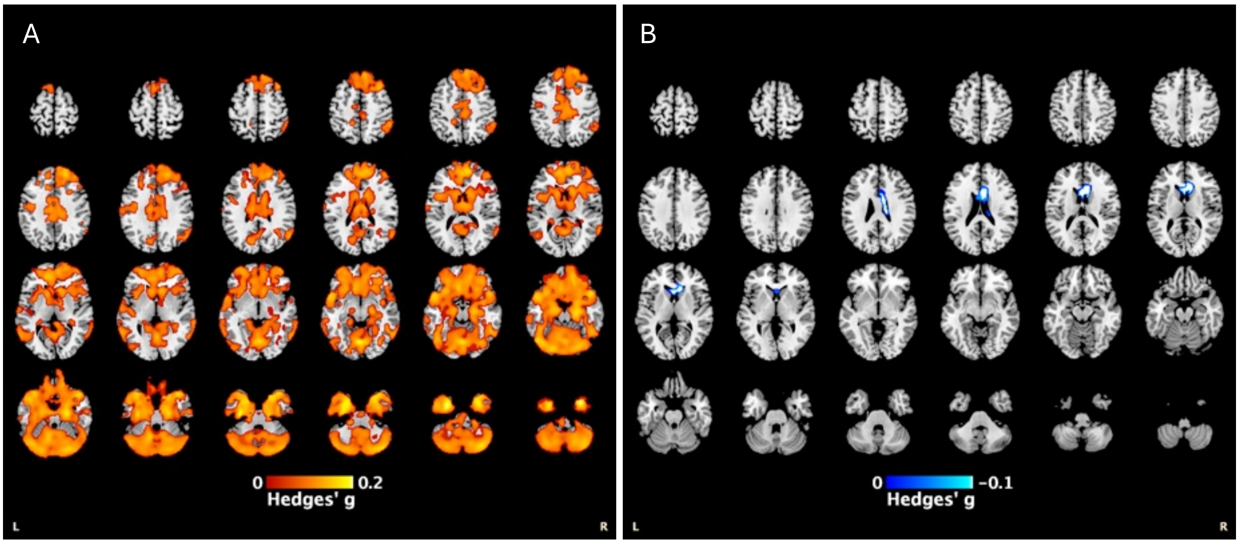

Table S22. No covariates

Peak Regions	MNI coordinate	Hedges' g	Z	Cluster size (voxels)	*p*-value[a]	I^2
**GREY MATTER** (Mean I^2 = 22.55 (all voxels in the brain))						
Large cluster widespread across the whole brain.				174,008		
Right parahippocampus	18,-22,-18	0.22	6.064	Subcluster	.001	0.00
Left lingual gyrus	-8,-74,-8	0.21	5.806	Subcluster	.001	0.00
Left cerebellum	-4,-72,-10	0.26	5.755	Subcluster	.001	27.63
Cerebellum vermis	0,-60,-2	0.21	5.713	Subcluster	.001	0.00
Right cerebellum	20,-84,-24	0.21	5.683	Subcluster	.001	2.59
**WHITE MATTER** (Mean I^2 = 3.22 (all voxels in the brain))						
Left cerebellum	-16,-54,-38	0.14	3.576	6,978	.002	6.55
WM adjacent to the right striatum	14,8,-6	0.13	3.495	224	.014	0.00
WM adjacent to the left striatum	-30,-12,0	0.13	3.481	72	.015	0.00
Left anterior thalamic projections	-14,10,-4	0.12	3.200	12	.025	0.00

[a]p-values have been FWE-corrected for multiple comparisons
Data included in the analysis comprised 1,309 PTSD and 2,130 controls from 35 cohorts.

Figure S17. No covariates – GM and WM differences
(A) **Grey Matter**: smaller GM volumes in patients compared to controls.
(B) **White Matter**: smaller WM volumes in patients compared to controls.

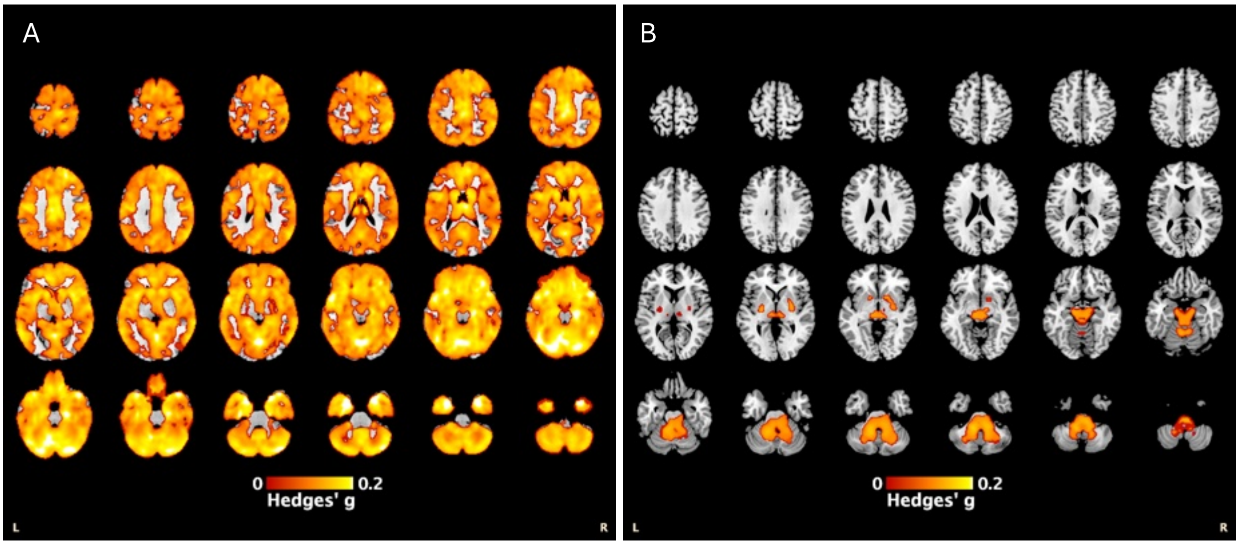

Sensitivity Analyses: using non-modulated images

Modulation is a process that aims to preserve brain volumes during the normalisation step in the VBM process. Warping effects can occur during normalisation as the image is being normalised to the MNI template. Modulation ensures each voxel represents the true volume as it takes into account the amount that each voxel has been dilated or compressed. The sensitivity analyses in this section used images that were not modulated during the normalisation step.

The non-modulated results appeared to have more widely spread clusters in the frontal regions, with less effects detected in the cerebellum for GM (Pearson's r = 0.60 to 0.61; see Table S28). Contrasting the main group results comparing WM differences, the non-modulated images revealed that patients exhibited greater WM volumes than controls in small clusters within the cingulum and longitudinal fasciculus (Pearson's r = 0.68 to 0.74; see Table S28).

Table S23. Non-modulated images covarying for age and ICV

Peak Regions	MNI coordinate	Hedges' g	Z	Cluster size (voxels)	*p*-value[a]	I^2
**GREY MATTER** (Mean I^2 = 9.99 (all voxels in the brain))						
Left olfactory	-4,22,0	0.21	5.745	70,986	< .001	0.00
Right cerebellum	24,-34,-44	0.13	3.548	16	.023	0.24
**WHITE MATTER** (Mean I^2 = 4.30 (all voxels in the brain))						
Left median cingulum network	-16,46,8	-0.16	-4.369	154	.007	0.14
Right median cingulum network	8,-16,36	-0.13	-3.673	119	.017	0.00
Left uncinate fasciculus	-18,28,-18	-0.15	-4.152	101	.009	0.22
Left median cingulate network	-10,-18,44	-0.15	-4.006	90	.013	0.00
Right frontal orbito-polar tract	16,40,-20	-0.16	-4.363	45	.017	0.00

[a]p-values have been FWE-corrected for multiple comparisons
Data included in the analysis comprised 1,309 PTSD and 2,130 controls from 35 cohorts.

Figure S18. Non-modulated images covarying for age and ICV – GM and WM differences
(A) **Grey Matter**: smaller GM volumes in patients compared to controls.
(B) **White Matter**: greater WM volumes in patients compared to controls.

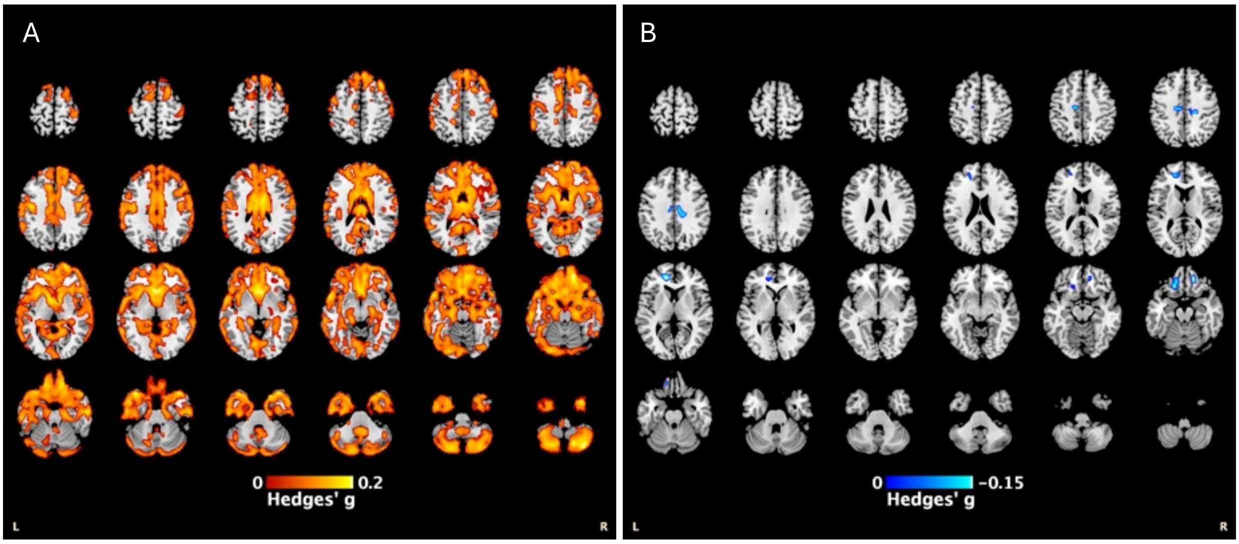

Table S24. Non-modulated images with no covariates

Peak Regions	MNI coordinate	Hedges' g	Z	Cluster size (voxels)	*p*-value[a]	I^2
**GREY MATTER** (Mean I^2 = 24.32 (all voxels in the brain))						
Left frontal superior gyrus	-16,38,-22	0.22	5.972	108,124	.001	0.00
Left paracentral lobule	-8,-40,78	0.15	3.184	40	.022	37.70
**WHITE MATTER** (Mean I^2 = 7.13 (all voxels in the brain))						
Left median cingulum	-14,44,8	-0.18	-4.855	5903	.002	1.29
Right superior longitudinal fasciculus III	42,-6,34	-0.16	-4.006	154	.011	11.40
Left superior longitudinal fasciculus II	-38,-8,32	-0.18	-4.991	118	.010	0.00
WM adjacent to the right inferior temporal gyrus	58,-14,-26	-0.17	-4.663	64	.012	0.00
Right frontal superior longitudinal fasciculus	20,16,46	-0.16	-4.229	27	.019	2.01

[a]p-values have been FWE-corrected for multiple comparisons
Data included in the analysis comprised 1,309 PTSD and 2,130 controls from 35 cohorts.

Figure S19. Non-modulated images with no covariates – GM and WM differences
(A) **Grey Matter**: smaller GM volumes in patients compared to controls.
(B) **White Matter**: greater WM volumes in patients compared to controls.

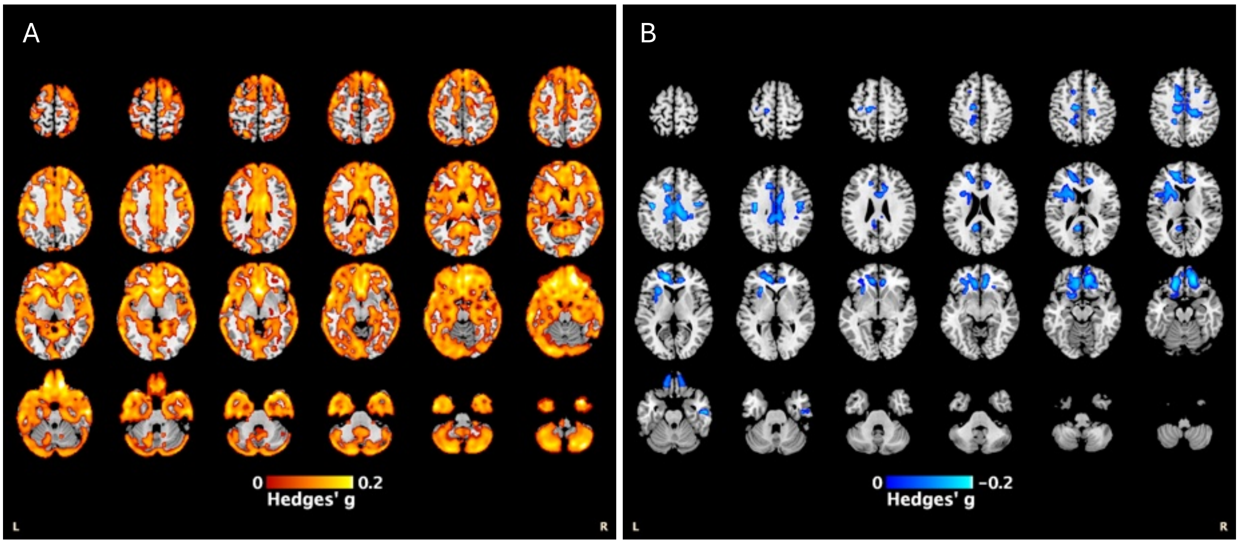

Sensitivity Analyses: varying smoothing kernel size

The analyses here repeated the main group comparison between patients and controls but using images that have been smoothed using different Gaussian kernels ranging between 2mm to 12mm. The main analysis was smoothed with an 8mm kernel.

Using 2mm, 4mm, or 12mm smoothing kernels exhibited effect size maps that were strongly correlated with the main group comparison (8mm smoothing kernel) for GM and WM (all Pearson's $r > .9$; see Table S28). However, the spatial extent of the significant clusters appeared to decrease with smaller kernel sizes and increase with the 12mm kernel size in the GM analysis. In the WM analysis, there were no significant differences between PTSD patients and controls when using the smaller kernel sizes of 2mm and 4mm. When using the 12mm kernel, the pattern of significant clusters exhibited a greater spatial extent (Table S27, Figure S22).

Table S25. Smoothing kernel of 2mm covarying for age and ICV

Peak Regions	MNI coordinate	Hedges' g	Z	Cluster size (voxels)	*p*-value[a]	I^2
**GREY MATTER** (Mean I^2 = 3.60 (all voxels in the brain))						
Cerebellum vermis	0,-70,-16	0.21	5.712	11,068	.001	0.00
Left fusiform gyrus	-34,-16,-34	0.21	5.652	2674	.002	0.00
Right caudate	14,12,12	0.16	4.187	467	.010	5.89
Right inferior frontal gyrus	38,32,-20	0.17	4.257	42	.021	9.91
Right superior frontal gyrus	12,34,-24	0.14	3.703	40	.021	0.00
**WHITE MATTER**						
No significant differences.						

[a]p-values have been FWE-corrected for multiple comparisons
Data included in the analysis comprised 1,309 PTSD and 2,130 controls from 35 cohorts.

Figure S20. Smoothing kernel of 2mm covarying for age and ICV – GM differences
Grey Matter: smaller GM volumes in patients compared to controls.

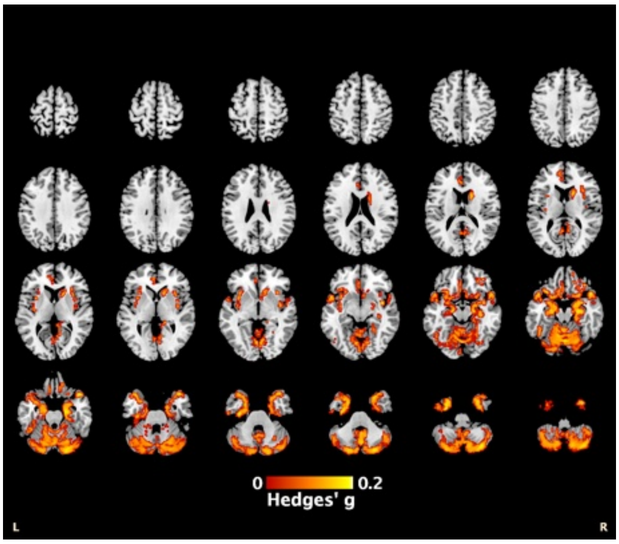

Table S26. Smoothing kernel of 4mm covarying for age and ICV

Peak Regions	MNI coordinate	Hedges' g	Z	Cluster size (voxels)	*p*-value[a]	I^2
**GREY MATTER** (Mean I^2 = 4.52 (all voxels in the brain))						
Cerebellum vermis	-2,-72,-14	0.20	5.561	40,655	.001	0.00
Right paracentral lobule	12,-26,66	0.15	4.101	1,333	.010	0.00
Right postcentral gyrus	60,-12,34	0.13	3.454	325	.019	0.00
Left middle temporal gyrus	-60,-54,2	0.15	4.189	253	.010	0.00
Right postcentral gyrus	42,-18,36	0.12	3.292	38	.024	0.00
**WHITE MATTER**						
No significant differences.						

[a]p-values have been FWE-corrected for multiple comparisons
Data included in the analysis comprised 1,309 PTSD and 2,130 controls from 35 cohorts.

Figure S21. Smoothing kernel of 4mm covarying for age and ICV – GM differences
Grey Matter: smaller GM volumes in patients compared to controls.

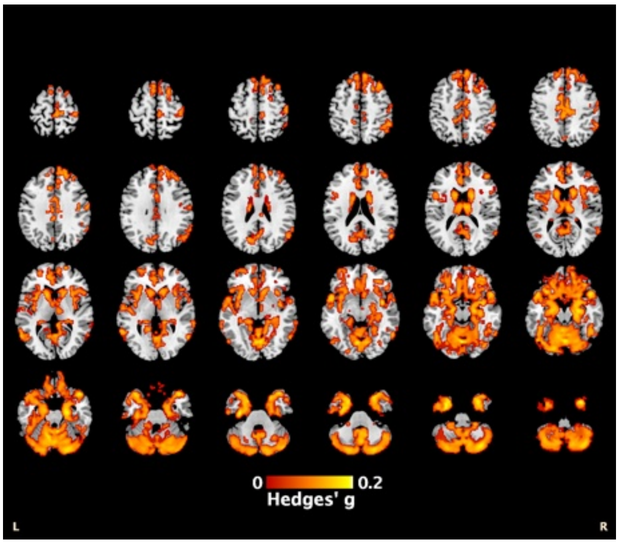

Table S27. Smoothing kernel of 12mm covarying for age and ICV

Peak Regions	MNI coordinate	Hedges' g	Z	Cluster size (voxels)	*p*-value[a]	I^2
**GREY MATTER** (Mean I^2 = 10.76 (all voxels in the brain))						
Large cluster comprising regions across the frontal lobe, temporal lobe, cerebellum, and thalamus.				123,254		
Cerebellum vermis	-2,-70,-12	0.22	5.765	Subcluster	.001	5.10
Right parahippocampus	20,-18,-24	0.20	5.516	Subcluster	.001	0.00
Left fusiform gyrus	-26,2,-48	0.20	5.271	Subcluster	.001	0.98
Left fusiform gyrus	-28,2,-38	0.19	5.252	Subcluster	.001	0.00
Left fusiform gyrus	-26,-2,-50	0.19	5.187	Subcluster	.001	0.00
**WHITE MATTER** (Mean I^2 = 7.47 (all voxels in the brain))						
Cluster across the cerebellum.				5,222		
Left cerebellum	-16,-56,-40	0.13	3.438	Subcluster	.003	0.48
Left cerebellum	-6,-52,-18	0.14	3.336	Subcluster	.003	18.03
Left cerebellum	-10,-50,-18	0.14	3.325	Subcluster	.003	21.28
Left cerebellum	-10,-50,-24	0.14	3.172	Subcluster	.003	24.92
Right cerebellum	14,-56,-40	0.12	3.101	Subcluster	.004	6.44

[a]p-values have been FWE-corrected for multiple comparisons
Data included in the analysis comprised 1,309 PTSD and 2,130 controls from 35 cohorts.

Figure S22. Smoothing kernel of 12mm covarying for age and ICV – GM and WM differences
(A) **Grey Matter**: smaller GM volumes in patients compared to controls.
(B) **White Matter**: smaller WM volumes in patients compared to controls.

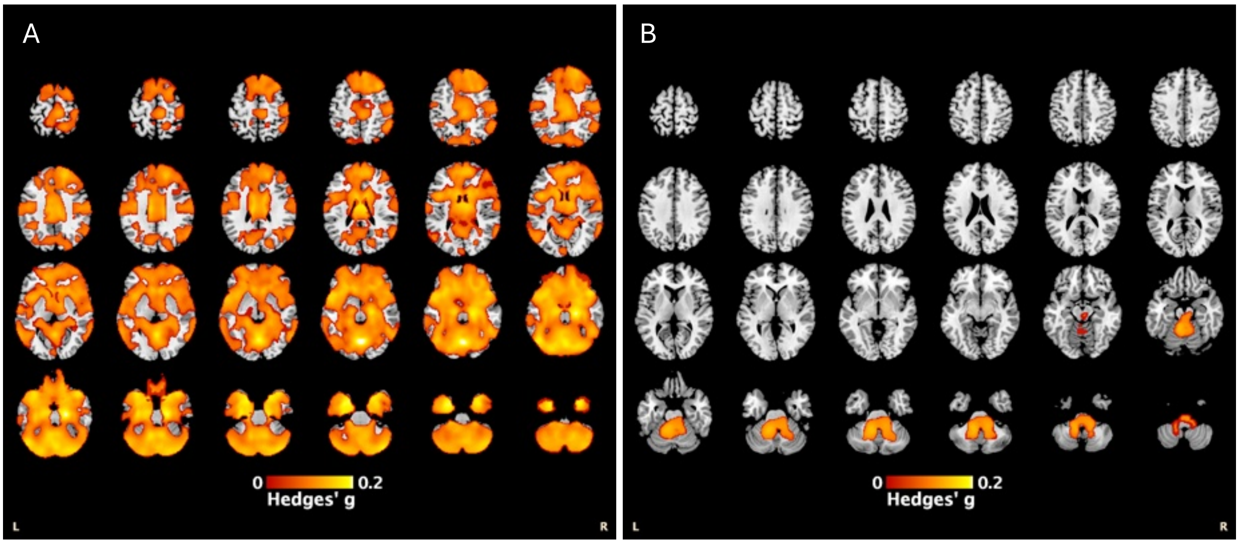

Sensitivity Analysis: comparison to the main results

Table S28. Correlations between the results of the sensitivity analyses and the main results.

	Pearson's *r*[a]	
**Sensitivity Analysis**	**Grey Matter**	**White Matter**
Sample exclusions		
Excluding non-adult cohorts	.989	.994
Excluding moderate/severe TBI participants	.963	.951
Controlling for different covariates		
Covarying age, ICV, and sex	.987	.990
Covarying age, total GM / WM	.873	.978
Covarying age, age squared, ICV, and sex	.988	.972[b]
Covarying age and sex	.971	.775
Covarying ICV only	.927	.980
Proportional scaling, covarying age and ICV	.918	.945
No covariates	.934	.760
Non-modulated images		
Non-modulated, covarying age and ICV	.558	.743
Non-modulated, no covariates	.491	.683
Varying smoothing kernel sizes		
Smoothing Kernel 2mm, covarying age and ICV	.943	.959[b]
Smoothing Kernel 4mm, covarying age and ICV	.983	.982[b]
Smoothing Kernel 12mm, covarying age and ICV	.985	.989

[a] Correlation analysis is performed between the resulting effect size maps from the sensitivity analysis and the main group comparison using a parcel-based correlation approach. Voxels that had zero value in both maps were excluded.
[b] No significant clusters were observed in the sensitivity analysis.

Heterogeneity of Effect Size

Figure S23. The heterogeneity of effect size measured using the I^2 statistic for the main group comparison.
(A) **Grey Matter**: overall mean I^2 = 8.15% across all GM voxels in the brain.
(B) **White Matter**: overall mean I^2 = 4.67% across all WM voxels in the brain.

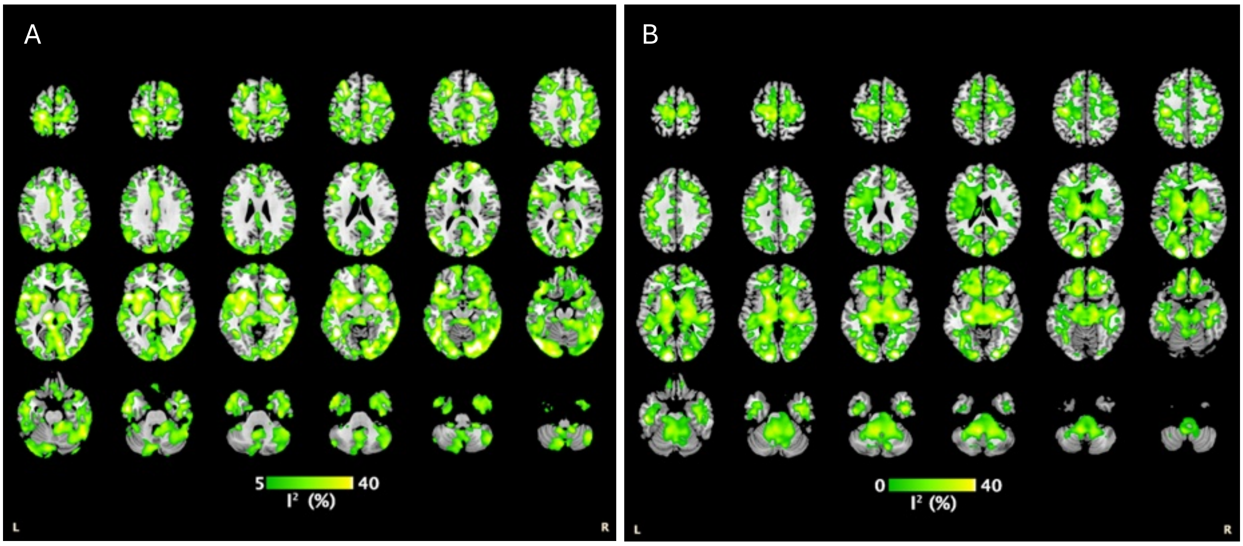

Supplementary References

1. Wang X, Xie H, Chen T, Cotton AS, Salminen LE, Logue MW, et al. Cortical volume abnormalities in posttraumatic stress disorder: an ENIGMA-psychiatric genomics consortium PTSD workgroup mega-analysis. Mol Psychiatry. 2021;26(8):4331-43. 10.1038/s41380-020-00967-1
2. Huggins AA, Baird CL, Briggs M, Laskowitz S, Hussain A, Fouda S, et al. Smaller total and subregional cerebellar volumes in posttraumatic stress disorder: a mega-analysis by the ENIGMA-PGC PTSD workgroup. Mol Psychiatry. 2024. 10.1038/s41380-023-02352-0
3. Saunders JB, Aasland OG, Babor TF, de la Fuente JR, Grant M. Development of the Alcohol Use Disorders Identification Test (AUDIT): WHO Collaborative Project on Early Detection of Persons with Harmful Alcohol Consumption--II. Addiction. 1993;88(6):791-804. 10.1111/j.1360-0443.1993.tb02093.x
4. Skinner HA. The drug abuse screening test. Addict Behav. 1982;7(4):363-71. 10.1016/0306-4603(82)90005-3
5. Gavin DR, Ross HE, Skinner HA. Diagnostic validity of the drug abuse screening test in the assessment of DSM-III drug disorders. Br J Addict. 1989;84(3):301-7. 10.1111/j.1360-0443.1989.tb03463.x
6. Whitcher B, Schmid VJ, Thornton A. Working with the DICOM and NIfTI Data Standards in R. Journal of Statistical Software. 2011;44(6). 10.18637/jss.v044.i06
7. Fortea L, Ortuno M, De Prisco M, Oliva V, Albajes-Eizagirre A, Fortea A, et al. Atlas of gray matter volume differences across psychiatric conditions: A systematic review with a novel meta-analysis that considers co-occurring disorders. Biol Psychiatry. 2024. 10.1016/j.biopsych.2024.10.020
8. Blake DD, Weathers FW, Nagy LM, Kaloupek DG, Gusman FD, Charney DS, et al. The development of a Clinician-Administered PTSD Scale. J Trauma Stress. 1995;8(1):75-90. 10.1007/BF02105408
9. Weathers FW, Bovin MJ, Lee DJ, Sloan DM, Schnurr PP, Kaloupek DG, et al. The Clinician-Administered PTSD Scale for DSM-5 (CAPS-5): Development and initial psychometric evaluation in military veterans. Psychol Assess. 2018;30(3):383-95. 10.1037/pas0000486
10. Blevins CA, Weathers FW, Davis MT, Witte TK, Domino JL. The Posttraumatic Stress Disorder Checklist for DSM-5 (PCL-5): Development and Initial Psychometric Evaluation. J Trauma Stress. 2015;28(6):489-98. 10.1002/jts.22059
11. Silverman WK, Nelles WB. The Anxiety Disorders Interview Schedule for Children. J Am Acad Child Adolesc Psychiatry. 1988;27(6):772-8. 10.1097/00004583-198811000-00019
12. First MB, Williams JBW, Karg RS, Spitzer RL. Structured Clinical Interview for DSM-5. Arlington, VA: American Psychiatric Association; 2015.
13. Sheehan DV, Lecrubier Y, Sheehan KH, Amorim P, Janavs J, Weiller E, et al. The Mini-International Neuropsychiatric Interview (M.I.N.I.): the development and validation of a structured diagnostic psychiatric interview for DSM-IV and ICD-10. Journal of Clinical Psychiatry. 1998;59:22-33.
14. Falsetti SA, Resnick HS, Resick PA, Kilpatrick DG. The Modified PTSD Symptom Scale: A brief self-report measure of posttraumatic stress disorder. The Behavior Therapist. 1993;16:161-2.
15. Briere J. Trauma Symptom Checklist for Children (TSCC), Professional Manual. Odessa, FL: Psychological Assessment Resources; 1996. 10.1037/t06631-000
16. Foa EB, McLean CP, Zang Y, Zhong J, Powers MB, Kauffman BY, et al. Psychometric properties of the Posttraumatic Diagnostic Scale for DSM-5 (PDS-5). Psychol Assess. 2016;28(10):1166-71. 10.1037/pas0000258
17. Yesavage JA, Sheikh JI. Geriatric Depression Scale (GDS). Clinical Gerontologist. 2008;5(1-2):165-73. 10.1300/J018v05n01_09

18. Zigmond AS, Snaith RP. The hospital anxiety and depression scale. Acta Psychiatr Scand. 1983;67(6):361-70. 10.1111/j.1600-0447.1983.tb09716.x
19. Beck AT, Steer RA, Brown G. Manual for the Beck Depression Inventory-II. San Antonio, TX: Psychological Corporation; 1996. 10.1037/t00742-000
20. Kroenke K, Spitzer RL, Williams JB. The PHQ-9: validity of a brief depression severity measure. J Gen Intern Med. 2001;16(9):606-13. 10.1046/j.1525-1497.2001.016009606.x
21. Kovacs M. Rating scales to assess depression in school-aged children. Acta Paedopsychiatrica: International Journal of Child & Adolescent Psychiatry. 2014;45(5-6):305-15. 10.1037/t19482-000
22. Radloff LS. The CES-D Scale. Applied Psychological Measurement. 1977;1(3):385-401. 10.1177/014662167700100306
23. Lovibond SH, Lovibond PF. Manual for the Depression Anxiety Stress Scales (2nd ed.). Sydney: Psychology Foundation; 1995. 10.1037/t01004-000
24. Watson D, Clark LA. Mood and Anxiety Symptom Questionnaire (MASQ). Iowa: University of Iowa; 1991. 10.1037/t13679-000
25. Bernstein DP, Stein JA, Newcomb MD, Walker E, Pogge D, Ahluvalia T, et al. Development and validation of a brief screening version of the Childhood Trauma Questionnaire. Child Abuse & Neglect. 2003;27(2):169-90. 10.1016/s0145-2134(02)00541-0
26. Bremner JD, Vermetten E, Mazure CM. Development and preliminary psychometric properties of an instrument for the measurement of childhood trauma: The early trauma inventory. Depress Anxiety. 2000;12:1-12. https://doi.org/10.1002/1520-6394(2000)12:1%3C1::AID-DA1%3E3.0.CO;2-W
