# Supplementary material for: A whole-brain voxel-based analysis of structural abnormalities in PTSD: An ENIGMA-PGC study
Source: Eur Psychiatry. 2025 Jul 22;68(1):e97. doi: 10.1192/j.eurpsy.2025.10062 (PMC12344465; doi:10.1192/j.eurpsy.2025.10062)
Supplement: See et al. supplementary material 2 — See et al. supplementary material [file S092493382510062Xsup002.pdf]

Supplement B

Overview of the ENIGMA-VBM Tool

The ENIGMA-PGC[1] is a collaboration between research sites to share neuroimaging data for meta-analysis. For a given study, it is not always possible to share participant-level data with the coordinating site due to data privacy restrictions. As such, the ENIGMA-VBM tool (https://sites.google.com/view/enigmavbm) has been developed to provide sites with an automated voxel-based morphometry (VBM) processing pipeline that enables them to process their data locally without having to share participant-level data with the coordinating site. The ENIGMA-VBM tool generates group-level data which is sent to the coordinating site for analysis. In doing so, it not only addresses concerns around data privacy, but the tool standardises the analytical pipeline ensuring that the meta-analysis is not confounded by different VBM parameters and settings during the processing stage.

Technical Information
This document is applicable to the ENIGMA-VBM tool version 1.076. The ENIGMA-VBM tool has been designed to work in MATLAB (backward compatible to MATLAB 2007) and calls on procedures from the software SPM12 (https://www.fil.ion.ucl.ac.uk/spm/). Figure B1 provides a summary overview of the process.

User Input
When the tool is launched, the software prompts the user for information about the cohort (PI name, contact, city). The tool will also check with the user if the sample is made up of participants of East Asian descent, and if so, it will use an appropriate template for affine transformation during later processing. It also requires the user to provide a covariates file in CSV (comma-separate values) file format. The covariates file has a prescribed format with each row corresponding to a patient or control, and the first four columns must contain the participant ID, diagnosis, age, and sex, in that order. Additional clinical covariates can be included in the following columns thereafter. Sites are provided with a manual on how to create the covariate file and what clinical covariates should be included where available.

The user will also be prompted by the tool to select the T1 images for patients and controls. It is recommended that cohorts include at least 10 patients and 8 controls. The tool will generate a random selection of T1 images for the user to verify the orientation of the scans.

Finally, the user will also be asked to supply a 'CNR.nii' file. This file is supplied with the ENIGMA-VBM tool and contains regions of interest (ROI) in the MNI space which is used to sample grey matter (GM), white matter (WM), cerebrospinal fluid (CSF), air, and bone. This information is used to calculate the contrast-to-noise ratio (CNR) for the cohort, which can be used by the coordinating site to assess image quality.

[1] Enhancing Neuro-Imaging Genetics through Meta-Analysis-Psychiatric Genomics Consortium

Data Processing

The tool utilises the standard DARTEL (Diffeomorphic Anatomical Registration Through Exponentiated Lie Algebra) VBM process [1]. In summary, the tool will: 1) *Segment* – individual T1 data is segmented to produce native space and DARTEL imported versions of GM and WM; 2) *Create Templates* – the DARTEL imported images are used to generate template images by iteratively aligning GM and WM across images. DARTEL adjusts three parameters for each voxel, which are then captured as flow fields for each participant that encode the movement for each voxel from the participant image to match the template; 3) *Normalise to MNI* – the flow fields are applied to the corresponding native space segmented image to normalise the image to the MNI space. During this stage, modulation is applied which ensures each voxel represents the true volume as it takes into account the amount that each voxel has been dilated or compressed during normalisation; 4) *Smoothing* – the normalised image is smoothed using a Gaussian kernel of 8mm FWHM (full width half maximum), and the final result is a smoothed, segmented image in the MNI space. Default parameters and settings are used unless otherwise stated.

The ENIGMA-VBM tool will also re-process scans using different VBM settings for sensitivity analyses such as using non-modulated images or using different smoothing kernel sizes.

Statistical Analysis

The main analysis performed by the ENIGMA-VBM tool is a group comparison between patients and controls, covarying for age and total intracranial volume (ICV). The tool uses SPM12 models for design specification, the default classical model estimation (using restricted maximum likelihood), and the SPM contrast manager in batch mode.

Regression analyses are conducted for each clinical variable in the covariates file within the patient group. As an example, the tool will investigate the association between GM volumes and symptom severity. For each regression, the tool will produce results covarying for age and ICV, as well as results covarying for age, ICV, and sex.

Sensitivity analyses test over a range of different parameters and settings during processing in SPM. In addition to using non-modulated processing and different smoothing kernel sizes (2mm, 4mm, and 12mm), the tool also tests for the use of proportional scaling, where each voxel is scaled by the fraction of total ICV rather than using ICV as a covariate, and covarying for different variables (e.g., total GM or WM, sex).

Integrated Quality Control

Several quality control (QC) steps are integrated into the ENIGMA-VBM tool. During the initial user input stage, the tool will prompt the user to check that the T1 images are oriented correctly, and it will identify if any participant scans have large voxel sizes, or if the voxel volume is significantly different between cases and controls, as this may affect processing. The tool will also identify if the sample is not well matched based on age or sex, or if there are any outlier values for age or other clinical covariate values. The tool will display the T1 file names and the participant IDs from the covariate file, prompting the user to check that these have been matched correctly. During these checks, the user will be presented with an option to continue or to terminate the process, allowing them to address any issues with the cohort before re-running the tool.

The tool will also detect whether any participants have extreme high or low GM or WM volumes which could indicate an error in the imaging and will automatically exclude images that exceed a specified threshold (GM volume <0.10 or >1.40 litres; WM volume <0.13 or >0.80 litres).

To check that the demographic data is correctly matched with the T1 images, the tool will test whether the mean male total ICV is greater than the mean female total ICV. This finding typically has a large effect size of Cohen's d = 1.3, and would be expected in large, adult MRI datasets. As additional checks, the tool will test if GM volumes reduce with age controlling for sex and for ICV separately. These checks are done on the entire sample, and separately for the patient and control groups.

The results from the QC checks are logged in a text file for review.

Output Files

The ENIGMA-VBM tool generates several output files. 2D screenshots of the T1 images are saved at each step in the VBM process and include initial defaced scans, segmented images, DARTEL templates, and smoothed modulated segmented images. These images are used to visually verify each participant was processed as expected. The tool generates a text file "shared_info.txt" which contains the details from the user inputs including information about the cohort (site, PI, contact) and sample (number of patients/controls, voxel sizes, summary statistics of the clinical covariates), metadata pertaining to the computer model, software versions, and the results of the QC checks as described above. The results of the main group comparison analysis, sensitivity analyses, and regression analyses are saved in corresponding folders for GM and WM and consist of the group statistical T-maps, PDF files detailing the design matrix used for the analysis, residuals images, contrast files, and beta files. This output is zipped into a single file for convenience to share with the coordinating site.

The tool also produces additional outputs which are not shared with the coordinating site. This includes log files and more detail at a participant-level which can be used for further error or QC troubleshooting as necessary. A mega-analysis folder is also created in case the site wishes to contribute the data for any mega-analysis. However, this data is at a participant-level and as such will not be shared with the coordinating site without explicit agreement.

Validation

The ENIGMA-VBM tool has been validated using 36 participants from the publicly available IXI dataset (https://brain-development.org/ixi-dataset/). The 36 IXI participants were randomly split into 'patient' and 'control' groups. All processing was done manually in SPM12 in parallel with the ENIGMA-VBM tool. The outputs from each processing step (i.e., segmentation, template creation, normalisation) were compared between the manual process and the output from the ENIGMA-VBM tool using Pearson's correlation, where all image outputs had a correlation coefficient $r \geq 0.999$. The summary of the demographic and clinical variables matched between the tool's summary and a manually calculated summary.

Meta-Analysis

The group-level data were meta-analysed using the software Seed Based-d Mapping with Permutation of Subject Image (SDM-PSI) version 6.22 (https://www.sdmproject.com). SDM-PSI involves four main steps: 1) *Pre-processing* – registering the T-maps to the SDM template (in MNI

space) and converting t-values to effect sizes; 2) *Mean Analysis* – calculating the mean of voxel values via random-effects meta-analysis; 3) *Family-Wise Error (FWE) Correction* – the p-values are corrected using a subject-based permutation test using threshold-free cluster enhancement (TFCE); 4) *Threshold* – the results are thresholded to obtain peak coordinates and cluster breakdowns. Standard settings were used at all stages except during pre-processing, where a whole brain mask was applied. The statistical threshold was set at $p < .025$ with a minimum cluster size of 10 voxels.

SDM requires that the statistical T-maps for pooling (from the ENIGMA-VBM tool output) be organised into a separate folder per analysis and accompanied by a text file ("sdm_table.txt") containing the name of the cohort (corresponding to the filename of the T-map), and the number of cases and controls. The coordinating site used an ENIGMA-VBM Extractor Tool, as developed by the authors, which automatically extracts the statistical T-maps, groups the T-maps into separate folders for each analysis, and generates the sdm_table.txt file. The Extractor Tool also creates several CSV files containing key information from the shared_info.txt files from each site, which allowed the coordinating site to review the clinical characteristics of the full sample, the results from the quality control checks, and other useful meta-data.

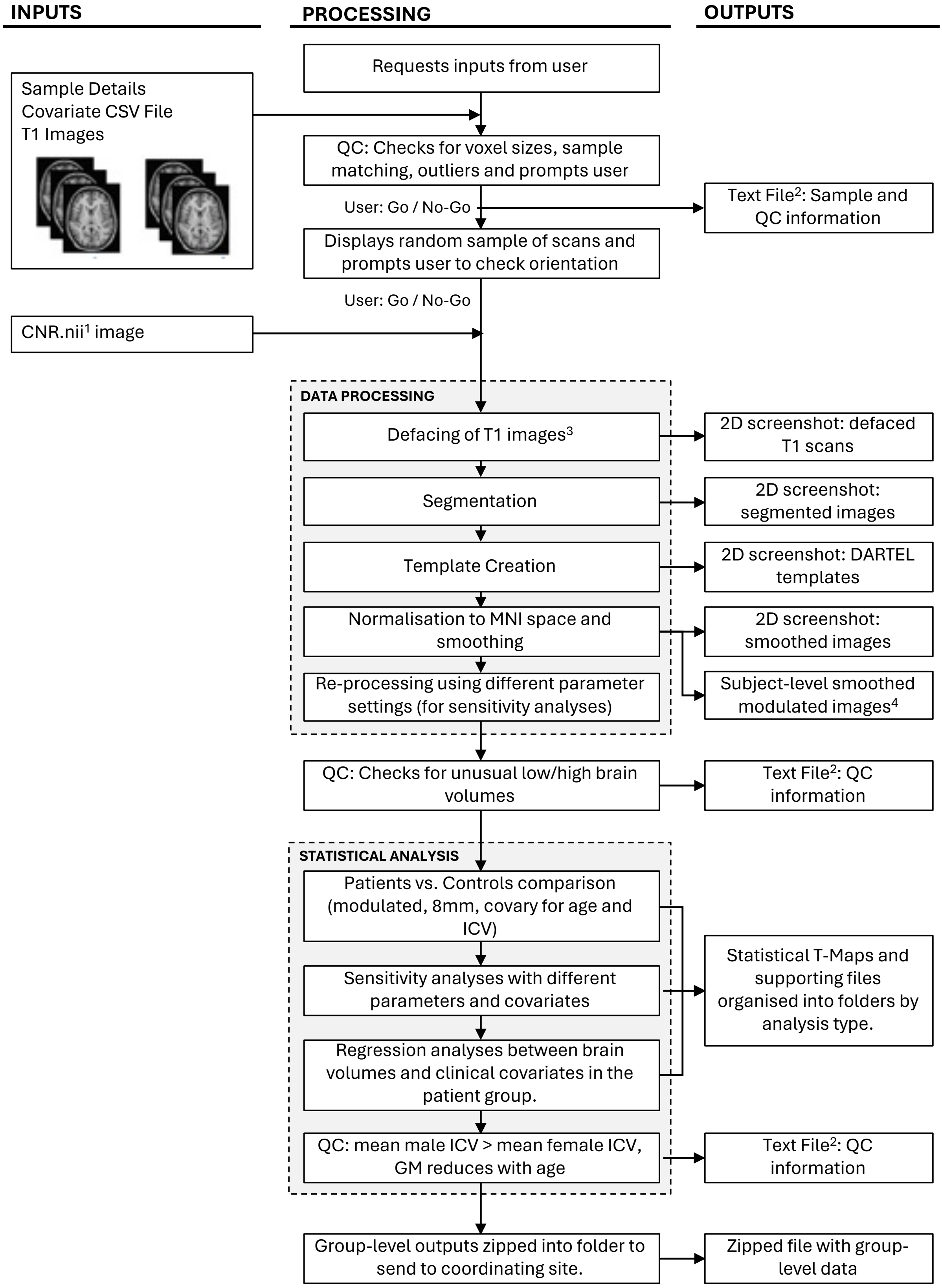

[1]The CNR.nii file is supplied with the tool and is used to sample grey matter, white matter, cerebrospinal fluid, air, and bone.
[2]The text file is a single file called "shared_info.txt".
[3]Defacing of the T1 images is only for the purposes of the 2D screenshot used for quality control. Segmentation is performed on the full image.
[4]The participant-level images are for an optional mega-analysis. This is not sent to the coordinating site.

Figure B1. Summary overview of the ENIGMA-VBM tool.

References

1. Ashburner J. A fast diffeomorphic image registration algorithm. Neuroimage. 2007;38(1):95-113. 10.1016/j.neuroimage.2007.07.007
